# The association between primary frozen shoulder and serum lipids may be overestimated: evidence based on retrospective observational studies and Mendelian randomization

**DOI:** 10.3389/fendo.2024.1363018

**Published:** 2025-01-15

**Authors:** Yongchao Zhang, Wenhao Fan, Yichang Wang, Tengjing Dong, Deding Liu, Yiming Shao

**Affiliations:** ^1^ Department of Joint and Sports Injuries, Zhengzhou Central Hospital Affiliated to Zhengzhou University, Zhengzhou, China; ^2^ Department of Foot and Ankle Surgery, Zhengzhou Orthopedic Hospital Affiliated to Henan University, Zhengzhou, China; ^3^ Department of Pain, Shunde Hospital of Southern Medical University, Foshan, China; ^4^ Department of Orthopedics, The Second Affiliated Hospital of Zhengzhou University, Zhengzhou, China; ^5^ Department of Trauma Surgery, Zhengzhou Central Hospital Affiliated to Zhengzhou University, Zhengzhou, China

**Keywords:** primary frozen shoulder, serum lipid, Mendelian randomization, correlation, causality

## Abstract

**Background:**

Previous studies have shown that dyslipidemia is significantly associated with primary frozen shoulder and may be a risk factor for the development of primary frozen shoulder. However, these findings may be biased by a number of confounding factors. We investigated the association between serum lipids and primary frozen shoulder by retrospective analysis and two-sample Mendelian randomization (MR) methods.

**Methods:**

This retrospective observational study included 284 patients with primary frozen shoulder diagnosed from October 2020 to October 2023 at four centers as the experimental group. Patients with diabetes and thyroid dysfunction were excluded. The control group consisted of age- and sex-matched people who underwent a health checkup. We compared total cholesterol (TC), triglycerides (TG), high-density lipoprotein (HDL), and low-density lipoprotein (LDL) between the two groups. Genetic variants for the serum lipids and frozen shoulder were then extracted from large-scale genome-wide association studies. Causal effects were assessed using Inverse variance weighting (IVW), Weighted median, MR-Egger, simple and weighted models in MR analysis.

**Results:**

The analysis showed that after excluding patients with diabetes and thyroid dysfunction, the serum lipids (TC, TG, HDL, and LDL) in the primary frozen shoulder group were no different from those of normal individuals. None of the MR methods found significant causal evidence between them.

**Conclusions:**

Dyslipidemia in patients with primary frozen shoulder may be influenced by confounding factors such as diabetes and thyroid dysfunction. These findings deepen our understanding of primary frozen shoulder risk factors.

## 1 Introduction

For Primary frozen shoulder, also known as “adhesive capsulitis of the shoulder”, is a common shoulder disorder characterized by spontaneous pain and limited shoulder motion (1). The major risk factors for primary frozen shoulder that have been reported include age, female gender, diabetes mellitus, and thyroid diseases (2–4). Because the mechanisms of primary frozen shoulder development are unknown, identifying possible risk factors is particularly important for the diagnosis and clinical management of primary frozen shoulder.

Bunker’s study (5) provided the first indication that dyslipidemia may be a risk factor for the pathogenesis of frozen shoulder. This was based on the finding that the pathologic manifestations of frozen shoulder are very similar to Dupuytren’s disease, which is known to be associated with hyperlipidemia. Subsequently Bunker et al. (6) further found that fasting serum triglyceride (TG) and cholesterol levels were significantly higher in patients with frozen shoulder than in healthy controls. However, the significance of this study is limited by the fact that the effects of confounding factors (such as diabetes mellitus and thyroid dysfunction) were not considered. Dyslipidemia is frequent in patients with diabetes mellitus and thyroid disease, and it is not possible to identify dyslipidemia as an independent risk factor for primary frozen shoulder (7, 8). With this in mind, Sung et al. (9) conducted a case-control study after excluding patients with diabetes mellitus and thyroid dysfunction. Although the results had significance in continuous and categorical values, the actual differences between the two study groups of these serum lipid levels were small. The relationship between primary frozen shoulder and dyslipidemia remains ambiguous, therefore strong evidence of a correlation is needed. And to the best of our knowledge, there has not been a study exploring the causal relationship between primary frozen shoulder and dyslipidemia.

In this study, we performed a multicenter retrospective analysis to assess the correlation between total cholesterol (TC), high-density lipoprotein (HDL), low-density lipoprotein (LDL), TG, and primary frozen shoulder. The patients with already diagnosed diabetes mellitus and thyroid dysfunction were excluded. We then used two-sample Mendelian randomization (MR) to assess the causal relationship between primary frozen shoulder and dyslipidemia. This method avoids interference from reverse causal associations and potential confounders encountered in conventional randomized controlled trials by using the random distribution of genetic variation and simulating the randomization process of a randomized controlled experiment (10).

## 2 Materials and methods

### 2.1 Clinical study design

This retrospective observational study included patients with primary frozen shoulder diagnosed from October 2020 to October 2023 at four centers as the experimental group. The control group consisted of age- and sex-matched people who underwent a health checkup. The inclusion criteria were as follows:(a) Patients in the experimental group met the diagnostic criteria for primary frozen shoulder (11), including painful freezing phase, adhesive phase, and resolution phase; (b) The control population showed normal shoulder motion and no shoulder symptoms. The exclusion criteria were as follows: (a) Patients diagnosed with type 1 diabetes (12) and type 2 diabetes (13); (b) Patients diagnosed with hyperthyroidism (14) and hypothyroidism (15); (c) Patients with previous shoulder surgery or trauma.

The clinical data of all enrolled patients were collected, including gender, age, TC, TG, HDL, and LDL. The study was conducted under the Declaration of Helsinki and was approved by the ethics committees of each participating center. As the study was retrospective, the requirement for informed consent was waived.

### 2.2 Mendelian randomization study design

In this study, causal association analysis with outcome variables was performed by selecting Single nucleotide polymorphisms (SNPs) loci significantly associated with exposure factors as instrumental variables (IVs) and using the two-sample MR method. MR analysis needs to satisfy the following three core hypotheses: ① There was a strong correlation between IVs and exposure factors; ② IVs and any confounders associated with exposure-outcome were not relevant; ③ IVs affect outcomes only through exposure factors (16).

### 2.3 Sources of GWAS summary statistics

Summary-level GWAS data for hypothyroidism (17), hyperthyroidism (17), type 1 diabetes (18), type 2 diabetes (17), lipid traits (HDL, LDL, and TG) (19), and primary frozen shoulder (20) were sourced from published genome-wide association studies. All GWAS datasets utilized in this study were obtained from FinnGen and the UK Biobank and are publicly accessible via the IEU Open GWAS database summary website (https://gwas.mrcieu.ac.uk/). Summary information on the datasets used in this study is presented in **Table 1**. In addition, sex-stratified summary statistics for lipid traits and primary frozen shoulder were obtained from the UK Biobank (https://www.nealelab.is/uk-biobank) to analyze sex-specific genetic effects.

**Table 1:** Details of GWAS datasets used in the study.

Variable	ID	Sample size	SNPs	Population	Year
Hypothyroidism	ebi-a-GCST90018862	410,141	24,138,872	European	2021
Hyperthyroidism	ebi-a-GCST90018860	460,499	24,289,279	European	2021
Type 1 diabetes	ebi-a-GCST90014023	520,580	59,999,551	European	2021
Type 2 diabetes	ebi-a-GCST90018926	490,089	24,167,560	European	2021
HDL	ieu-b-109	403,943	12,321,875	European	2020
LDL	ieu-b-110	440,546	12,321,875	European	2020
TG	ieu-b-111	441,016	12,321,875	European	2020
Frozen shoulder	ebi-a-GCST90000512	451,099	15,184,371	European	2021

HDL, high-density lipoprotein; LDL, low-density lipoprotein; TG, triglycerides.

### 2.4 Selection of instrumental variables

SNPs in exposure factors were screened by setting a genome-wide significance threshold (*P *< 5. 0 × 10^− 8^). Second, independent SNPs without linkage disequilibrium (LD) were used as IVs according to the PLINK clumping algorithm (LD cut-off of r²< 0.001 within a 10 000-kilobase clumping window). The F statistic (F = beta^2^/se^2^) was used to assess the strength of genetic tools for all SNPs. We selected strong IVs with F-statistics above 10 for subsequent analysis. However, due to the large number of SNPs associated with lipid traits, we raised the F-statistic threshold to 100 to further optimize the quality of the instrumental variables and ensure the precision and robustness of the causal effect estimates. Additionally, SNPs associated with confounding factors (diabetes mellitus and thyroid dysfunction) were excluded when analyzing the causal associations between lipid traits and primary frozen shoulder.

### 2.5 Mendelian randomization analysis

In this study, inverse variance weighting (IVW) (21) was used as the primary method of analysis, with MR Egger (22), weighted median (23), simple mode, and weighted mode (24) as secondary methods of analysis.

Individual differences between IVs were tested using IVW and MR-Egger methods, and Cochran’s Q statistic and p-value were used to determine the presence of heterogeneity. To determine the possibility of horizontal pleiotropy, the MR-Egger intercept method calculates the intercept term from linear regression analysis. The study’s overall pleiotropy and horizontal pleiotropic outliers were evaluated using the MR pleiotropy residual sum and outlier (MR-PRESSO) test (25).

### 2.6 Statistical analysis

Statistical analysis was performed using SPSS 26.0 software. Continuous variables that satisfy normal distribution are expressed as mean ± standard deviation, and comparisons between groups are made using the independent samples t-test. Categorical variables were expressed as percentages, and comparisons between groups were made using the Chi-square test. The “TwoSampleMR” and “MR-PRESSO” packages in R (version 4.3.0) were used for MR analysis between exposures and results. The R script used in this analysis is provided in the **Supplementary Table 1**. The results are expressed as odds ratio (OR) and 95% confidence interval (CI) for each standard deviation. *P* < 0.05 was considered as statistical significance.

## 3 Results

### 3.1 No difference in serum lipids between primary frozen shoulder patients and normal subjects

The analysis showed that after excluding patients with diabetes mellitus and thyroid dysfunction, the serum lipids (TC, TG, HDL, and LDL) in the primary frozen shoulder group were no different from those of normal individuals (**Table 2**).

**Table 2:** Serum lipids levels in the study and control groups.

Characteristic	Frozen shoulder group (n = 284)	Control group (n = 284)	t/χ2	*P*
Male sex	125 (44%)	133(47%)	0.455	0.500
Age (years)	55.11 ± 5.94	55.25 ± 6.04	0.287	0.774
TC (mmol/L)	5.07 ± 0.72	4.99 ± 0.88	-1.321	0.187
TG (mmol/L)	1.19 ± 0.31	1.17 ± 0.32	-0.662	0.508
HDL (mmol/L)	1.26 ± 0.37	1.28 ± 0.41	0.627	0.531
LDL (mmol/L)	2.63 ± 0.70	2.54 ± 0.75	-1.509	0.132

HDL, high-density lipoprotein; LDL, low-density lipoprotein; TC, total cholesterol; TG, triglycerides.

### 3.2 Effect of diabetes mellitus and thyroid dysfunction on primary frozen shoulder

**Figure 1:**
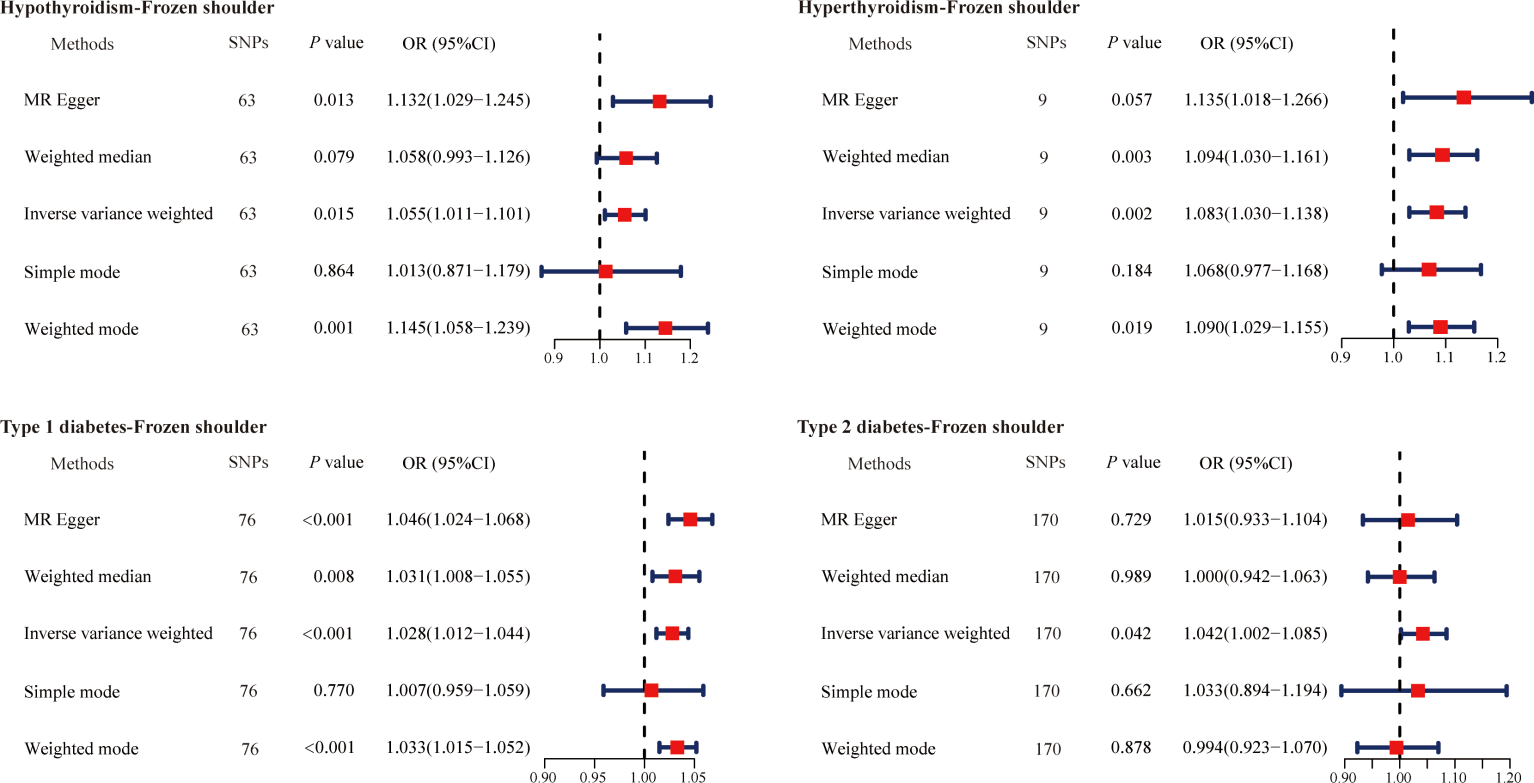
The causal effects of diabetes mellitus and thyroid dysfunction on primary frozen shoulder using two-sample Mendelian randomization methods. SNPs, single nucleotide polymorphisms; OR, odds ratios; CI, confidence interval.

We first evaluated the causal relationships between hypothyroidism, hyperthyroidism, type 1 diabetes mellitus, and type 2 diabetes mellitus with primary frozen shoulder by MR analysis. For subsequent analysis, 63 SNPs associated with hypothyroidism, 9 SNPs associated with hyperthyroidism, 76 SNPs associated with type 1 diabetes mellitus, and 170 SNPs associated with type 2 diabetes mellitus were identified (**Supplementary Tables 2**-**5**). The results revealed that the development of primary frozen shoulder was positively associated with hypothyroidism (OR = 1.055, 95% CI, 1.011−1.101, *P* = 0.015), hyperthyroidism (OR = 1.083, 95% CI, 1.030−1.138, *P* = 0.002), type 1 diabetes mellitus (OR = 1.028, 95% CI, 1.012−1.044, *P* < 0.001), and type 2 diabetes mellitus (OR = 1.042, 95% CI, 1.002−1.085, *P* = 0.042) (**Figure 1**). These findings provide additional evidence that hypothyroidism, hyperthyroidism, type 1 diabetes mellitus, and type 2 diabetes mellitus are independent risk factors for the development of primary frozen shoulder.

### 3.3 Effect of serum lipids on primary frozen shoulder

We first evaluated whether serum lipids have a causal effect on the onset of frozen shoulder without excluding potential confounders. The results indicated a positive association between LDL and the onset of primary frozen shoulder, while HDL and TG were negatively associated with its development (**Supplementary Figure 1**). However, significant heterogeneity and pleiotropy were observed in this analysis, which violates the core assumptions of MR analysis and indicates the influence of confounding factors (**Supplementary Figure 1**). In subsequent MR analyses of serum lipids and primary frozen shoulder, SNPs associated with these confounding factors will be excluded.

**Figure 2:**
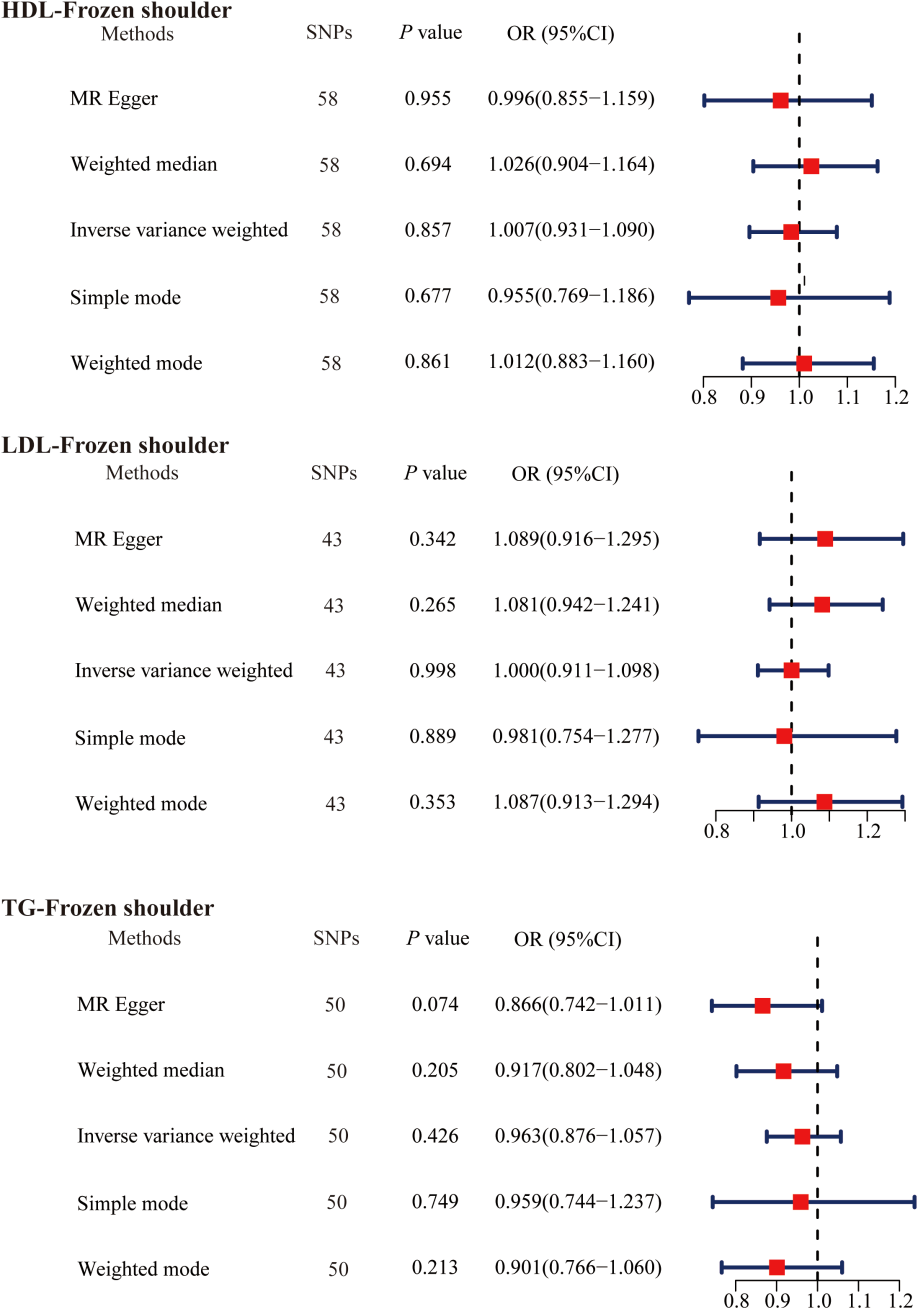
The causal effects of serum lipids on primary frozen shoulder using two-sample Mendelian randomization methods. SNPs, single nucleotide polymorphisms; OR, odds ratios; CI, confidence interval; HDL, high-density lipoprotein; LDL, low-density lipoprotein; TG, triglycerides.

A total of 151 SNPs (HDL 58, LDL 43, and TG 50) were identified for use in MR analysis by screening the acquired SNPs according to our study design criteria (**Supplementary Tables 6**-**8**). The main MR analysis results were as follows: HDL (OR = 1.007, 95% CI, 0.931−1.090, *P* = 0.857); LDL (OR = 1.000, 95% CI, 0.911−1.098, *P* = 0.998); TG (OR = 0.963, 95% CI, 0.876−1.057, *P* = 0.426) (**Figure 2**). No correlation was found between serum lipids and the risk of developing primary frozen shoulder by any of the MR analysis methods in this study.

**Figure 3:**
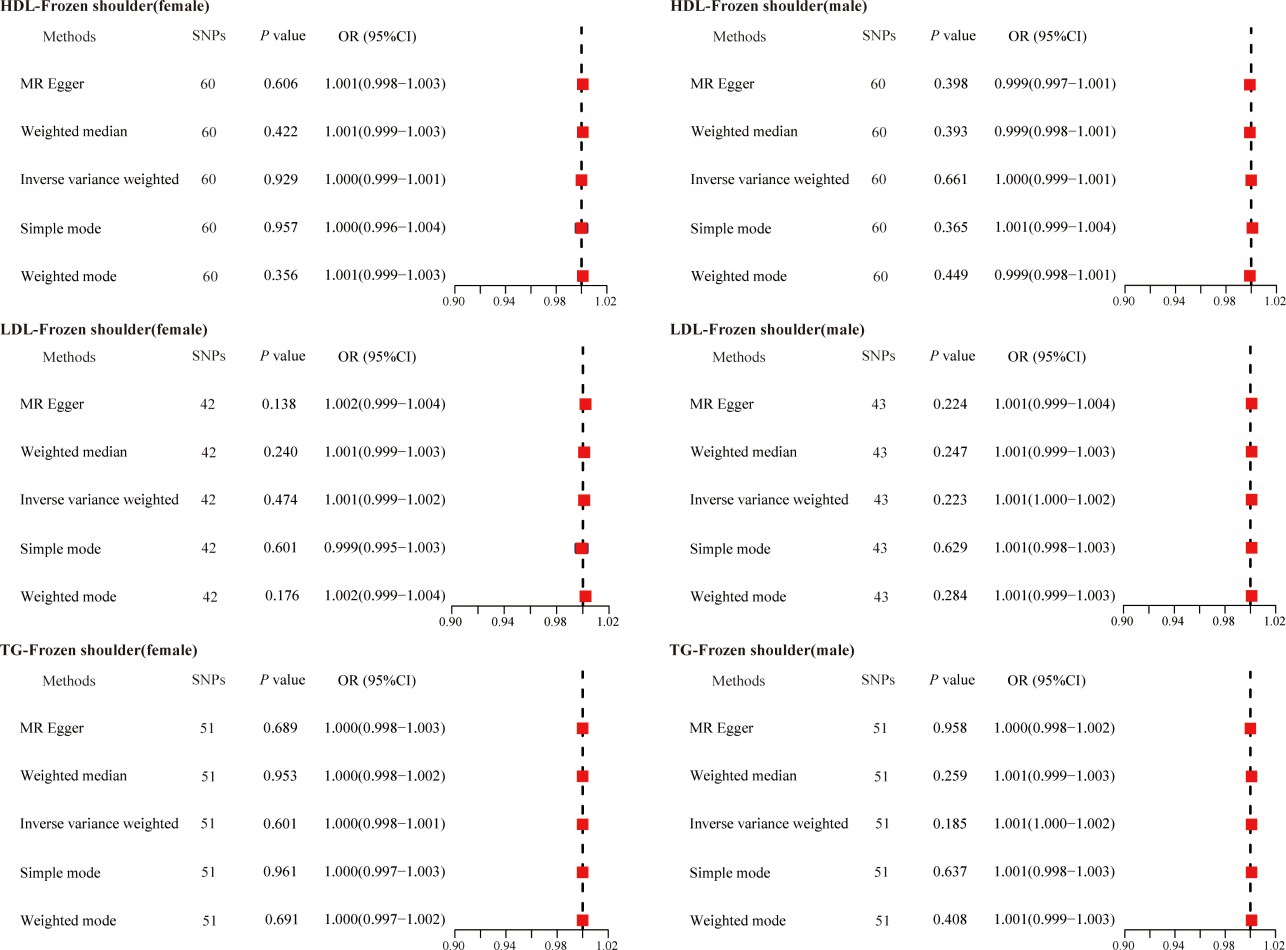
The causal effects of serum lipids on gender-stratified primary frozen shoulder using two-sample Mendelian randomization methods. SNPs, single nucleotide polymorphisms; OR, odds ratios; CI, confidence interval; HDL, high-density lipoprotein; LDL, low-density lipoprotein; TG, triglycerides.

Given that gender is an independent risk factor for primary frozen shoulder, we also evaluated the causal relationship between serum lipids and the gender-stratified onset of primary frozen shoulder. Specifically, we identified 60 SNPs (HDL-primary frozen shoulder in females), 60 SNPs (HDL-primary frozen shoulder in males), 42 SNPs (LDL-primary frozen shoulder in females), 43 SNPs (LDL-primary frozen shoulder in males), 51 SNPs (TG-primary frozen shoulder in females), and 51 SNPs (TG-primary frozen shoulder in males) as IVs (**Supplementary Tables 9**-**14**). However, none of the MR analysis methods revealed a causal association between serum lipids and the gender-specific onset of primary frozen shoulder (**Figure 3**).

### 3.4 Effect of primary frozen shoulder on serum lipids

**Figure 4:**
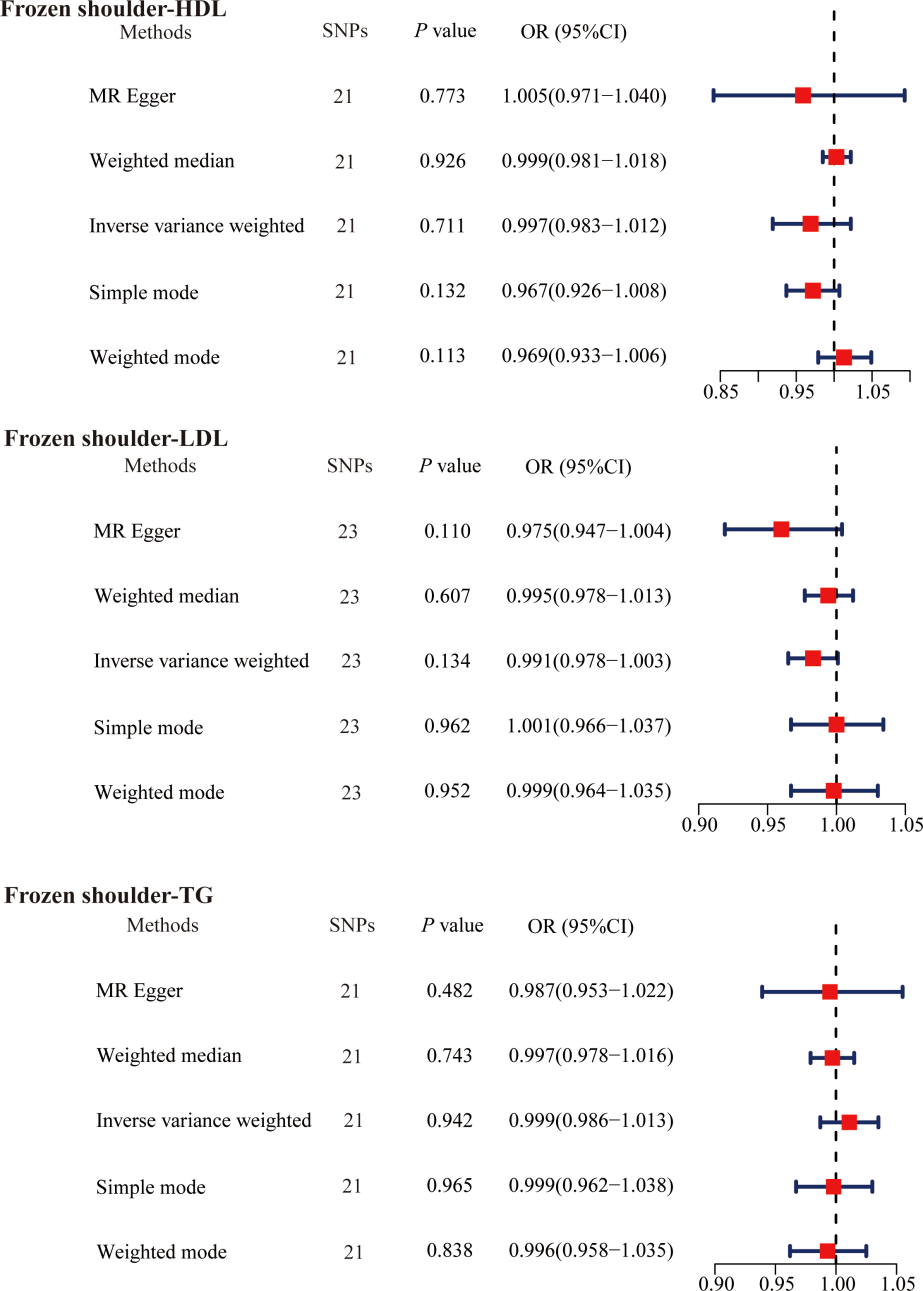
The causal effects of primary frozen shoulder on serum lipids using two-sample Mendelian randomization methods. SNPs, single nucleotide polymorphisms; OR, odds ratios; CI, confidence interval; HDL, high-density lipoprotein; LDL, low-density lipoprotein; TG, triglycerides.

We then explored whether the primary frozen shoulder contributes to abnormal serum lipid levels. We adjusted the threshold to *P* < 5. 0 × 10^− 6^ screening to obtain primary frozen shoulder -associated SNPs. Based on the design criteria, 21 SNPs (primary frozen shoulder-HDL), 23 SNPs (primary frozen shoulder-LDL), and 21 SNPs (primary frozen shoulder-TG) were finally obtained as IVs for MR analysis of the effect of primary frozen shoulder on serum lipids (**Supplementary Tables 15**-**17**). The results of the IVW method of primary frozen shoulder on serum lipids were analyzed as follows: primary frozen shoulder on HDL (OR = 0.997, 95% CI, 0.983−1.012, *P* = 0.711); primary frozen shoulder on LDL (OR = 0.991, 95% CI, 0.978−1.003, *P* = 0.134); primary frozen shoulder on TG (OR = 0.999, 95% CI, 0.986−1.013, *P* = 0.942) (**Figure 4**). All results did not find any effect of the primary frozen shoulder on serum lipids.

**Figure 5:**
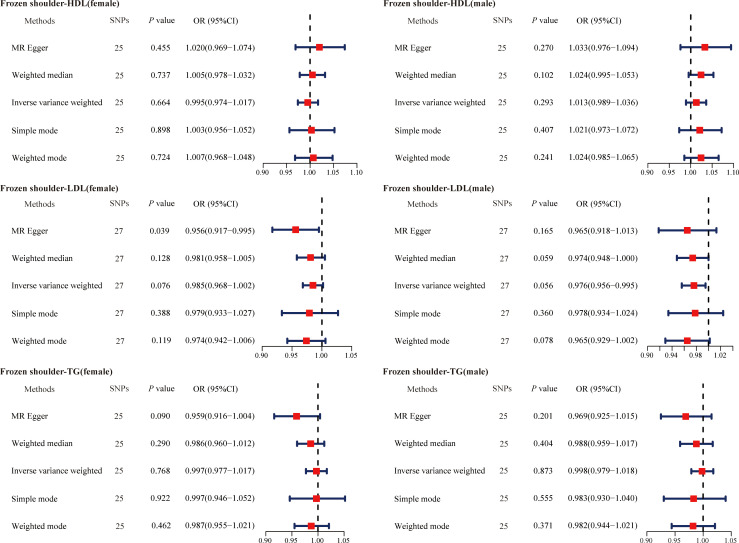
The causal effects of primary frozen shoulder on gender-stratified serum lipids using two-sample Mendelian randomization methods. SNPs, single nucleotide polymorphisms; OR, odds ratios; CI, confidence interval; HDL, high-density lipoprotein; LDL, low-density lipoprotein; TG, triglycerides.

The causal association between primary frozen shoulder and sex-stratified abnormal serum lipid levels was then analyzed in a similar manner. The IVs used for MR analysis consisted of 25 SNPs (primary frozen shoulder-HDL in females), 25 SNPs (primary frozen shoulder-HDL in males), 27 SNPs (primary frozen shoulder-LDL in females), 27 SNPs (primary frozen shoulder-LDL in males), 25 SNPs (primary frozen shoulder-TG in females), and 25 SNPs (primary frozen shoulder-TG in males) (**Supplementary Tables 18**-**23**). Consistent with these findings, no evidence was found to support a causal association between primary frozen shoulder and sex-specific changes in serum lipid levels (**Figure 5**).

### 3.5 Assessment of IVs validity and robustness in MR analysis

The Cochran Q test showed no intergenic heterogeneity among the IVs used for MR analysis in this study (**Table 3**). Moreover, neither the MR-Egger intercept test nor the MR-PRESSO global test showed signs of multidimensionality (**Table 4**). The funnel plot shows that the dispersion of causal association effects is largely symmetric and the results are not potentially biased (**Supplementary Figure 2**). In addition, “leave-one-out” sensitivity analyses showed that after eliminating each SNP in turn, the results of IVW analyses for the remaining SNPs were similar to the analyses that included all SNPs (**Supplementary Figure 3**).

**Table 3:** Heterogeneity test.

Exposure	Outcome	Heterogeneity test (MR-Egger)			Heterogeneity test (IVW)		
		Cochran's Q	Q_df	*P*	Cochran's Q	Q_df	*P*
Hypothyroidism	FS	77.067	61	0.080	80.361	62	0.058
Hyperthyroidism	FS	8.893	7	0.260	10.043	8	0.262
Type 1 diabetes	FS	81.404	74	0.260	87.440	75	0.154
Type 2 diabetes	FS	199.712	168	0.058	200.303	169	0.051
HDL	FS	56.262	56	0.465	56.293	57	0.502
HDL	FS-females	73.666	58	0.081	74.222	59	0.087
HDL	FS-males	64.084	58	0.272	64.67491	59	0.285
LDL	FS	38.404	41	0.587	39.709	42	0.572
LDL	FS-females	32.598	40	0.791	32.598	40	0.791
LDL	FS-males	34.403	41	0.757	34.403	41	0.757
TG	FS	52.311	48	0.310	55.373	49	0.247
TG	FS-females	46.677	49	0.568	47.474	50	0.575
TG	FS-males	58.081	49	0.176	59.406	50	0.170
FS	HDL	29.611	19	0.057	30.001	20	0.070
FS	HDL-females	30.741	23	0.129	32.165	24	0.123
FS	HDL-males	32.387	23	0.0925	33.221	24	0.100
FS	LDL	19.120	21	0.577	20.430	22	0.556
FS	LDL-females	25.418	25	0.439	27.921	26	0.362
FS	LDL-males	31.225	25	0.182	31.538	26	0.209
FS	TG	24.185	19	0.189	24.903	20	0.205
FS	TG-females	28.475	23	0.198	32.500	24	0.115
FS	TG-males	23.732	23	0.419	25.668	24	0.370

FS, frozen shoulder; HDL, high-density lipoprotein; LDL, low-density lipoprotein; TC, total cholesterol; TG, triglycerides.

**Table 4:** Pleiotropy test.

Exposure	Outcome	Horizontal pleiotropy test (MR-Egger)		MR-PRESSO
		Intercept	*P*	Global test *P*-value
Hypothyroidism	FS	-0.0081	0.112	0.051
Hyperthyroidism	FS	-0.0124	0.373	0.361
Type 1 diabetes	FS	-0.0066	0.122	0.155
Type 2 diabetes	FS	0.0022	0.482	0.050
HDL	FS	0.0007	0.862	0.500
HDL	FS-females	<-0.0001	0.511	0.107
HDL	FS-males	<-0.0001	0.467	0.315
LDL	FS	-0.0058	0.260	0.576
LDL	FS-females	<-0.0001	0.187	0.757
LDL	FS-males	<-0.0001	0.500	0.600
TG	FS	0.0075	0.100	0.267
TG	FS-females	<-0.0001	0.376	0.580
TG	FS-males	<-0.0001	0.296	0.209
FS	HDL	-0.0008	0.622	0.068
FS	HDL-females	-0.0027	0.313	0.148
FS	HDL-males	-0.0022	0.450	0.093
FS	LDL	0.0017	0.265	0.561
FS	LDL-females	0.0032	0.129	0.350
FS	LDL-males	0.0012	0.621	0.217
FS	TG	0.0013	0.462	0.215
FS	TG-females	0.0041	0.084	0.117
FS	TG-males	0.0032	0.184	0.389

FS, frozen shoulder; HDL, high-density lipoprotein; LDL, low-density lipoprotein; TC, total cholesterol; TG, triglycerides.

## 4 Discussion

Previous clinical studies have shown that dyslipidemia and primary frozen shoulder are closely related, and may be a risk factor for the development of primary frozen shoulder (9). In the present study, we obtained different results for the first time. After excluding patients with diabetes mellitus and thyroid dysfunction, there was no difference in serum lipid levels between the primary frozen shoulder group and the normal control group. In addition, the results of MR analyses also indicated that there was no causal relationship between dyslipidemia and primary frozen shoulder.

An analysis of data based on the National Health Insurance Research Database of Taiwan found that patients with hyperlipidemia had a 1.5-fold higher risk of adhesive capsulitis than did healthy controls (26). Several potential mechanisms may explain how blood lipids could influence primary frozen shoulder. In individuals with dyslipidemia, inflammatory factors such as interleukins and tumor necrosis factor-alpha are often elevated, which may promote the development of primary frozen shoulder through inflammatory pathways (27, 28). Additionally, alterations in lipid metabolism could affect the function of synovial tissue in the shoulder joint. Lipids influence the integrity of cell membranes and signaling pathways, including those associated with peroxisome proliferator-activated receptors and the NF-kB pathway, both of which play crucial roles in inflammation and fibrosis (29). Of note, the study also found that the use of statins did not prevent frozen shoulder in patients with hyperlipidemia (26). This result is thought-provoking. Diabetes mellitus and thyroid dysfunction are independent risk factors for the development of primary frozen shoulder (4). And dyslipidemia is an important component of diabetic metabolic syndrome and thyroid dysfunction (7, 8). We therefore hypothesized that serum lipid levels in patients with primary frozen shoulder may be affected by diabetes mellitus and thyroid dysfunction. This was confirmed in the multicenter retrospective analysis conducted in this study.

In addition, although some single-center, small-sample studies have demonstrated a relationship between dyslipidemia and frozen shoulder. However, no study has been able to confirm whether dyslipidemia is a cause, an associated cofactor, or a consequence of primary frozen shoulder. In this study, the causal genetic link between frozen shoulder and serum lipids was examined using a two-sample MR approach based on a sizable GWAS database. To the best of our knowledge, this is the first study to use MR methods to assess the causal relationship between serum lipids and frozen shoulder. Unfortunately, the results of the MR analysis did not reveal a direct link between frozen shoulder and serum lipids.

The above evidence indicates that diabetes mellitus and thyroid dysfunction interfere with the relationship between dyslipidemia and the risk of developing primary frozen shoulder to the extent that the association is overestimated. This further explains why the use of statins does not protect hyperlipidemic patients from primary frozen shoulder. Although serum lipids improve with statins, diabetes mellitus and thyroid dysfunction may persist. This observation emphasizes the need for a more nuanced understanding of risk factors for primary frozen shoulder in clinical practice. For instance, clinicians should focus on managing systemic conditions like diabetes and thyroid dysfunction, which may indirectly influence the development of frozen shoulder. This shift in focus could lead to more targeted and effective preventive strategies, reducing unnecessary interventions aimed solely at lipid management in such patients. At the same time, we believe that there may be other potential factors contributing to the relationship between lipids and primary frozen shoulder that we have not yet focused on or observed. Larger genetic and intervention studies are needed to further investigate the pathophysiology of serum lipids and primary frozen shoulder.

The greatest strength of our study was the combination of retrospective and MR analyses, which reduced the effects of reverse causality and confounding factors. Secondly, we performed a gender-stratified MR analysis to identify gender-specific causal associations. Finally, we used multiple MR analysis methods, which increased the credibility and authenticity of our findings. In clinical settings, similar MR analyses could be applied to identify and validate causal risk factors for other musculoskeletal or metabolic disorders. This evidence-based approach could guide the development of precision medicine strategies, ensuring that interventions are directed at modifiable risk factors with proven causal links to disease outcomes.

Inevitably, the current study has some limitations. First, we may not be able to exclude all confounders and eliminate biased estimates of causal inference. Confounding factors such as subclinical disease states, different stages of disease progression, diet, and lifestyle may influence the relationship between serum lipids and primary frozen shoulder. Second, the GWAS dataset we use is primarily from European-origin populations. The population included in previous clinical studies and our retrospective analysis consisted of Asian individuals. The emergence of specific traits varies across racial and ethnic groups due to their different living environments and genetic backgrounds. This therefore limits the application of our findings to other populations of different races. Finally, the sample size included in this study was too small because serum lipids are not a routine examination for patients with primary frozen shoulder.

## 5 Conclusion

In conclusion, retrospective observational studies and MR analyses did not reveal correlations and causal genetic relationships between serum lipids and primary frozen shoulder. These findings deepen our understanding of risk factors for primary frozen shoulder. Further studies on the pathophysiologic relationship between serum lipids and primary frozen shoulder are warranted.

## Data Availability

The original contributions presented in the study are included in the article/**Supplementary Material**. Further inquiries can be directed to the corresponding author.
