# Supplementary material for: The association between primary frozen shoulder and serum lipids may be overestimated: evidence based on retrospective observational studies and Mendelian randomization
Source: Front Endocrinol (Lausanne). 2025 Jan 15;15:1363018. doi: 10.3389/fendo.2024.1363018 (PMC11774719; doi:10.3389/fendo.2024.1363018)

**Supplementary materials**

Supplement table 1: The R script used to perform the MR analyses in this study.

Supplement table 2: Summary data of SNPs used in MR analysis of IVs associated with hypothyroidism-frozen shoulder.

Supplement table 3: Summary data of SNPs used in MR analysis of IVs associated with hyperthyroidism-frozen shoulder.

Supplement table 4: Summary data of SNPs used in MR analysis of IVs associated with type 1 diabetes-frozen shoulder.

Supplement table 5: Summary data of SNPs used in MR analysis of IVs associated with type 2 diabetes-frozen shoulder.

Supplement table 6: Summary data of SNPs used in MR analysis of IVs associated with HDL- frozen shoulder.

Supplement table 7: Summary data of SNPs used in MR analysis of IVs associated with LDL- frozen shoulder.

Supplement table 8: Summary data of SNPs used in MR analysis of IVs associated with TG- frozen shoulder.

Supplement table 9: Summary data of SNPs used in MR analysis of IVs associated with HDL- frozen shoulder in females.

Supplement table 10: Summary data of SNPs used in MR analysis of IVs associated with HDL- frozen shoulder in males.

Supplement table 11: Summary data of SNPs used in MR analysis of IVs associated with LDL- frozen shoulder in females.

Supplement table 12: Summary data of SNPs used in MR analysis of IVs associated with LDL- frozen shoulder in males.

Supplement table 13: Summary data of SNPs used in MR analysis of IVs associated with TG- frozen shoulder in females.

Supplement table 14: Summary data of SNPs used in MR analysis of IVs associated with TG- frozen shoulder in males.

Supplement table 15: Summary data of SNPs used in MR analysis of IVs associated with frozen shoulder-HDL.

Supplement table 16: Summary data of SNPs used in MR analysis of IVs associated with frozen shoulder-LDL.

Supplement table 17: Summary data of SNPs used in MR analysis of IVs associated with frozen shoulder-TG.

Supplement table 18: Summary data of SNPs used in MR analysis of IVs associated with frozen shoulder-HDL in females.

Supplement table 19: Summary data of SNPs used in MR analysis of IVs associated with frozen shoulder-HDL in males.

Supplement table 20: Summary data of SNPs used in MR analysis of IVs associated with frozen shoulder-LDL in females.

Supplement table 21: Summary data of SNPs used in MR analysis of IVs associated with frozen shoulder-LDL in males.

Supplement table 22: Summary data of SNPs used in MR analysis of IVs associated with frozen shoulder-TG in females.

Supplement table 23: Summary data of SNPs used in MR analysis of IVs associated with frozen shoulder-TG in males.

Supplement figure 1: MR analysis of serum lipids and frozen shoulder without adjusting for confounders.

Supplement figure 2: Funnel plots of the causal effects.

Supplement figure 3: Leave-one-out sensitivity analysis.

Supplement table 1. The R script used to perform the MR analyses in this study.

library(VariantAnnotation)

library(gwasglue)

library(TwoSampleMR)

exposureName="exposure"

outcomeName="outcome"

exposureFile="exposure.csv"

outcomeFile="outcome.csv"

setwd("C:\\Mendelian")

exposure_dat<-read_exposure_data(filename=exposureFile,

sep = ",",

snp_col = "SNP",

beta_col = "beta.exposure",

se_col = "se.exposure",

effect_allele_col = "effect_allele.exposure",

other_allele_col = "other_allele.exposure",

eaf_col = "eaf.exposure",

clump = F)

outcome_dat<-read_outcome_data(snps=exposure_dat$SNP,

filename="outcome.csv", sep = ",",

snp_col = "SNP",

beta_col = "beta.outcome",

se_col = "se.outcome",

effect_allele_col = "effect_allele.outcome",

other_allele_col = "other_allele.outcome",

pval_col = "pval.outcome",

eaf_col = "eaf.outcome")

exposure_dat$exposure=exposureName

outcome_dat$outcome=outcomeName

dat<-harmonise_data(exposure_dat=exposure_dat,

outcome_dat=outcome_dat)

outTab=dat[dat$mr_keep=="TRUE",]

write.csv(outTab, file="table.SNP.csv", row.names=F)

presso=run_mr_presso(dat)

write.csv(presso[[1]]$`MR-PRESSO results`$`Outlier Test`, file="table.MR-PRESSO.csv")

mrResult=mr(dat)

#mr_method_list()$obj

#mrResult=mr(dat, method_list=c("mr_ivw", "mr_egger_regression", "mr_weighted_median", "mr_simple_mode", "mr_weighted_mode"))

mrTab=generate_odds_ratios(mrResult)

write.csv(mrTab, file="table.MRresult.csv", row.names=F)

heterTab=mr_heterogeneity(dat)

write.csv(heterTab, file="table.heterogeneity.csv", row.names=F)

pleioTab=mr_pleiotropy_test(dat)

write.csv(pleioTab, file="table.pleiotropy.csv", row.names=F)

pdf(file="pic.scatter_plot.pdf", width=7.5, height=7)

mr_scatter_plot(mrResult, dat)

dev.off()

res_single=mr_singlesnp(dat)

pdf(file="pic.forest.pdf", width=7, height=6.5)

mr_forest_plot(res_single)

dev.off()

pdf(file="pic.funnel_plot.pdf", width=7, height=6.5)

mr_funnel_plot(singlesnp_results = res_single)

dev.off()

pdf(file="pic.leaveoneout.pdf", width=7, height=6.5)

mr_leaveoneout_plot(leaveoneout_results = mr_leaveoneout(dat))

dev.off()

presso[[1]]$`MR-PRESSO results`$`Global Test`

Supplement table 2. Summary data of SNPs used in MR analysis of IVs associated with hypothyroidism-frozen shoulder.

| **SNP** | **Effect Allele** | **Other Allele** | **EAF** | **Beta Estimate** | **SE** | **F-statistic** | **P value** | **beta.FS** | **se.FS** | **eaf.FS** | **Pval.FS** |
| --- | --- | --- | --- | --- | --- | --- | --- | --- | --- | --- | --- |
| rs10075764 | G | A | 0.302413 | -0.057 | 0.0104 | 562.9960602 | 4.23E-08 | 0.0325978 | 0.0146584 | 0.341257 | 0.025 |
| rs10126000 | A | C | 0.688692 | -0.0683 | 0.0104 | 822.029424 | 5.12E-11 | -0.00300343 | 0.0143882 | 0.627625 | 0.84 |
| rs10424978 | A | C | 0.624328 | -0.0775 | 0.0102 | 1158.807868 | 3.01E-14 | -0.0042782 | 0.0143293 | 0.59985 | 0.77 |
| rs10494077 | A | G | 0.249595 | 0.1284 | 0.0118 | 2548.667224 | 1.41E-27 | -0.0177357 | 0.0165769 | 0.228565 | 0.29 |
| rs10748781 | A | C | 0.623331 | -0.0592 | 0.0102 | 676.080472 | 6.48E-09 | 0.00108414 | 0.0141039 | 0.566125 | 0.92 |
| rs1079418 | G | A | 0.262374 | -0.0657 | 0.011 | 686.396453 | 2.33E-09 | 0.0207751 | 0.0150876 | 0.305506 | 0.17 |
| rs11171710 | A | G | 0.443916 | -0.0698 | 0.01 | 988.9150919 | 2.95E-12 | 0.012771 | 0.0140606 | 0.447866 | 0.38 |
| rs11675342 | T | C | 0.387729 | 0.0906 | 0.01 | 1604.668381 | 1.30E-19 | 0.0150287 | 0.0140681 | 0.422834 | 0.28 |
| rs11875260 | G | A | 0.172925 | 0.0751 | 0.0135 | 662.7425046 | 2.65E-08 | 0.0066219 | 0.0196315 | 0.148133 | 0.75 |
| rs12117927 | A | C | 0.476541 | 0.0627 | 0.0105 | 805.993825 | 2.35E-09 | 0.0113352 | 0.0142481 | 0.488874 | 0.42 |
| rs12379417 | A | G | 0.317559 | 0.0583 | 0.0103 | 605.1011476 | 1.51E-08 | 0.0216408 | 0.0144164 | 0.367495 | 0.13 |
| rs12582330 | T | G | 0.624365 | -0.061 | 0.0109 | 717.1070852 | 2.19E-08 | -0.0161201 | 0.0156613 | 0.728577 | 0.32 |
| rs12593201 | A | G | 0.325432 | 0.0905 | 0.0112 | 1480.160216 | 6.46E-16 | -0.00833468 | 0.0160371 | 0.250327 | 0.6 |
| rs12984428 | A | G | 0.35604 | -0.0659 | 0.0102 | 818.3805392 | 1.04E-10 | -0.0155529 | 0.014148 | 0.407326 | 0.3 |
| rs13090803 | T | G | 0.190624 | 0.0829 | 0.0128 | 871.6055478 | 9.38E-11 | 0.0118337 | 0.0171033 | 0.21238 | 0.48 |
| rs13109179 | A | G | 0.461895 | 0.0647 | 0.01 | 855.2332433 | 9.80E-11 | -0.000439368 | 0.0141219 | 0.411403 | 0.99 |
| rs1364450 | C | A | 0.126235 | 0.0886 | 0.0139 | 711.4684641 | 1.84E-10 | -0.00657603 | 0.0197331 | 0.145496 | 0.73 |
| rs142997491 | G | A | 0.0135637 | 0.2385 | 0.0412 | 625.2397941 | 7.09E-09 | -0.031886 | 0.0639072 | 0.012199 | 0.63 |
| rs1432806 | G | A | 0.338619 | 0.0583 | 0.0105 | 625.3496145 | 2.82E-08 | 0.0201109 | 0.0146151 | 0.348034 | 0.17 |
| rs1479565 | A | G | 0.498866 | 0.0975 | 0.0101 | 1958.742004 | 4.75E-22 | -0.0267636 | 0.0142988 | 0.381603 | 0.058 |
| rs1534430 | T | C | 0.428394 | -0.086 | 0.0101 | 1490.987706 | 1.67E-17 | -0.0109844 | 0.0142552 | 0.390338 | 0.42 |
| rs2111485 | G | A | 0.479023 | 0.0813 | 0.0102 | 1357.53861 | 1.58E-15 | -0.00375401 | 0.0142131 | 0.608778 | 0.8 |
| rs2114702 | A | T | 0.26671 | 0.07 | 0.0111 | 787.5994275 | 2.86E-10 | 0.00968998 | 0.0149974 | 0.311979 | 0.52 |
| rs2247314 | C | T | 0.376495 | -0.086 | 0.0104 | 1429.116967 | 1.35E-16 | -0.0140424 | 0.014717 | 0.335648 | 0.33 |
| rs229528 | T | C | 0.496016 | 0.0903 | 0.01 | 1678.893485 | 1.72E-19 | -0.0111685 | 0.0140659 | 0.42355 | 0.41 |
| rs2445608 | A | G | 0.430366 | -0.0593 | 0.0101 | 708.3595274 | 4.32E-09 | -0.00145133 | 0.0141174 | 0.409513 | 0.91 |
| rs244685 | G | T | 0.79915 | -0.0858 | 0.0132 | 971.5459121 | 8.03E-11 | 0.0306605 | 0.0210862 | 0.876142 | 0.15 |
| rs2476601 | G | A | 0.891439 | -0.3663 | 0.0159 | 10935.23102 | 1.95E-117 | -0.0580848 | 0.0229145 | 0.898197 | 0.011 |
| rs2988277 | T | C | 0.287022 | 0.0593 | 0.0106 | 591.135807 | 2.21E-08 | 0.0154829 | 0.0141825 | 0.398361 | 0.28 |
| rs307558 | A | G | 0.742625 | -0.0688 | 0.0119 | 743.4647883 | 7.40E-09 | 0.0238087 | 0.0172518 | 0.796343 | 0.17 |
| rs3087243 | A | G | 0.386088 | -0.1466 | 0.0102 | 4221.518827 | 7.69E-47 | -0.0228927 | 0.0139505 | 0.449862 | 0.1 |
| rs3118469 | T | A | 0.295174 | 0.0803 | 0.0106 | 1103.364357 | 3.58E-14 | 0.0133171 | 0.0151291 | 0.301183 | 0.37 |
| rs3184504 | C | T | 0.668513 | -0.1734 | 0.0102 | 5539.39058 | 8.21E-65 | -0.00934912 | 0.0138805 | 0.517363 | 0.53 |
| rs34536443 | C | G | 0.0440279 | -0.1863 | 0.0263 | 1201.79579 | 1.40E-12 | 0.00518692 | 0.0342253 | 0.045704 | 0.88 |
| rs3775291 | T | C | 0.287788 | -0.0649 | 0.0108 | 709.3865384 | 1.86E-09 | 0.0120478 | 0.0151706 | 0.297655 | 0.44 |
| rs434294 | G | A | 0.322802 | -0.0683 | 0.0109 | 838.1871569 | 3.70E-10 | -0.004688 | 0.014841 | 0.323255 | 0.75 |
| rs4409785 | C | T | 0.142566 | 0.1069 | 0.0133 | 1149.074504 | 9.16E-16 | -0.00280444 | 0.0183885 | 0.172284 | 0.88 |
| rs4529854 | T | C | 0.723438 | -0.0768 | 0.0107 | 970.2944688 | 7.10E-13 | -0.0274888 | 0.0145333 | 0.644806 | 0.055 |
| rs4835534 | C | T | 0.156356 | -0.1421 | 0.0132 | 2196.558193 | 5.03E-27 | 0.00840757 | 0.0169792 | 0.212158 | 0.61 |
| rs61759532 | T | C | 0.188941 | 0.0905 | 0.0122 | 1032.115761 | 1.19E-13 | -0.03656 | 0.0165296 | 0.247778 | 0.03 |
| rs61877856 | T | C | 0.197495 | -0.0658 | 0.0115 | 563.6547159 | 1.05E-08 | 0.0173563 | 0.0154925 | 0.278517 | 0.27 |
| rs6908626 | T | G | 0.17075 | 0.1441 | 0.0141 | 2426.032226 | 1.62E-24 | 0.0157027 | 0.0182164 | 0.179081 | 0.37 |
| rs7030280 | T | C | 0.745664 | 0.2075 | 0.0108 | 6809.251381 | 2.88E-82 | -0.00062634 | 0.014768 | 0.667558 | 0.96 |
| rs71508903 | T | C | 0.210816 | 0.0934 | 0.0125 | 1193.985703 | 7.90E-14 | 0.0331635 | 0.0177378 | 0.193477 | 0.06 |
| rs7223956 | C | T | 0.897005 | -0.0902 | 0.0144 | 617.5022203 | 3.75E-10 | -0.0163876 | 0.0198018 | 0.855621 | 0.43 |
| rs73192661 | T | C | 0.428858 | -0.1061 | 0.01 | 2274.317317 | 2.68E-26 | 0.0219443 | 0.0139906 | 0.451564 | 0.12 |
| rs736374 | A | G | 0.375103 | 0.0832 | 0.0103 | 1335.298432 | 6.60E-16 | -0.0146692 | 0.0144791 | 0.363212 | 0.33 |
| rs7441808 | G | A | 0.21091 | 0.0766 | 0.0111 | 802.5855542 | 5.17E-12 | -0.00158207 | 0.0151197 | 0.301702 | 0.93 |
| rs7574865 | G | T | 0.74252 | -0.1321 | 0.0117 | 2755.027181 | 1.46E-29 | 0.000473343 | 0.0166507 | 0.776524 | 0.95 |
| rs76518703 | G | A | 0.0723811 | -0.1554 | 0.0236 | 1334.34613 | 4.56E-11 | -0.0442867 | 0.0326858 | 0.052161 | 0.18 |
| rs7742626 | C | T | 0.267343 | 0.0686 | 0.0116 | 757.495723 | 3.34E-09 | 0.018896 | 0.0155574 | 0.27499 | 0.24 |
| rs7767978 | T | C | 0.308123 | -0.1291 | 0.0115 | 2935.376054 | 3.04E-29 | -0.0252246 | 0.016135 | 0.244235 | 0.12 |
| rs79490353 | C | T | 0.0241151 | 0.2006 | 0.0349 | 778.276504 | 9.04E-09 | -0.00334276 | 0.0440623 | 0.025422 | 0.94 |
| rs7990020 | C | A | 0.453482 | 0.0577 | 0.0101 | 677.9450505 | 1.11E-08 | -0.00645357 | 0.0139196 | 0.541233 | 0.65 |
| rs853305 | C | T | 0.718894 | -0.0802 | 0.0111 | 1068.99397 | 5.00E-13 | -0.0203939 | 0.0149272 | 0.68465 | 0.17 |
| rs881858 | A | G | 0.747899 | 0.0665 | 0.0108 | 685.0885475 | 7.39E-10 | 0.0210304 | 0.0150288 | 0.693781 | 0.17 |
| rs926103 | C | T | 0.697985 | -0.0678 | 0.0104 | 796.4115571 | 7.07E-11 | -0.0235865 | 0.014675 | 0.651938 | 0.11 |
| rs9271365 | G | T | 0.443466 | 0.2484 | 0.0105 | 12883.95329 | 9.97E-124 | 0.0453736 | 0.0140439 | 0.429177 | 0.0014 |
| rs9277569 | T | C | 0.16088 | 0.1369 | 0.0149 | 2085.918147 | 4.01E-20 | 0.000619141 | 0.0223801 | 0.108043 | 0.98 |
| rs9497965 | T | C | 0.409035 | 0.0827 | 0.0102 | 1360.606906 | 5.15E-16 | -0.0182707 | 0.014154 | 0.409343 | 0.19 |
| rs9511151 | A | G | 0.279567 | -0.0976 | 0.0106 | 1579.828205 | 3.34E-20 | 0.00327976 | 0.0146439 | 0.346256 | 0.8 |
| rs9625921 | G | C | 0.306819 | -0.0654 | 0.0103 | 747.5432267 | 2.16E-10 | 0.0132863 | 0.0144635 | 0.359654 | 0.38 |
| rs9902341 | T | C | 0.172257 | 0.0801 | 0.0129 | 751.7852934 | 5.32E-10 | 0.0348353 | 0.0186679 | 0.165375 | 0.062 |

Abbreviations: SE, standard error; EAF, effect allele frequency; SNP, single nucleotide polymorphism; FS, frozen shoulder.

Supplement table 3. Summary data of SNPs used in MR analysis of IVs associated with hyperthyroidism-frozen shoulder.

| **SNP** | **Effect Allele** | **Other Allele** | **EAF** | **Beta Estimate** | **SE** | **F-statistic** | **P value** | **beta.FS** | **se.FS** | **eaf.FS** | **Pval.FS** |
| --- | --- | --- | --- | --- | --- | --- | --- | --- | --- | --- | --- |
| rs1126511 | T | G | 0.198475 | -0.1903 | 0.0274 | 5367.725311 | 3.78E-12 | -0.00543939 | 0.016828 | 0.217451 | 0.74 |
| rs1794280 | T | A | 0.098516 | 0.5463 | 0.0391 | 25777.31854 | 2.31E-44 | 0.0515534 | 0.019582 | 0.147448 | 0.0085 |
| rs2160215 | C | T | 0.425594 | 0.2483 | 0.0226 | 14312.56114 | 4.43E-28 | 0.00790682 | 0.014374 | 0.375466 | 0.57 |
| rs2476601 | G | A | 0.887841 | -0.2961 | 0.0378 | 8183.76722 | 4.75E-15 | -0.0580848 | 0.022915 | 0.898197 | 0.011 |
| rs3087243 | A | G | 0.380249 | -0.2038 | 0.0224 | 9194.678269 | 9.18E-20 | -0.0228927 | 0.013951 | 0.449862 | 0.1 |
| rs4338740 | C | T | 0.207075 | 0.1844 | 0.0264 | 5200.141753 | 2.85E-12 | 0.0146715 | 0.015818 | 0.260367 | 0.35 |
| rs58722186 | T | C | 0.302235 | 0.1359 | 0.0233 | 3615.313286 | 5.46E-09 | -0.00242993 | 0.014989 | 0.312379 | 0.9 |
| rs604912 | G | A | 0.538777 | 0.1198 | 0.0217 | 3308.25748 | 3.38E-08 | 0.0241658 | 0.014245 | 0.613269 | 0.086 |
| rs6131010 | G | A | 0.712807 | 0.1306 | 0.0237 | 3238.414378 | 3.58E-08 | -0.0233575 | 0.015864 | 0.739796 | 0.15 |

Abbreviations: SE, standard error; EAF, effect allele frequency; SNP, single nucleotide polymorphism; FS, frozen shoulder.

Supplement table 4. Summary data of SNPs used in MR analysis of IVs associated with type 1 diabetes-frozen shoulder.

| **SNP** | **Effect Allele** | **Other Allele** | **EAF** | **Beta Estimate** | **SE** | **F-statistic** | **P value** | **beta.FS** | **se.FS** | **eaf.FS** | **Pval.FS** |
| --- | --- | --- | --- | --- | --- | --- | --- | --- | --- | --- | --- |
| rs10224046 | G | T | 0.324 | 0.085811 | 0.015436 | 1684.599058 | 2.71E-08 | 0.00888625 | 0.015038 | 0.310485 | 0.56 |
| rs10484565 | A | G | 0.0866 | 0.6635 | 0.023923 | 38969.77733 | 2.66E-169 | -0.00447358 | 0.0261977 | 0.075713 | 0.88 |
| rs1050979 | G | A | 0.515 | 0.106196 | 0.014129 | 2949.406976 | 5.64E-14 | 0.0180805 | 0.0138799 | 0.528948 | 0.2 |
| rs10801128 | G | A | 0.717 | 0.096073 | 0.015681 | 1957.284303 | 8.97E-10 | -0.00813839 | 0.0153004 | 0.710192 | 0.6 |
| rs11203203 | A | G | 0.349 | 0.143803 | 0.014405 | 4938.0822 | 1.81E-23 | -0.00113896 | 0.0143968 | 0.36671 | 0.94 |
| rs113374757 | T | C | 0.163 | -0.171277 | 0.020769 | 4200.657932 | 1.63E-16 | -0.0117745 | 0.0188409 | 0.163974 | 0.54 |
| rs114378220 | T | C | 0.0703 | 0.177902 | 0.030444 | 2162.59923 | 5.11E-09 | 0.0700099 | 0.0276378 | 0.073755 | 0.011 |
| rs12128789 | C | T | 0.132 | 0.126969 | 0.021535 | 1930.243938 | 3.73E-09 | -0.0173001 | 0.020202 | 0.13737 | 0.4 |
| rs12257077 | T | C | 0.0319 | 0.231245 | 0.036955 | 1725.075807 | 3.91E-10 | -0.058949 | 0.0372583 | 0.036009 | 0.11 |
| rs12464462 | G | A | 0.41 | -0.087955 | 0.014341 | 1955.695475 | 8.62E-10 | -0.000970025 | 0.0141077 | 0.416463 | 0.94 |
| rs12644686 | G | C | 0.194 | -0.10775 | 0.01932 | 1896.997847 | 2.45E-08 | 0.0113872 | 0.0182816 | 0.178004 | 0.56 |
| rs12927355 | T | C | 0.316 | -0.203881 | 0.015188 | 9525.490629 | 4.39E-41 | 0.0103694 | 0.0148728 | 0.321675 | 0.49 |
| rs13018977 | A | T | 0.225 | 0.100335 | 0.017882 | 1834.139596 | 2.01E-08 | -0.00925279 | 0.0166445 | 0.224842 | 0.54 |
| rs13147049 | G | A | 0.641 | -0.109522 | 0.014689 | 2889.849453 | 8.92E-14 | 0.00677969 | 0.0147498 | 0.669777 | 0.66 |
| rs13259300 | C | A | 0.598 | -0.092191 | 0.014669 | 2135.986015 | 3.28E-10 | 0.0078685 | 0.0143535 | 0.608368 | 0.56 |
| rs1350275 | G | T | 0.698 | -0.093665 | 0.015283 | 1932.600742 | 8.86E-10 | -0.0016207 | 0.0155531 | 0.722884 | 0.91 |
| rs144706316 | C | T | 0.00422 | 0.578307 | 0.101952 | 1467.340715 | 1.41E-08 | 0.176508 | 0.100057 | 0.004886 | 0.078 |
| rs150853727 | A | G | 0.0363 | -0.3337 | 0.038541 | 4087.648311 | 4.79E-18 | 0.00130995 | 0.0350712 | 0.040721 | 0.95 |
| rs1574285 | T | G | 0.591 | -0.126548 | 0.014171 | 4061.743963 | 4.26E-19 | -0.00133404 | 0.0142012 | 0.600179 | 0.93 |
| rs1701704 | G | T | 0.339 | 0.244048 | 0.014558 | 14276.35016 | 4.49E-63 | 0.00983629 | 0.0146133 | 0.342254 | 0.5 |
| rs17106304 | G | C | 0.656 | 0.115351 | 0.014812 | 3145.116586 | 6.83E-15 | -0.0176436 | 0.0146192 | 0.655952 | 0.22 |
| rs17323934 | G | C | 0.223 | -0.129663 | 0.016818 | 3050.784427 | 1.26E-14 | -0.00169613 | 0.0163932 | 0.234171 | 0.93 |
| rs17623914 | C | T | 0.0997 | -0.134887 | 0.023381 | 1705.922324 | 7.97E-09 | -0.00343921 | 0.0225605 | 0.108604 | 0.87 |
| rs1808094 | C | T | 0.524 | -0.113651 | 0.0149 | 3376.042879 | 2.39E-14 | 0.00418555 | 0.0139165 | 0.525512 | 0.73 |
| rs1881146 | T | A | 0.311 | -0.09517 | 0.017407 | 2028.544734 | 4.57E-08 | -0.00833311 | 0.0150691 | 0.31284 | 0.55 |
| rs1947178 | G | A | 0.792 | -0.103267 | 0.017134 | 1835.506938 | 1.67E-09 | 0.015778 | 0.0167386 | 0.776631 | 0.35 |
| rs202535 | A | C | 0.828 | -0.141437 | 0.018453 | 2983.199554 | 1.79E-14 | 0.00547177 | 0.0189835 | 0.840866 | 0.77 |
| rs2111485 | G | A | 0.604 | 0.127631 | 0.014455 | 4088.44115 | 1.05E-18 | -0.00375401 | 0.0142131 | 0.608778 | 0.8 |
| rs2188962 | T | C | 0.404 | 0.07946 | 0.014096 | 1587.680337 | 1.73E-08 | 0.000657125 | 0.0140286 | 0.428982 | 0.95 |
| rs229527 | A | C | 0.416 | 0.104077 | 0.014138 | 2754.376521 | 1.82E-13 | -0.011712 | 0.0140551 | 0.422497 | 0.38 |
| rs231972 | C | A | 0.118 | 0.170939 | 0.021081 | 3185.651486 | 5.12E-16 | -0.00968297 | 0.0210373 | 0.124825 | 0.68 |
| rs238265 | G | T | 0.695 | -0.090825 | 0.015158 | 1826.975643 | 2.07E-09 | 0.0178235 | 0.0152238 | 0.70139 | 0.24 |
| rs2476601 | G | A | 0.886 | -0.64304 | 0.020896 | 47447.38613 | 5.96E-208 | -0.0580848 | 0.0229145 | 0.898197 | 0.011 |
| rs2493411 | C | T | 0.132 | 0.127063 | 0.022335 | 1933.11368 | 1.28E-08 | 0.0331881 | 0.0210336 | 0.124614 | 0.11 |
| rs2543537 | T | C | 0.46 | -0.083441 | 0.014316 | 1806.887828 | 5.59E-09 | 0.00410539 | 0.0140967 | 0.445188 | 0.78 |
| rs2596544 | A | T | 0.221 | 0.721034 | 0.01891 | 113505.7424 | 0 | 0.0362772 | 0.0168995 | 0.21495 | 0.034 |
| rs2611211 | T | C | 0.824 | -0.143854 | 0.018689 | 3143.505085 | 1.39E-14 | -0.00797703 | 0.0190974 | 0.843297 | 0.69 |
| rs28367848 | T | C | 0.121 | -0.246085 | 0.018135 | 6793.462 | 6.06E-42 | -0.041402 | 0.0289499 | 0.062618 | 0.15 |
| rs3024493 | A | C | 0.154 | -0.163855 | 0.019641 | 3667.543074 | 7.27E-17 | -0.0033762 | 0.0192254 | 0.154185 | 0.86 |
| rs3087243 | A | G | 0.422 | -0.19913 | 0.014202 | 10268.63558 | 1.16E-44 | -0.0228927 | 0.0139505 | 0.449862 | 0.1 |
| rs3184504 | C | T | 0.533 | -0.231498 | 0.014086 | 14269.15522 | 1.08E-60 | -0.00934912 | 0.0138805 | 0.517363 | 0.53 |
| rs34536443 | C | G | 0.0427 | -0.385331 | 0.038519 | 6396.813478 | 1.47E-23 | 0.00518692 | 0.0342253 | 0.045704 | 0.88 |
| rs34593439 | A | G | 0.108 | -0.218071 | 0.024119 | 4813.908327 | 1.55E-19 | -0.0127428 | 0.0227307 | 0.10443 | 0.57 |
| rs35327136 | A | C | 0.156 | -0.119196 | 0.018978 | 1954.94291 | 3.37E-10 | 0.0281282 | 0.017916 | 0.184777 | 0.12 |
| rs3802214 | C | T | 0.799 | -0.106609 | 0.01923 | 1907.368936 | 2.96E-08 | 0.027027 | 0.0180217 | 0.818432 | 0.14 |
| rs3842753 | G | T | 0.731 | 0.710531 | 0.018519 | 128965.6515 | 0 | 0.00681069 | 0.015389 | 0.710415 | 0.67 |
| rs41295159 | G | C | 0.00895 | -0.699552 | 0.090252 | 4558.911352 | 9.11E-15 | 0.00271284 | 0.0672992 | 0.011001 | 0.98 |
| rs4490209 | G | C | 0.36 | -0.084522 | 0.015457 | 1719.371964 | 4.55E-08 | 0.0166321 | 0.0144509 | 0.360695 | 0.25 |
| rs4548024 | C | T | 0.234 | -0.095737 | 0.016703 | 1716.12688 | 9.94E-09 | 0.0304752 | 0.0164702 | 0.23725 | 0.065 |
| rs4820827 | C | T | 0.621 | -0.129657 | 0.014267 | 4152.301567 | 1.01E-19 | 0.0303518 | 0.0142027 | 0.605188 | 0.035 |
| rs55893453 | G | A | 0.202 | 0.094653 | 0.01732 | 1507.978163 | 4.63E-08 | 0.0236495 | 0.0169781 | 0.21356 | 0.16 |
| rs55993634 | G | C | 0.0849 | 0.219371 | 0.024379 | 3922.023424 | 2.29E-19 | 0.0296968 | 0.025588 | 0.082684 | 0.25 |
| rs56994090 | C | T | 0.431 | -0.134255 | 0.014594 | 4643.25746 | 3.60E-20 | -0.0237949 | 0.0140987 | 0.415141 | 0.087 |
| rs57209021 | T | C | 0.226 | 0.100693 | 0.018297 | 1853.13478 | 3.73E-08 | -0.00417533 | 0.0166071 | 0.23056 | 0.8 |
| rs601338 | A | G | 0.479 | 0.127096 | 0.014419 | 4231.247962 | 1.20E-18 | -0.0309067 | 0.0138777 | 0.508992 | 0.028 |
| rs607703 | T | C | 0.484 | 0.092015 | 0.01428 | 2210.897843 | 1.17E-10 | 0.0172419 | 0.0139329 | 0.47325 | 0.21 |
| rs61759532 | T | C | 0.235 | 0.118379 | 0.018587 | 2636.259749 | 1.90E-10 | -0.03656 | 0.0165296 | 0.247778 | 0.03 |
| rs61839660 | T | C | 0.0851 | -0.357441 | 0.026 | 10567.08208 | 5.26E-43 | -0.0301634 | 0.0232823 | 0.098611 | 0.19 |
| rs62395823 | A | G | 0.0427 | -0.699325 | 0.042518 | 21680.55784 | 8.71E-61 | 0.0125852 | 0.0343964 | 0.042491 | 0.7 |
| rs6434435 | A | G | 0.16 | -0.122856 | 0.019089 | 2120.671516 | 1.23E-10 | -0.0260764 | 0.018535 | 0.17511 | 0.17 |
| rs663743 | A | G | 0.349 | -0.099964 | 0.015092 | 2374.575426 | 3.50E-11 | 0.00711269 | 0.0146758 | 0.340981 | 0.65 |
| rs6908626 | T | G | 0.166 | 0.202923 | 0.01852 | 6003.888253 | 6.15E-28 | 0.0157027 | 0.0182164 | 0.179081 | 0.37 |
| rs7068821 | T | G | 0.251 | -0.165103 | 0.016333 | 5390.827935 | 5.06E-24 | -0.0151602 | 0.0158848 | 0.258089 | 0.34 |
| rs7130222 | G | T | 0.308 | -0.091985 | 0.016179 | 1884.412095 | 1.30E-08 | -0.0228533 | 0.0148557 | 0.324937 | 0.13 |
| rs722988 | C | T | 0.353 | 0.082649 | 0.014412 | 1629.398111 | 9.77E-09 | 0.0124405 | 0.0144752 | 0.365212 | 0.43 |
| rs7237497 | C | T | 0.839 | -0.220466 | 0.018635 | 6926.716691 | 2.71E-32 | -0.00911 | 0.0187221 | 0.83458 | 0.62 |
| rs7668577 | C | A | 0.312 | 0.093652 | 0.015203 | 1967.575089 | 7.27E-10 | -0.00192433 | 0.0149927 | 0.311823 | 0.91 |
| rs7776597 | G | A | 0.959 | 0.244413 | 0.036372 | 2457.037846 | 1.82E-11 | -0.0289795 | 0.0334169 | 0.9544136 | 0.38 |
| rs7795896 | T | C | 0.692 | -0.135435 | 0.016416 | 4102.452061 | 1.58E-16 | 0.00162812 | 0.0152331 | 0.704325 | 0.9 |
| rs78325861 | G | C | 0.039 | -0.282082 | 0.042195 | 3123.579924 | 2.31E-11 | 0.0024676 | 0.0350667 | 0.040749 | 0.96 |
| rs8046043 | C | G | 0.392 | -0.084587 | 0.015175 | 1781.543456 | 2.49E-08 | -0.0032867 | 0.0141023 | 0.416846 | 0.82 |
| rs855330 | C | T | 0.259 | 0.111208 | 0.016916 | 2482.975003 | 4.89E-11 | 0.0143185 | 0.0164925 | 0.232002 | 0.37 |
| rs9267951 | T | C | 0.276 | 0.691817 | 0.016264 | 123124.7872 | 0 | 0.0474039 | 0.0156219 | 0.271383 | 0.0025 |
| rs9273311 | A | G | 0.617 | 1.10584 | 0.019133 | 712905.1221 | 0 | 0.0319499 | 0.0142501 | 0.608689 | 0.025 |
| rs9385401 | T | C | 0.454 | 0.12042 | 0.014827 | 3769.597838 | 4.60E-16 | -0.0192202 | 0.0139429 | 0.454447 | 0.16 |
| rs9517712 | C | T | 0.741 | -0.102063 | 0.015805 | 2089.827285 | 1.06E-10 | 0.015995 | 0.0160383 | 0.749396 | 0.34 |

Supplement table 5. Summary data of SNPs used in MR analysis of IVs associated with type 2 diabetes-frozen shoulder.

| **SNP** | **Effect Allele** | **Other Allele** | **EAF** | **Beta Estimate** | **SE** | **F-statistic** | **P value** | **beta.FS** | **se.FS** | **eaf.FS** | **Pval.FS** |
| --- | --- | --- | --- | --- | --- | --- | --- | --- | --- | --- | --- |
| rs1002389 | G | C | 0.392781 | -0.0511 | 0.0064 | 611.1982231 | 1.41E-15 | -0.0126527 | 0.0145663 | 0.347868 | 0.41 |
| rs10062657 | A | C | 0.799903 | -0.0417 | 0.0075 | 272.9574846 | 2.70E-08 | 0.0202461 | 0.0238769 | 0.9046166 | 0.4 |
| rs10169613 | T | C | 0.446602 | -0.0386 | 0.0063 | 361.2068726 | 8.96E-10 | 0.0114257 | 0.0139229 | 0.475271 | 0.42 |
| rs10173251 | G | C | 0.11235 | -0.1062 | 0.0141 | 1104.956318 | 5.00E-14 | 0.00734176 | 0.0206808 | 0.128785 | 0.73 |
| rs10201401 | A | G | 0.787528 | 0.0421 | 0.0075 | 290.8658645 | 1.98E-08 | -0.00161818 | 0.0212706 | 0.878441 | 0.95 |
| rs10268294 | A | C | 0.796446 | 0.0454 | 0.0081 | 327.7488682 | 2.08E-08 | 0.0174692 | 0.0169502 | 0.785009 | 0.29 |
| rs10408179 | C | T | 0.38792 | -0.0675 | 0.0067 | 1062.678185 | 7.15E-24 | 0.000720362 | 0.0139496 | 0.440095 | 0.95 |
| rs1046316 | G | A | 0.745203 | 0.0999 | 0.0087 | 1864.45595 | 1.61E-30 | 0.0160644 | 0.0148747 | 0.677834 | 0.27 |
| rs10830963 | G | C | 0.330035 | 0.0504 | 0.0065 | 551.1432473 | 8.92E-15 | 0.0392793 | 0.0155218 | 0.275343 | 0.012 |
| rs10900325 | C | T | 0.841826 | -0.0557 | 0.0082 | 405.2564856 | 1.10E-11 | -0.0411805 | 0.0209846 | 0.874514 | 0.051 |
| rs10938398 | A | G | 0.406542 | 0.0491 | 0.0065 | 570.777803 | 4.23E-14 | 0.0109679 | 0.014045 | 0.433782 | 0.44 |
| rs10950494 | A | G | 0.450764 | 0.0361 | 0.0063 | 316.4507553 | 1.00E-08 | 0.0131293 | 0.0140838 | 0.433983 | 0.36 |
| rs10965250 | A | G | 0.235558 | -0.1644 | 0.0073 | 4817.227803 | 2.61E-112 | -0.029051 | 0.0184456 | 0.17066 | 0.12 |
| rs111640200 | G | A | 0.305886 | 0.0413 | 0.0067 | 355.2289572 | 7.08E-10 | 0.0153428 | 0.0170188 | 0.211296 | 0.37 |
| rs1117610 | T | A | 0.312695 | 0.0418 | 0.0069 | 368.3430413 | 1.38E-09 | -0.0223569 | 0.01667 | 0.227921 | 0.17 |
| rs112108223 | A | G | 0.0147015 | -0.2373 | 0.0344 | 800.8242251 | 5.26E-12 | -0.0167992 | 0.065398 | 0.012 | 0.75 |
| rs11257655 | T | C | 0.281992 | 0.1084 | 0.0069 | 2343.145528 | 1.29E-55 | 0.0105001 | 0.0170918 | 0.208151 | 0.54 |
| rs1127215 | T | C | 0.368543 | -0.042 | 0.0066 | 402.708155 | 1.97E-10 | -0.00223611 | 0.0140603 | 0.420111 | 0.9 |
| rs1128249 | T | G | 0.300292 | -0.0786 | 0.0078 | 1275.668546 | 6.99E-24 | -0.00824446 | 0.0142099 | 0.392443 | 0.57 |
| rs114322470 | G | T | 0.0204657 | -0.2281 | 0.0301 | 1024.488084 | 3.51E-14 | -0.112285 | 0.0511802 | 0.019303 | 0.026 |
| rs115018313 | C | T | 0.0309476 | 0.284 | 0.0238 | 2382.43022 | 7.99E-33 | 0.0328644 | 0.0461777 | 0.023114 | 0.48 |
| rs11514706 | C | A | 0.500501 | 0.0601 | 0.0063 | 886.7000669 | 1.43E-21 | -0.00973585 | 0.01393 | 0.524124 | 0.48 |
| rs11607147 | C | T | 0.684232 | -0.0411 | 0.0068 | 357.9937486 | 1.50E-09 | -0.00511631 | 0.0149347 | 0.673399 | 0.7 |
| rs11651052 | G | A | 0.589657 | -0.091 | 0.0065 | 1971.861126 | 1.56E-44 | -0.00623678 | 0.0139529 | 0.524121 | 0.65 |
| rs116947250 | A | T | 0.031514 | 0.0849 | 0.0155 | 215.7280231 | 4.32E-08 | 0.100857 | 0.0670022 | 0.010933 | 0.13 |
| rs117430555 | T | G | 0.0336968 | 0.1366 | 0.0235 | 596.2586784 | 6.14E-09 | -0.0192997 | 0.0397622 | 0.032177 | 0.64 |
| rs11819995 | T | C | 0.213049 | 0.0493 | 0.0077 | 399.7406345 | 1.53E-10 | 0.0286417 | 0.0165572 | 0.229041 | 0.085 |
| rs11846158 | G | A | 0.283548 | 0.037 | 0.0068 | 272.7482325 | 5.29E-08 | -0.00246548 | 0.0167786 | 0.219926 | 0.88 |
| rs12001437 | C | T | 0.413559 | 0.0367 | 0.0064 | 320.3914616 | 9.79E-09 | 0.0122473 | 0.014402 | 0.366965 | 0.39 |
| rs12108088 | A | G | 0.439211 | -0.0365 | 0.0065 | 321.8449609 | 1.96E-08 | -0.0218398 | 0.014185 | 0.402298 | 0.12 |
| rs1215468 | G | A | 0.287639 | -0.0749 | 0.0069 | 1129.312973 | 1.89E-27 | -0.017869 | 0.0153445 | 0.292183 | 0.24 |
| rs12441261 | A | C | 0.248102 | -0.0435 | 0.0074 | 346.2402896 | 4.14E-09 | -0.010056 | 0.020663 | 0.131136 | 0.64 |
| rs12453376 | G | A | 0.2041 | 0.0581 | 0.0076 | 538.0634601 | 2.09E-14 | 0.000373077 | 0.0183739 | 0.172733 | 0.98 |
| rs12546365 | C | T | 0.55818 | -0.0381 | 0.0065 | 351.1428493 | 4.59E-09 | -0.0034985 | 0.0138974 | 0.496654 | 0.81 |
| rs12578595 | T | C | 0.222896 | -0.0715 | 0.0073 | 869.4938356 | 1.19E-22 | -0.00401179 | 0.0174273 | 0.196798 | 0.81 |
| rs1260326 | C | T | 0.571429 | 0.071 | 0.0064 | 1213.049599 | 1.35E-28 | 0.00271136 | 0.0141938 | 0.605604 | 0.84 |
| rs12625671 | C | T | 0.203237 | 0.0565 | 0.0076 | 507.2023261 | 1.05E-13 | -0.00572585 | 0.0225831 | 0.106251 | 0.79 |
| rs12692723 | G | C | 0.311541 | 0.0436 | 0.0068 | 399.96661 | 1.44E-10 | 0.00394257 | 0.0160155 | 0.273056 | 0.83 |
| rs12698897 | A | T | 0.256189 | 0.0586 | 0.0071 | 642.2294693 | 1.54E-16 | -0.0112515 | 0.0161395 | 0.246286 | 0.49 |
| rs12763565 | G | T | 0.134488 | 0.0624 | 0.01 | 444.6546537 | 4.38E-10 | 0.0113583 | 0.018911 | 0.160053 | 0.57 |
| rs12910361 | G | A | 0.62609 | 0.0696 | 0.0066 | 1114.067807 | 5.33E-26 | 0.0253594 | 0.015343 | 0.713378 | 0.099 |
| rs13073970 | T | G | 0.193647 | -0.0499 | 0.008 | 381.3977231 | 4.45E-10 | -0.0284402 | 0.0171243 | 0.208137 | 0.097 |
| rs13086642 | A | G | 0.092455 | -0.0599 | 0.0101 | 295.2683996 | 3.02E-09 | -0.0210091 | 0.0274745 | 0.068813 | 0.47 |
| rs13162708 | T | C | 0.208377 | 0.0546 | 0.0075 | 482.4852004 | 3.34E-13 | 0.0131213 | 0.0205614 | 0.130352 | 0.54 |
| rs13266634 | T | C | 0.348803 | -0.1053 | 0.0065 | 2481.107777 | 5.04E-59 | -0.00542742 | 0.0150139 | 0.310583 | 0.72 |
| rs13292347 | C | T | 0.0708271 | -0.0727 | 0.0122 | 341.1695163 | 2.54E-09 | 0.0515749 | 0.0293793 | 0.059503 | 0.084 |
| rs1362891 | A | G | 0.0714984 | -0.0821 | 0.0117 | 438.9927167 | 2.27E-12 | 0.0226249 | 0.0352763 | 0.040278 | 0.52 |
| rs139722172 | G | C | 0.186009 | -0.0786 | 0.0111 | 918.5759548 | 1.43E-12 | 0.0363779 | 0.018406 | 0.185594 | 0.05 |
| rs141330165 | T | C | 0.0560573 | -0.1202 | 0.0168 | 750.5065391 | 8.38E-13 | 0.563765 | 0.542774 | 0.000173 | 0.3 |
| rs1426371 | A | G | 0.337897 | -0.0486 | 0.0066 | 518.4953123 | 1.79E-13 | 0.00082056 | 0.0159654 | 0.26025 | 0.99 |
| rs142682088 | A | G | 0.0401343 | -0.1263 | 0.0217 | 603.0733462 | 5.88E-09 | -0.057489 | 0.0359747 | 0.039083 | 0.1 |
| rs143708136 | T | C | 0.0116967 | 0.2321 | 0.042 | 611.1506732 | 3.27E-08 | -0.0870171 | 0.0623137 | 0.013195 | 0.17 |
| rs145109691 | G | T | 0.122755 | 0.0637 | 0.01 | 428.6687626 | 1.89E-10 | 0.0352196 | 0.024929 | 0.085144 | 0.17 |
| rs149336329 | T | G | 0.0459427 | -0.1063 | 0.0148 | 485.9494506 | 6.85E-13 | -0.013403 | 0.0333095 | 0.045648 | 0.71 |
| rs1515104 | A | T | 0.719318 | 0.0754 | 0.0078 | 1127.66344 | 4.18E-22 | -0.000224377 | 0.0145682 | 0.652218 | 0.96 |
| rs1567353 | G | C | 0.340343 | 0.0403 | 0.0065 | 357.6556519 | 5.65E-10 | -0.0007185 | 0.0150979 | 0.307786 | 0.97 |
| rs1574285 | T | G | 0.583008 | -0.065 | 0.0063 | 1008.846769 | 5.88E-25 | -0.00133404 | 0.0142012 | 0.600179 | 0.93 |
| rs16858462 | C | G | 0.0525964 | 0.0738 | 0.0134 | 266.1599755 | 3.64E-08 | 0.0317075 | 0.0576721 | 0.014707 | 0.56 |
| rs17036160 | T | C | 0.105331 | -0.1092 | 0.0112 | 1103.936808 | 1.84E-22 | 0.027814 | 0.0215495 | 0.118253 | 0.19 |
| rs1707498 | A | C | 0.449132 | -0.0499 | 0.0063 | 604.5903984 | 2.36E-15 | 0.0152932 | 0.0140178 | 0.434101 | 0.27 |
| rs17101591 | T | C | 0.0577437 | -0.0683 | 0.0114 | 248.9079452 | 2.08E-09 | 0.115756 | 0.0606885 | 0.013336 | 0.056 |
| rs17168486 | T | C | 0.238504 | 0.0626 | 0.0071 | 698.6083541 | 1.18E-18 | -0.0113289 | 0.0184132 | 0.173497 | 0.52 |
| rs17496664 | T | C | 0.277089 | -0.0468 | 0.0085 | 430.4079107 | 3.67E-08 | -0.0157288 | 0.0142554 | 0.392147 | 0.26 |
| rs1776899 | A | G | 0.943992 | 0.0899 | 0.012 | 419.1912908 | 6.80E-14 | -0.0134655 | 0.0403041 | 0.9694442 | 0.73 |
| rs1794138 | G | T | 0.516893 | -0.0449 | 0.0078 | 493.9435662 | 8.59E-09 | -0.010785 | 0.0139099 | 0.515794 | 0.45 |
| rs1861410 | T | C | 0.573342 | -0.0372 | 0.0064 | 332.0296284 | 6.15E-09 | -1.51E-05 | 0.0140202 | 0.555424 | 0.96 |
| rs1966265 | A | G | 0.322278 | -0.0465 | 0.0068 | 463.3420629 | 8.02E-12 | 0.0236138 | 0.0160909 | 0.246868 | 0.14 |
| rs1977832 | T | G | 0.502983 | -0.1118 | 0.0065 | 3082.009201 | 2.66E-66 | 0.00137848 | 0.0141204 | 0.408618 | 0.94 |
| rs2010825 | C | T | 0.519789 | 0.0411 | 0.0063 | 413.6303503 | 6.85E-11 | 0.0226818 | 0.0140256 | 0.483971 | 0.1 |
| rs2034761 | G | T | 0.429369 | -0.0375 | 0.0063 | 337.9489751 | 2.64E-09 | 0.00220971 | 0.0141667 | 0.401893 | 0.87 |
| rs2115107 | A | G | 0.372125 | 0.0451 | 0.0065 | 466.2633583 | 3.96E-12 | -0.0192319 | 0.0142779 | 0.381874 | 0.18 |
| rs2237897 | T | C | 0.249812 | -0.2678 | 0.009 | 13537.59385 | 1.47E-194 | -0.000476281 | 0.0352214 | 0.041941 | 0.98 |
| rs2270162 | G | C | 0.0724056 | 0.0683 | 0.0107 | 307.2890478 | 1.73E-10 | -0.0428525 | 0.0382077 | 0.034261 | 0.26 |
| rs2289488 | C | G | 0.370273 | 0.0448 | 0.0069 | 459.1348314 | 8.43E-11 | -0.00481672 | 0.0145418 | 0.394427 | 0.73 |
| rs2382249 | G | A | 0.217601 | -0.0465 | 0.0082 | 361.0922143 | 1.42E-08 | 0.0165909 | 0.0207115 | 0.128737 | 0.39 |
| rs2578011 | T | C | 0.204526 | -0.0693 | 0.0074 | 767.0494399 | 7.61E-21 | -0.00545172 | 0.0202407 | 0.13769 | 0.78 |
| rs2583938 | A | T | 0.185271 | 0.0692 | 0.0077 | 709.519408 | 2.54E-19 | 0.0242831 | 0.020523 | 0.132769 | 0.24 |
| rs2648731 | A | G | 0.240353 | 0.0647 | 0.0072 | 750.3044006 | 2.56E-19 | 0.000937217 | 0.0166784 | 0.225617 | 0.92 |
| rs2706710 | T | C | 0.161364 | 0.0495 | 0.0088 | 325.2234347 | 1.86E-08 | -0.0257678 | 0.0180761 | 0.181253 | 0.15 |
| rs2768344 | T | C | 0.833624 | 0.0459 | 0.0084 | 286.5783099 | 4.65E-08 | -0.00710465 | 0.0184899 | 0.826982 | 0.69 |
| rs2796441 | A | G | 0.472771 | -0.0679 | 0.0063 | 1128.995377 | 4.38E-27 | 0.0200264 | 0.0140416 | 0.419314 | 0.15 |
| rs28375915 | A | G | 0.476839 | -0.0498 | 0.0077 | 607.1649663 | 9.96E-11 | -0.00677027 | 0.0146153 | 0.493395 | 0.63 |
| rs28637955 | G | A | 0.373478 | -0.067 | 0.0118 | 1031.733308 | 1.36E-08 | -0.0212252 | 0.0144824 | 0.373351 | 0.14 |
| rs28642213 | G | A | 0.773252 | 0.1006 | 0.0079 | 1745.450971 | 3.82E-37 | 0.0155728 | 0.0160455 | 0.750681 | 0.33 |
| rs3110641 | G | A | 0.76149 | 0.0598 | 0.0072 | 637.4422577 | 9.94E-17 | 0.00399879 | 0.016931 | 0.783076 | 0.79 |
| rs340874 | C | T | 0.493303 | 0.0535 | 0.0063 | 702.2547657 | 2.03E-17 | -0.0121052 | 0.0139994 | 0.569628 | 0.38 |
| rs348330 | A | G | 0.543403 | -0.0539 | 0.0068 | 707.5584966 | 2.25E-15 | 0.00219405 | 0.0144703 | 0.633978 | 0.87 |
| rs34872471 | C | T | 0.207525 | 0.2972 | 0.0091 | 14664.28986 | 5.90E-234 | -0.0101625 | 0.0152964 | 0.291311 | 0.5 |
| rs35352848 | C | T | 0.213447 | -0.0881 | 0.0078 | 1280.578946 | 1.39E-29 | -0.0258076 | 0.0172064 | 0.204177 | 0.13 |
| rs35385487 | G | A | 0.11622 | -0.076 | 0.0089 | 582.1984122 | 1.35E-17 | 0.021568 | 0.0457999 | 0.023502 | 0.63 |
| rs35473599 | A | G | 0.182945 | 0.0553 | 0.0093 | 448.4587488 | 2.74E-09 | 0.00112265 | 0.0161535 | 0.243835 | 0.93 |
| rs35895680 | A | C | 0.24391 | -0.0584 | 0.0087 | 617.2749198 | 1.91E-11 | 0.00611463 | 0.0148906 | 0.326705 | 0.67 |
| rs35917376 | A | T | 0.717917 | 0.0428 | 0.0072 | 363.8850451 | 2.77E-09 | -0.0206606 | 0.0161608 | 0.745547 | 0.2 |
| rs3743140 | A | G | 0.152268 | 0.0623 | 0.0082 | 491.566048 | 3.02E-14 | -0.0221229 | 0.0224935 | 0.107189 | 0.32 |
| rs3751297 | A | G | 0.241048 | 0.052 | 0.0081 | 485.3528612 | 1.36E-10 | -0.0185986 | 0.0155621 | 0.28746 | 0.22 |
| rs3887925 | T | C | 0.49964 | 0.053 | 0.0063 | 689.2949489 | 4.01E-17 | -0.0114197 | 0.0139836 | 0.549858 | 0.42 |
| rs3918296 | G | C | 0.0192566 | -0.1776 | 0.0252 | 584.5771338 | 1.82E-12 | 0.0267263 | 0.043742 | 0.026413 | 0.56 |
| rs39254 | T | G | 0.46123 | 0.0401 | 0.0067 | 391.976554 | 2.16E-09 | 0.0131849 | 0.0139341 | 0.530969 | 0.31 |
| rs392794 | T | C | 0.659966 | 0.06 | 0.0066 | 793.14368 | 9.82E-20 | -0.0189717 | 0.0159356 | 0.74677 | 0.24 |
| rs4273712 | G | A | 0.320615 | 0.0577 | 0.0066 | 711.8441892 | 2.28E-18 | 0.00932925 | 0.0157149 | 0.265831 | 0.56 |
| rs429358 | C | T | 0.14701 | -0.0739 | 0.0092 | 672.1684312 | 9.54E-16 | -0.017185 | 0.0191894 | 0.154996 | 0.36 |
| rs4517619 | G | T | 0.384609 | 0.0395 | 0.007 | 362.233682 | 1.67E-08 | 0.00806528 | 0.0158422 | 0.263019 | 0.63 |
| rs4632135 | C | T | 0.0339896 | 0.0832 | 0.0152 | 222.8820792 | 4.41E-08 | -0.0109967 | 0.0474121 | 0.021958 | 0.83 |
| rs4731701 | T | C | 0.43764 | -0.0484 | 0.0065 | 565.7523805 | 9.61E-14 | -0.0077731 | 0.013915 | 0.49465 | 0.57 |
| rs4795608 | G | A | 0.350995 | 0.037 | 0.0065 | 305.8627276 | 1.25E-08 | -0.0241622 | 0.0149843 | 0.325841 | 0.11 |
| rs4854343 | A | G | 0.849205 | 0.0688 | 0.0091 | 594.8484701 | 4.02E-14 | -0.021534 | 0.0183751 | 0.827758 | 0.24 |
| rs4901812 | A | G | 0.333674 | -0.0408 | 0.0067 | 363.0397866 | 1.13E-09 | 0.0125583 | 0.0161891 | 0.246319 | 0.44 |
| rs504652 | G | T | 0.164415 | 0.0488 | 0.0087 | 320.8922012 | 2.03E-08 | -0.0188755 | 0.0178261 | 0.187507 | 0.28 |
| rs5215 | T | C | 0.616015 | -0.0623 | 0.0064 | 901.5358215 | 2.15E-22 | -0.0409763 | 0.01448 | 0.642114 | 0.0045 |
| rs543159 | A | C | 0.427571 | -0.0364 | 0.0065 | 318.0662438 | 2.14E-08 | -0.00286206 | 0.0139365 | 0.471978 | 0.84 |
| rs554833 | T | C | 0.376316 | 0.0443 | 0.0064 | 451.8852591 | 4.46E-12 | 0.0015798 | 0.0148979 | 0.318074 | 0.92 |
| rs56094641 | G | A | 0.352899 | 0.1166 | 0.0069 | 3062.160098 | 4.61E-64 | -0.00972571 | 0.0141452 | 0.404251 | 0.5 |
| rs56218782 | C | T | 0.312633 | -0.039 | 0.0068 | 320.5825525 | 9.73E-09 | 0.0269182 | 0.0148651 | 0.326343 | 0.073 |
| rs56348580 | C | G | 0.301336 | -0.0631 | 0.0093 | 823.0192535 | 1.16E-11 | -0.00855303 | 0.015077 | 0.307619 | 0.56 |
| rs5762867 | G | A | 0.362109 | 0.0389 | 0.007 | 342.84031 | 2.74E-08 | -0.004709 | 0.0165522 | 0.228328 | 0.76 |
| rs576674 | A | G | 0.856359 | -0.0802 | 0.0091 | 776.7369544 | 1.22E-18 | 0.00112437 | 0.0185813 | 0.832576 | 0.96 |
| rs58090211 | A | C | 0.0766814 | -0.0807 | 0.0136 | 452.3687029 | 2.96E-09 | -0.0688683 | 1.41139 | 3.60E-05 | 0.97 |
| rs6066137 | T | C | 0.263925 | -0.0815 | 0.0129 | 1268.070072 | 2.65E-10 | 0.0113201 | 0.0158139 | 0.26293 | 0.47 |
| rs62262091 | T | C | 0.0970158 | 0.0876 | 0.0148 | 659.8091183 | 3.24E-09 | 0.0573313 | 0.0242296 | 0.095336 | 0.019 |
| rs62319060 | C | T | 0.470491 | 0.0371 | 0.0066 | 336.3361966 | 1.90E-08 | -0.0118065 | 0.014444 | 0.361642 | 0.41 |
| rs62405419 | T | G | 0.156975 | 0.0565 | 0.0082 | 414.4175164 | 5.57E-12 | -0.0284674 | 0.0216362 | 0.116894 | 0.19 |
| rs62508166 | A | G | 0.178231 | -0.1018 | 0.0085 | 1492.286205 | 4.72E-33 | 0.0220151 | 0.0178002 | 0.198196 | 0.22 |
| rs633715 | C | T | 0.218583 | 0.0491 | 0.0075 | 403.9459074 | 5.88E-11 | 0.0270855 | 0.0169986 | 0.211687 | 0.11 |
| rs6453133 | G | A | 0.357642 | -0.0438 | 0.0065 | 432.3744619 | 1.60E-11 | -0.0194671 | 0.0153239 | 0.28722 | 0.21 |
| rs6496742 | T | C | 0.303172 | 0.0582 | 0.0067 | 702.4021353 | 3.74E-18 | 0.0242061 | 0.0164335 | 0.232518 | 0.15 |
| rs6554060 | G | A | 0.376916 | 0.0366 | 0.0066 | 308.5530634 | 2.93E-08 | 0.0289833 | 0.0152534 | 0.292873 | 0.057 |
| rs6567160 | C | T | 0.218731 | 0.0785 | 0.0076 | 1034.353664 | 5.21E-25 | 0.00280905 | 0.016428 | 0.232673 | 0.89 |
| rs6813195 | T | C | 0.353285 | -0.062 | 0.0066 | 862.3592072 | 5.78E-21 | 0.00423111 | 0.0158859 | 0.276659 | 0.77 |
| rs6853272 | T | C | 0.923642 | -0.0718 | 0.0103 | 356.6368425 | 3.15E-12 | -0.00305425 | 0.0415888 | 0.9713283 | 0.91 |
| rs6878122 | A | G | 0.778957 | -0.0547 | 0.0092 | 505.493759 | 2.75E-09 | -0.0100526 | 0.0148986 | 0.680597 | 0.48 |
| rs6885132 | G | C | 0.195176 | -0.0449 | 0.0077 | 310.5977774 | 5.50E-09 | -0.0314755 | 0.0235316 | 0.09985 | 0.18 |
| rs6985028 | A | G | 0.656712 | -0.0408 | 0.0073 | 368.1147032 | 2.28E-08 | 0.0317551 | 0.0139719 | 0.55954 | 0.024 |
| rs7030811 | A | T | 0.0395381 | -0.0944 | 0.0151 | 331.9228422 | 4.06E-10 | 0.0114148 | 0.0389149 | 0.033009 | 0.79 |
| rs7107784 | A | G | 0.774621 | -0.0532 | 0.0083 | 484.7954666 | 1.46E-10 | 0.0238249 | 0.0153378 | 0.71308 | 0.12 |
| rs7132351 | A | G | 0.17439 | 0.063 | 0.0079 | 560.7605303 | 1.53E-15 | -0.000877473 | 0.0206688 | 0.131005 | 0.96 |
| rs7132908 | A | G | 0.358104 | 0.0394 | 0.0066 | 350.0092453 | 2.38E-09 | -0.0227233 | 0.0142834 | 0.384388 | 0.11 |
| rs7141 | A | G | 0.6394 | -0.0357 | 0.0065 | 288.1995379 | 3.97E-08 | -0.03063 | 0.0149037 | 0.672511 | 0.04 |
| rs71424153 | C | G | 0.259162 | -0.0429 | 0.007 | 346.5926618 | 8.87E-10 | -0.00617667 | 0.0162481 | 0.239613 | 0.68 |
| rs7172432 | G | A | 0.452818 | -0.0604 | 0.0063 | 887.6022297 | 9.04E-22 | -0.0176929 | 0.0140258 | 0.428766 | 0.21 |
| rs7250869 | C | T | 0.628785 | -0.0488 | 0.0065 | 545.448305 | 6.02E-14 | 0.00884288 | 0.015 | 0.689044 | 0.55 |
| rs72640313 | A | G | 0.116012 | -0.0615 | 0.0094 | 380.4869221 | 6.05E-11 | 0.0236655 | 0.0230493 | 0.101541 | 0.3 |
| rs72802365 | C | G | 0.0619909 | -0.1168 | 0.0146 | 778.7775603 | 1.24E-15 | 0.0269733 | 0.0259073 | 0.078278 | 0.3 |
| rs72830608 | T | G | 0.305978 | 0.0482 | 0.0077 | 484.0491102 | 3.86E-10 | -0.00216289 | 0.0168363 | 0.220335 | 0.9 |
| rs72906810 | G | A | 0.0894907 | -0.0876 | 0.0124 | 613.6448983 | 1.61E-12 | 0.00651016 | 0.0244477 | 0.088371 | 0.77 |
| rs72951548 | T | C | 0.188488 | -0.0627 | 0.0078 | 590.1189166 | 9.10E-16 | -0.0157746 | 0.019189 | 0.155848 | 0.4 |
| rs72964564 | C | A | 0.231426 | -0.1004 | 0.0104 | 1763.715642 | 4.73E-22 | -0.0115003 | 0.0160749 | 0.250964 | 0.48 |
| rs73221113 | A | G | 0.039939 | 0.0933 | 0.016 | 327.3802637 | 5.50E-09 | 0.0418887 | 0.0361389 | 0.038294 | 0.25 |
| rs73347525 | G | A | 0.201505 | -0.0536 | 0.008 | 453.5158673 | 2.08E-11 | -0.00948521 | 0.0184331 | 0.182502 | 0.6 |
| rs74862545 | T | C | 0.0223105 | -0.2052 | 0.0301 | 901.9171644 | 9.28E-12 | 0.0313153 | 0.0470169 | 0.023519 | 0.51 |
| rs75418188 | T | C | 0.0276409 | 0.1337 | 0.0157 | 471.370461 | 1.65E-17 | -0.0714007 | 0.0862422 | 0.006526 | 0.41 |
| rs75872811 | G | A | 0.0871287 | -0.0727 | 0.0102 | 412.3899535 | 1.02E-12 | 0.0498166 | 0.0317079 | 0.050912 | 0.12 |
| rs76177300 | A | G | 0.0551313 | 0.1309 | 0.0187 | 876.4520246 | 2.56E-12 | 0.0563872 | 0.0310494 | 0.053697 | 0.074 |
| rs77464186 | C | A | 0.141673 | -0.1028 | 0.01 | 1262.835857 | 8.67E-25 | -0.0323053 | 0.0190851 | 0.156698 | 0.084 |
| rs7773175 | G | C | 0.249461 | -0.0488 | 0.0075 | 437.4276208 | 7.68E-11 | 0.0142328 | 0.0152128 | 0.293389 | 0.33 |
| rs7778167 | A | G | 0.216676 | 0.0571 | 0.0082 | 543.0109212 | 3.32E-12 | 0.0193923 | 0.0158935 | 0.26875 | 0.21 |
| rs78470967 | A | T | 0.042318 | -0.1272 | 0.0217 | 643.5670311 | 4.58E-09 | 0.0543278 | 0.0348923 | 0.043189 | 0.12 |
| rs7925578 | T | G | 0.463149 | 0.0675 | 0.0073 | 1112.936383 | 2.32E-20 | 0.0139009 | 0.0147447 | 0.341234 | 0.33 |
| rs8043085 | T | G | 0.301228 | 0.0457 | 0.0068 | 431.2691726 | 1.81E-11 | -0.00418506 | 0.0164524 | 0.23326 | 0.8 |
| rs8100204 | A | G | 0.169058 | 0.0552 | 0.0082 | 419.9133884 | 1.68E-11 | -0.00399366 | 0.0201036 | 0.146474 | 0.84 |
| rs8100562 | T | C | 0.170998 | 0.0559 | 0.0088 | 434.5685861 | 2.12E-10 | 0.00973379 | 0.0174968 | 0.200269 | 0.55 |
| rs860262 | A | C | 0.420673 | -0.0668 | 0.0068 | 1068.243142 | 8.92E-23 | -0.041296 | 0.0138811 | 0.501982 | 0.0031 |
| rs9265433 | C | T | 0.445483 | 0.0625 | 0.0067 | 947.6504446 | 1.08E-20 | 0.0339217 | 0.0143191 | 0.477905 | 0.019 |
| rs9304665 | A | T | 0.752917 | 0.0545 | 0.0098 | 542.2085586 | 2.68E-08 | 0.0341846 | 0.0163955 | 0.764646 | 0.035 |
| rs9368222 | A | C | 0.318552 | 0.1551 | 0.0066 | 5172.487533 | 4.08E-122 | -0.0113134 | 0.0157787 | 0.26218 | 0.48 |
| rs9379084 | A | G | 0.156857 | -0.0816 | 0.0085 | 864.6775928 | 7.99E-22 | 0.000920236 | 0.0223789 | 0.115581 | 0.97 |
| rs9562987 | A | G | 0.41532 | -0.0413 | 0.0069 | 406.3163294 | 2.16E-09 | -0.00601548 | 0.0148227 | 0.325162 | 0.68 |
| rs972119 | G | A | 0.215533 | -0.0465 | 0.0074 | 358.6040586 | 3.30E-10 | -0.0110357 | 0.0177869 | 0.187729 | 0.55 |
| rs9841201 | A | G | 0.319026 | 0.1259 | 0.0067 | 3398.698471 | 8.94E-79 | 0.0367637 | 0.0149576 | 0.314355 | 0.015 |
| rs9844972 | C | G | 0.0666559 | 0.1036 | 0.0176 | 655.365298 | 3.95E-09 | -0.0162602 | 0.0274328 | 0.071382 | 0.57 |
| rs9873519 | T | C | 0.487086 | 0.0373 | 0.0065 | 340.9361562 | 9.55E-09 | 0.00563345 | 0.0139365 | 0.532893 | 0.67 |

Abbreviations: SE, standard error; EAF, effect allele frequency; SNP, single nucleotide polymorphism; FS, frozen shoulder.

Supplement table 6. Summary data of SNPs used in MR analysis of IVs associated with HDL-frozen shoulder.

| **SNP** | **Effect Allele** | **Other Allele** | **EAF** | **Beta Estimate** | **SE** | **F-statistic** | **P value** | **beta.FS** | **se.FS** | **eaf.FS** | **Pval.FS** |
| --- | --- | --- | --- | --- | --- | --- | --- | --- | --- | --- | --- |
| rs10162642 | A | G | 0.211684 | -0.04792 | 0.0023419 | 309.8451 | 4.60E-93 | -0.0150554 | 0.017087 | 0.2111 | 0.38 |
| rs1054852 | G | A | 0.376936 | 0.035105 | 0.0020654 | 233.9539 | 8.71E-65 | 0.007953 | 0.015068 | 0.375836 | 0.61 |
| rs11045171 | G | A | 0.197605 | 0.028366 | 0.00240558 | 103.0966 | 4.30E-32 | -0.00405765 | 0.017527 | 0.197647 | 0.81 |
| rs112259268 | A | C | 0.028404 | -0.16853 | 0.00575922 | 634.2633 | 3.04E-188 | 0.0171335 | 0.041747 | 0.028585 | 0.65 |
| rs11239536 | A | T | 0.240967 | 0.02875 | 0.00223129 | 122.1733 | 5.47E-38 | -0.00552336 | 0.016235 | 0.241421 | 0.75 |
| rs116843064 | A | G | 0.019327 | 0.206178 | 0.0069176 | 651.961017 | 3.38E-195 | 0.0563202 | 0.05013 | 0.019543 | 0.27 |
| rs12575456 | A | G | 0.321829 | 0.044587 | 0.00203342 | 350.8310831 | 1.44E-106 | -0.00741112 | 0.014831 | 0.322621 | 0.62 |
| rs12740374 | T | G | 0.221008 | 0.028863 | 0.00229106 | 115.9026718 | 2.16E-36 | -0.0265625 | 0.016701 | 0.22161 | 0.11 |
| rs13235365 | T | C | 0.273616 | 0.025818 | 0.00214379 | 107.0580517 | 2.11E-33 | -0.0148186 | 0.015592 | 0.275625 | 0.36 |
| rs140584594 | G | A | 0.729761 | 0.031071 | 0.00212905 | 153.869474 | 3.07E-48 | -0.0239314 | 0.015557 | 0.730495 | 0.14 |
| rs144311893 | T | C | 0.022086 | 0.080962 | 0.0066916 | 114.4051934 | 1.07E-33 | -0.00272686 | 0.048636 | 0.022161 | 0.94 |
| rs150224153 | T | C | 0.029665 | -0.09347 | 0.00575879 | 203.2669313 | 3.06E-59 | -0.0124442 | 0.041559 | 0.030117 | 0.76 |
| rs150844304 | C | A | 0.025907 | -0.09054 | 0.00599215 | 167.2071999 | 1.39E-51 | 0.029213 | 0.044359 | 0.02513 | 0.54 |
| rs1601935 | T | G | 0.65541 | -0.09687 | 0.00201868 | 1719.464798 | 0 | 0.00283674 | 0.014709 | 0.655507 | 0.83 |
| rs17138358 | C | G | 0.398392 | -0.02723 | 0.00194811 | 143.5927331 | 2.18E-44 | 0.00668804 | 0.0142 | 0.397142 | 0.63 |
| rs174566 | G | A | 0.350061 | -0.05622 | 0.00199551 | 581.8813518 | 1.17E-174 | 0.0114281 | 0.014556 | 0.350478 | 0.43 |
| rs2066714 | C | T | 0.128622 | 0.04654 | 0.00284369 | 196.2121871 | 3.35E-60 | 0.00624308 | 0.020791 | 0.12748 | 0.77 |
| rs2256609 | G | A | 0.189238 | -0.03289 | 0.00244058 | 134.1131621 | 2.18E-41 | -0.020171 | 0.01773 | 0.188701 | 0.25 |
| rs2281718 | T | A | 0.612575 | 0.059643 | 0.00195409 | 683.2020777 | 1.32E-204 | -0.0272244 | 0.014274 | 0.61353 | 0.058 |
| rs2297409 | A | G | 0.194635 | -0.03336 | 0.00240571 | 140.9529247 | 1.02E-43 | 0.00694525 | 0.017534 | 0.194244 | 0.69 |
| rs2498786 | G | C | 0.615979 | -0.02547 | 0.00196826 | 124.0508839 | 2.59E-38 | -0.0168208 | 0.014309 | 0.616632 | 0.24 |
| rs2517951 | T | C | 0.677255 | 0.028238 | 0.00204079 | 140.8571697 | 1.53E-43 | 0.0158836 | 0.014847 | 0.677507 | 0.3 |
| rs2642438 | G | A | 0.702698 | 0.027656 | 0.00207854 | 129.1333178 | 2.15E-40 | 0.00544614 | 0.015179 | 0.702639 | 0.76 |
| rs2740488 | C | A | 0.265328 | -0.06865 | 0.00216181 | 743.5724274 | 2.58E-221 | 0.00706325 | 0.01575 | 0.265103 | 0.68 |
| rs2792751 | C | T | 0.725031 | -0.0361 | 0.0021336 | 210.0020612 | 3.22E-64 | 0.011639 | 0.015553 | 0.726024 | 0.45 |
| rs35493868 | G | C | 0.202024 | 0.037226 | 0.00237772 | 180.5644416 | 3.01E-55 | 0.0235216 | 0.01729 | 0.20293 | 0.16 |
| rs367070 | G | A | 0.225571 | 0.041795 | 0.00228709 | 246.6746468 | 1.33E-74 | 0.00371427 | 0.016619 | 0.227629 | 0.82 |
| rs3745683 | A | G | 0.074638 | -0.05464 | 0.00362928 | 166.6269949 | 3.25E-51 | -0.0132578 | 0.026409 | 0.074716 | 0.6 |
| rs3779788 | T | C | 0.144349 | 0.102233 | 0.00271342 | 1045.594514 | 0 | -0.0236628 | 0.019869 | 0.144172 | 0.23 |
| rs4803773 | G | A | 0.498722 | 0.040085 | 0.00199508 | 324.7799842 | 8.72E-90 | -0.0148197 | 0.014524 | 0.498515 | 0.3 |
| rs4871603 | T | C | 0.653471 | 0.036006 | 0.00199228 | 237.3127507 | 5.22E-73 | 0.00704529 | 0.014598 | 0.655033 | 0.64 |
| rs4969141 | T | C | 0.489676 | 0.029637 | 0.00191152 | 177.406984 | 3.23E-54 | -0.0021674 | 0.013904 | 0.49038 | 0.89 |
| rs55781197 | G | A | 0.115034 | 0.058735 | 0.00295144 | 283.9215489 | 4.04E-88 | 0.0134815 | 0.021735 | 0.115008 | 0.55 |
| rs559355 | T | A | 0.1573 | -0.03493 | 0.00261254 | 130.7117664 | 8.99E-41 | 0.032515 | 0.019077 | 0.156959 | 0.085 |
| rs571848809 | A | G | 0.105486 | -0.05692 | 0.00310573 | 247.100363 | 5.10E-75 | -0.0100843 | 0.022577 | 0.106396 | 0.66 |
| rs59781045 | T | C | 0.068117 | 0.074037 | 0.0037879 | 281.2967977 | 4.49E-85 | -0.0172666 | 0.027687 | 0.067811 | 0.55 |
| rs6073958 | C | T | 0.198804 | -0.06093 | 0.00239207 | 478.2849045 | 4.07E-143 | 0.00640605 | 0.017397 | 0.198748 | 0.69 |
| rs61352607 | T | G | 0.240851 | 0.030611 | 0.00222442 | 138.4618478 | 4.35E-43 | 0.0199849 | 0.016192 | 0.242358 | 0.24 |
| rs61805075 | A | G | 0.328694 | -0.02572 | 0.00202534 | 117.9428289 | 6.05E-37 | -0.0104528 | 0.014792 | 0.328265 | 0.49 |
| rs6469605 | T | C | 0.568501 | 0.03162 | 0.00191959 | 198.2382846 | 5.82E-61 | 0.0195369 | 0.014046 | 0.567836 | 0.16 |
| rs676210 | A | G | 0.205227 | 0.059269 | 0.00235237 | 463.4304475 | 4.47E-140 | 0.0390309 | 0.017191 | 0.20453 | 0.022 |
| rs686030 | A | C | 0.858823 | 0.049824 | 0.00273951 | 243.3077736 | 6.52E-74 | 0.0104749 | 0.019934 | 0.858493 | 0.61 |
| rs6902116 | G | A | 0.322025 | -0.02731 | 0.0020592 | 131.5679643 | 3.89E-40 | 0.0330709 | 0.014989 | 0.323253 | 0.029 |
| rs6989064 | T | C | 0.546975 | 0.063554 | 0.00190475 | 810.189707 | 4.30E-244 | 0.00587409 | 0.013942 | 0.546616 | 0.67 |
| rs7136506 | C | T | 0.2164 | -0.03611 | 0.00235185 | 178.6893041 | 3.38E-53 | 0.00163026 | 0.017153 | 0.215945 | 0.9 |
| rs7170361 | C | T | 0.285912 | 0.081242 | 0.00216071 | 1091.593707 | 0 | -0.0266748 | 0.015749 | 0.285619 | 0.09 |
| rs73607786 | A | G | 0.012812 | 0.099592 | 0.0084655 | 101.3720554 | 5.96E-32 | -0.0348462 | 0.06351 | 0.012206 | 0.6 |
| rs771481 | A | T | 0.18382 | 0.028815 | 0.0024568 | 100.6621203 | 9.10E-32 | 0.0276807 | 0.017932 | 0.183935 | 0.13 |
| rs77960347 | G | A | 0.013349 | 0.290966 | 0.00830188 | 902.847334 | 4.15E-269 | 0.0212548 | 0.060372 | 0.013383 | 0.73 |
| rs7817574 | C | T | 0.185003 | 0.033199 | 0.00243942 | 134.2962852 | 3.53E-42 | -0.00190999 | 0.017831 | 0.185659 | 0.93 |
| rs79600951 | G | C | 0.092057 | -0.1068 | 0.00325908 | 771.603106 | 1.66E-235 | -0.00779405 | 0.024001 | 0.092103 | 0.72 |
| rs8086351 | G | C | 0.824212 | 0.083959 | 0.0025068 | 826.7905632 | 6.22E-246 | 0.00182607 | 0.018222 | 0.823141 | 0.91 |
| rs921919 | A | G | 0.669162 | -0.04167 | 0.00206455 | 310.7214989 | 1.43E-90 | -0.0362883 | 0.015062 | 0.670681 | 0.017 |
| rs9296128 | A | G | 0.312474 | -0.02551 | 0.00205198 | 112.9433005 | 1.80E-35 | 0.00916565 | 0.014984 | 0.310734 | 0.52 |
| rs964184 | C | G | 0.866364 | 0.10544 | 0.00279456 | 1042.562386 | 0 | 0.0221587 | 0.020451 | 0.86722 | 0.27 |
| rs998584 | A | C | 0.482733 | -0.03419 | 0.00190975 | 235.9522068 | 1.12E-71 | -0.0135839 | 0.013921 | 0.482181 | 0.35 |
| rs9987289 | G | A | 0.908737 | 0.08729 | 0.00329366 | 511.164976 | 9.07E-155 | -0.0075221 | 0.024071 | 0.9084226 | 0.77 |
| rs9989419 | G | A | 0.605917 | 0.143765 | 0.00192633 | 4026.828073 | 0 | 0.00429374 | 0.014199 | 0.606051 | 0.79 |

Abbreviations: SE, standard error; EAF, effect allele frequency; SNP, single nucleotide polymorphism; FS, frozen shoulder.

Supplement table 7. Summary data of SNPs used in MR analysis of IVs associated with LDL-frozen shoulder.

| **SNP** | **Effect Allele** | **Other Allele** | **EAF** | **Beta Estimate** | **SE** | **F-statistic** | **P.vale** | **beta.FS** | **se. FS** | **eaf. FS** | **pval. FS** |
| --- | --- | --- | --- | --- | --- | --- | --- | --- | --- | --- | --- |
| rs10423733 | C | T | 0.180142 | -0.10331 | 0.002708 | 1393.114377 | 0 | -0.00672 | 0.018156 | 0.179813 | 0.7 |
| rs11206517 | G | T | 0.033149 | 0.068029 | 0.005806 | 130.7253793 | 1.05E-31 | 0.021017 | 0.03891 | 0.032907 | 0.57 |
| rs11591147 | T | G | 0.017468 | -0.34846 | 0.007931 | 1843.821775 | 0 | -0.02736 | 0.052775 | 0.017594 | 0.62 |
| rs1169294 | A | G | 0.310089 | 0.024504 | 0.002258 | 113.212638 | 1.99E-27 | 0.01204 | 0.015069 | 0.308528 | 0.42 |
| rs117733303 | G | A | 0.018422 | 0.083818 | 0.007738 | 111.9602226 | 2.45E-27 | 0.041066 | 0.051435 | 0.018539 | 0.44 |
| rs118039278 | A | G | 0.078672 | 0.083506 | 0.003885 | 445.7837022 | 1.83E-102 | 0.011387 | 0.025746 | 0.079697 | 0.67 |
| rs12208357 | T | C | 0.070102 | 0.056984 | 0.004096 | 186.5861245 | 5.39E-44 | 0.00157 | 0.027321 | 0.069981 | 0.97 |
| rs12611067 | T | G | 0.351293 | -0.03174 | 0.002174 | 202.330015 | 2.97E-48 | -0.0095 | 0.014566 | 0.351801 | 0.51 |
| rs12916 | C | T | 0.400537 | 0.062118 | 0.002127 | 817.8199832 | 1.75E-187 | -0.00627 | 0.014168 | 0.399962 | 0.66 |
| rs1367117 | A | G | 0.335193 | 0.082872 | 0.0022 | 1352.560753 | 0 | -0.02677 | 0.014674 | 0.337271 | 0.068 |
| rs1551891 | A | G | 0.088107 | -0.17363 | 0.00366 | 2144.480185 | 0 | -0.02532 | 0.024574 | 0.087782 | 0.3 |
| rs174564 | G | A | 0.348557 | -0.0319 | 0.00219 | 203.6521373 | 4.50E-48 | 0.010099 | 0.014565 | 0.349484 | 0.49 |
| rs183130 | T | C | 0.323808 | -0.03295 | 0.00223 | 209.6054 | 2.11E-49 | -0.01505 | 0.014841 | 0.324471 | 0.31 |
| rs1883711 | C | G | 0.031412 | 0.10277 | 0.006092 | 283.3126 | 7.49E-64 | -0.03364 | 0.040625 | 0.031292 | 0.41 |
| rs2073547 | G | A | 0.184007 | 0.03555 | 0.002673 | 167.255 | 2.31E-40 | -0.00618 | 0.017798 | 0.183931 | 0.72 |
| rs2618566 | T | G | 0.659861 | -0.02492 | 0.0022 | 122.8396 | 9.59E-30 | 0.004558 | 0.014657 | 0.661239 | 0.76 |
| rs2642438 | G | A | 0.702657 | 0.025322 | 0.002273 | 118.0663 | 8.02E-29 | 0.005446 | 0.015179 | 0.702639 | 0.76 |
| rs2740488 | C | A | 0.265466 | -0.02525 | 0.002365 | 109.5231 | 1.34E-26 | 0.007063 | 0.01575 | 0.265103 | 0.68 |
| rs28601761 | G | C | 0.418688 | -0.06201 | 0.002136 | 826.1582 | 2.93E-185 | 0.017212 | 0.014222 | 0.419573 | 0.25 |
| rs34042070 | G | C | 0.187927 | 0.048523 | 0.00268 | 316.8149 | 2.92E-73 | -0.02054 | 0.01789 | 0.186704 | 0.26 |
| rs35081008 | T | C | 0.14811 | -0.03191 | 0.002929 | 113.229 | 1.22E-27 | -0.02101 | 0.019679 | 0.147403 | 0.28 |
| rs36043200 | A | G | 0.516694 | -0.02659 | 0.002092 | 155.6222 | 5.06E-37 | -0.00785 | 0.013924 | 0.516042 | 0.56 |
| rs430096 | G | A | 0.221046 | -0.05439 | 0.002514 | 449.2434 | 8.63E-104 | 0.003673 | 0.016814 | 0.21981 | 0.84 |
| rs4307732 | A | G | 0.105876 | 0.044832 | 0.003399 | 167.7051497 | 9.81E-40 | -0.0108 | 0.022661 | 0.105475 | 0.64 |
| rs4452060 | A | C | 0.420697 | 0.083271 | 0.002104 | 1494.002 | 0 | 0.011382 | 0.014102 | 0.420404 | 0.4 |
| rs4665985 | C | A | 0.273129 | 0.024176 | 0.002359 | 102.2645 | 1.18E-24 | 0.001136 | 0.015765 | 0.271777 | 0.95 |
| rs472495 | T | G | 0.648959 | 0.042574 | 0.002181 | 364.1231 | 7.26E-85 | 0.000258 | 0.01457 | 0.650275 | 0.98 |
| rs4738684 | G | A | 0.664652 | -0.03154 | 0.002209 | 195.4937 | 3.03E-46 | 0.039206 | 0.014712 | 0.664301 | 0.0083 |
| rs4970834 | T | C | 0.186642 | -0.1053 | 0.002682 | 1488.184 | 0 | -0.01452 | 0.017878 | 0.187394 | 0.42 |
| rs556107 | T | C | 0.523338 | 0.035171 | 0.002088 | 272.0537 | 1.23E-63 | -0.01209 | 0.013943 | 0.522728 | 0.38 |
| rs56130071 | C | G | 0.216984 | 0.03318 | 0.002538 | 164.8647 | 4.67E-39 | -0.00093 | 0.016854 | 0.218339 | 0.96 |
| rs62116889 | C | T | 0.068292 | -0.04958 | 0.004112 | 137.8418 | 1.80E-33 | 0.015916 | 0.027651 | 0.067795 | 0.54 |
| rs6544713 | C | T | 0.676864 | -0.05372 | 0.002223 | 556.7355 | 5.04E-129 | 0.015632 | 0.014828 | 0.676597 | 0.3 |
| rs6680227 | A | G | 0.035237 | -0.07466 | 0.005633 | 167.0157 | 4.32E-40 | -0.03167 | 0.037766 | 0.03494 | 0.4 |
| rs6709904 | G | A | 0.112585 | -0.04344 | 0.003296 | 166.1843 | 1.15E-39 | -0.02107 | 0.022094 | 0.111535 | 0.33 |
| rs6874202 | C | T | 0.634269 | 0.032331 | 0.002163 | 213.7487 | 1.61E-50 | -0.01358 | 0.014408 | 0.634994 | 0.34 |
| rs71205205 | A | C | 0.11784 | 0.04546 | 0.003249 | 189.3656 | 1.73E-44 | 0.004072 | 0.021658 | 0.117736 | 0.85 |
| rs7202323 | G | T | 0.229741 | -0.02554 | 0.002477 | 101.6992 | 6.48E-25 | -0.00576 | 0.01652 | 0.228591 | 0.75 |
| rs7746081 | A | G | 0.304113 | -0.02347 | 0.002267 | 102.7574 | 4.02E-25 | 0.004358 | 0.015126 | 0.302741 | 0.81 |
| rs77542162 | G | A | 0.022495 | 0.128484 | 0.00704 | 320.0647 | 2.06E-74 | 0.010051 | 0.046495 | 0.022865 | 0.8 |
| rs79220007 | C | T | 0.076135 | -0.05734 | 0.003921 | 203.8441 | 2.04E-48 | -0.08459 | 0.025989 | 0.077172 | 0.0011 |
| rs964184 | C | G | 0.866409 | -0.05758 | 0.003064 | 338.4004 | 8.16E-79 | 0.022159 | 0.020451 | 0.86722 | 0.27 |
| rs9987289 | G | A | 0.908808 | 0.045382 | 0.003622 | 150.4428 | 5.07E-36 | -0.00752 | 0.024071 | 0.908423 | 0.77 |

Abbreviations: SE, standard error; EAF, effect allele frequency; SNP, single nucleotide polymorphism; FS, frozen shoulder.

Supplement table 8. Summary data of SNPs used in MR analysis of IVs associated with TG-frozen shoulder.

| **SNP** | **Effect Allele** | **Other Allele** | **EAF** | **Beta Estimate** | **SE** | **F-statistic** | **P.vale** | **beta.FS** | **se. FS** | **eaf. FS** | **pval. FS** |
| --- | --- | --- | --- | --- | --- | --- | --- | --- | --- | --- | --- |
| rs10773049 | C | T | 0.39535 | -0.0290398 | 0.002037 | 177.8814 | 4.07E-46 | -0.0093 | 0.014227 | 0.395267 | 0.5 |
| rs1077835 | G | A | 0.220459 | 0.0473668 | 0.002405 | 340.3551 | 2.20E-86 | -0.00439 | 0.016789 | 0.220062 | 0.78 |
| rs11122450 | G | T | 0.611733 | -0.0481745 | 0.002037 | 486.7301 | 1.29E-123 | -0.02758 | 0.014265 | 0.612569 | 0.055 |
| rs112259268 | A | C | 0.028291 | 0.139849 | 0.006014 | 474.7368 | 1.26E-119 | 0.017134 | 0.041747 | 0.028585 | 0.65 |
| rs116843064 | A | G | 0.019327 | -0.226505 | 0.00721 | 859.3539 | 1.26E-216 | 0.05632 | 0.05013 | 0.019543 | 0.27 |
| rs12446515 | T | C | 0.322889 | -0.0334207 | 0.002136 | 215.4958 | 3.47E-55 | -0.01571 | 0.0149 | 0.323643 | 0.3 |
| rs12546096 | G | A | 0.252369 | 0.0278972 | 0.00231 | 129.5551 | 1.39E-33 | -0.00242 | 0.016251 | 0.251077 | 0.86 |
| rs12928099 | A | C | 0.296442 | -0.0282116 | 0.002181 | 146.4609 | 2.92E-38 | -0.02016 | 0.015228 | 0.296033 | 0.19 |
| rs13108218 | G | A | 0.614859 | -0.0305169 | 0.002058 | 194.6032 | 9.71E-50 | -0.0124 | 0.014397 | 0.616668 | 0.38 |
| rs139974673 | C | T | 0.026131 | 0.143073 | 0.006239 | 459.9462 | 2.17E-116 | 0.013391 | 0.04411 | 0.025477 | 0.79 |
| rs150423652 | T | G | 0.006675 | 0.285824 | 0.012292 | 478.292 | 1.35E-119 | 0.003938 | 0.085987 | 0.006682 | 0.94 |
| rs150564454 | A | G | 0.011556 | -0.101751 | 0.009632 | 104.3331 | 4.37E-26 | 0.010324 | 0.068188 | 0.011381 | 0.87 |
| rs17184382 | C | A | 0.425367 | -0.0219251 | 0.00201 | 103.6626 | 1.08E-27 | -0.03363 | 0.014027 | 0.425943 | 0.018 |
| rs174566 | G | A | 0.34974 | 0.0485019 | 0.00208 | 472.3862 | 2.76E-120 | 0.011428 | 0.014556 | 0.350478 | 0.43 |
| rs17585887 | C | T | 0.591074 | -0.0285849 | 0.002017 | 174.2665 | 1.31E-45 | 0.018764 | 0.014102 | 0.590821 | 0.19 |
| rs1801689 | C | A | 0.030543 | -0.06613 | 0.005777 | 114.2437 | 2.44E-30 | -0.00993 | 0.040595 | 0.03014 | 0.8 |
| rs186696265 | T | C | 0.014693 | -0.104318 | 0.008285 | 139.0016 | 2.37E-36 | 0.089542 | 0.057697 | 0.014829 | 0.12 |
| rs188247550 | T | C | 0.013117 | -0.134286 | 0.009131 | 205.9903 | 5.89E-49 | -0.00659 | 0.063469 | 0.013253 | 0.92 |
| rs2043085 | C | T | 0.612184 | -0.0308028 | 0.002042 | 198.777 | 1.95E-51 | 0.006686 | 0.014252 | 0.612464 | 0.65 |
| rs2068888 | A | G | 0.450732 | -0.0318216 | 0.001996 | 221.2315 | 3.16E-57 | -0.01148 | 0.013946 | 0.450073 | 0.41 |
| rs2081687 | C | T | 0.663201 | -0.0261379 | 0.002091 | 134.6394 | 7.59E-36 | 0.040425 | 0.014688 | 0.662932 | 0.0064 |
| rs2130382 | G | C | 0.313212 | -0.0251437 | 0.002145 | 119.9831 | 9.65E-32 | 0.017315 | 0.014988 | 0.312349 | 0.26 |
| rs2240466 | A | G | 0.122916 | -0.122769 | 0.003018 | 1437.881 | 0 | 0.010461 | 0.02108 | 0.123298 | 0.59 |
| rs2302263 | T | C | 0.088863 | 0.0436112 | 0.003491 | 135.8679 | 8.03E-36 | 0.037202 | 0.024492 | 0.088489 | 0.13 |
| rs28383314 | C | T | 0.624109 | 0.0379005 | 0.002047 | 297.4318 | 1.48E-76 | 0.012213 | 0.014316 | 0.624114 | 0.39 |
| rs28752924 | C | T | 0.445662 | 0.0265556 | 0.002057 | 153.7185 | 3.96E-38 | -0.00192 | 0.014419 | 0.44304 | 0.89 |
| rs308 | G | T | 0.020597 | -0.159413 | 0.006984 | 452.6273 | 2.64E-115 | -0.00204 | 0.048951 | 0.020664 | 0.98 |
| rs326222 | C | T | 0.697957 | 0.0252203 | 0.002157 | 118.3033 | 1.39E-31 | 0.002986 | 0.015098 | 0.697465 | 0.84 |
| rs343 | A | C | 0.083129 | -0.141493 | 0.003611 | 1350.021 | 0 | 0.002889 | 0.025414 | 0.082651 | 0.92 |
| rs3775228 | T | C | 0.399717 | 0.0338197 | 0.002036 | 242.1971 | 5.83E-62 | 0.006296 | 0.014222 | 0.399709 | 0.67 |
| rs4760254 | C | G | 0.23908 | -0.0281414 | 0.002325 | 127.1104 | 1.00E-33 | 0.014267 | 0.016206 | 0.24087 | 0.41 |
| rs480823 | C | T | 0.078996 | 0.15572 | 0.00372 | 1561.611 | 0 | -0.0417 | 0.026079 | 0.078825 | 0.11 |
| rs4841580 | C | T | 0.434941 | -0.0245383 | 0.002001 | 130.5642 | 1.42E-34 | -0.00382 | 0.014058 | 0.434589 | 0.79 |
| rs6073958 | C | T | 0.198674 | 0.0556952 | 0.002493 | 436.0109 | 1.63E-110 | 0.006406 | 0.017397 | 0.198748 | 0.69 |
| rs61905078 | C | A | 0.073841 | 0.199898 | 0.003795 | 2423.609 | 0 | -0.02477 | 0.026589 | 0.073746 | 0.33 |
| rs676210 | A | G | 0.205293 | -0.0735169 | 0.00245 | 779.1203 | 7.09E-198 | 0.039031 | 0.017191 | 0.20453 | 0.022 |
| rs6800707 | G | C | 0.810543 | 0.0298848 | 0.002538 | 121.001 | 5.35E-32 | 0.012807 | 0.017705 | 0.809978 | 0.46 |
| rs684773 | C | A | 0.766797 | 0.0291133 | 0.002347 | 133.7246 | 2.50E-35 | 0.032086 | 0.016405 | 0.767276 | 0.049 |
| rs6882076 | C | T | 0.634285 | 0.0331011 | 0.002061 | 224.2928 | 4.95E-58 | -0.01374 | 0.014407 | 0.634988 | 0.34 |
| rs6999569 | G | A | 0.470737 | -0.0860885 | 0.001981 | 156.9991 | 0 | 0.010856 | 0.013905 | 0.472623 | 0.46 |
| rs7000494 | C | G | 0.030042 | 0.136538 | 0.005842 | 1634.667 | 7.96E-121 | 0.004985 | 0.041045 | 0.030001 | 0.88 |
| rs7140110 | C | T | 0.298338 | 0.0283172 | 0.002178 | 479.6708 | 1.21E-38 | -0.00214 | 0.015203 | 0.297462 | 0.89 |
| rs72555385 | G | A | 0.048975 | 0.0653776 | 0.004618 | 148.1035 | 1.67E-45 | -0.01562 | 0.032362 | 0.048691 | 0.65 |
| rs73243877 | G | A | 0.168135 | 0.0288117 | 0.002656 | 175.6627 | 2.00E-27 | 0.018567 | 0.018568 | 0.167834 | 0.3 |
| rs75609851 | A | G | 0.01048 | -0.199116 | 0.01004 | 102.4311 | 1.57E-87 | 0.052252 | 0.070508 | 0.01047 | 0.46 |
| rs77009508 | G | A | 0.073734 | 0.0450912 | 0.0038 | 362.9425 | 1.77E-32 | -0.00721 | 0.026446 | 0.074651 | 0.8 |
| rs7786102 | A | G | 0.715703 | -0.0283695 | 0.002201 | 122.5152 | 5.12E-38 | 0.01125 | 0.015411 | 0.717196 | 0.48 |
| rs78484485 | A | G | 0.054016 | -0.0758529 | 0.004399 | 259.4707 | 1.23E-66 | 0.030116 | 0.030908 | 0.053638 | 0.32 |
| rs9436661 | G | T | 0.352919 | -0.0777322 | 0.002078 | 1220.445 | 2.84E-306 | -0.02498 | 0.014542 | 0.353911 | 0.09 |
| rs998584 | A | C | 0.482727 | 0.0401182 | 0.00199 | 354.761 | 2.20E-90 | -0.01358 | 0.013921 | 0.482181 | 0.35 |

Supplement table 9. Summary data of SNPs used in MR analysis of IVs associated with HDL-frozen shoulder in females.

| **SNP** | **Effect Allele** | **Other Allele** | **EAF** | **Beta Estimate** | **SE** | **F-statistic** | **P value** | **beta.FS** | **se.FS** | **eaf.FS** | **Pval.FS** |
| --- | --- | --- | --- | --- | --- | --- | --- | --- | --- | --- | --- |
| rs10162642 | A | G | 0.211684 | -0.0479223 | 0.0023419 | 309.8450868 | 4.60E-93 | -0.000125168 | 0.000248609 | 0.211822 | 0.614631 |
| rs1054852 | G | A | 0.376936 | 0.0351047 | 0.0020654 | 233.9538527 | 8.71E-65 | 0.000426701 | 0.000219876 | 0.373944 | 0.0523025 |
| rs11045171 | G | A | 0.197605 | 0.0283661 | 0.00240558 | 103.0965574 | 4.30E-32 | -4.26E-05 | 0.000255297 | 0.197654 | 0.867567 |
| rs112259268 | A | C | 0.028404 | -0.168534 | 0.00575922 | 634.2633255 | 3.04E-188 | -0.000214871 | 0.000604598 | 0.0289509 | 0.722294 |
| rs11239536 | A | T | 0.240967 | 0.0287501 | 0.00223129 | 122.1732744 | 5.47E-38 | 0.000124555 | 0.000236532 | 0.241874 | 0.598481 |
| rs114165349 | C | G | 0.023124 | -0.0810512 | 0.00636562 | 119.9218018 | 3.89E-37 | -3.95E-05 | 0.000670878 | 0.0236515 | 0.953042 |
| rs116843064 | A | G | 0.019327 | 0.206178 | 0.0069176 | 651.961017 | 3.38E-195 | -0.000321842 | 0.000737462 | 0.0191606 | 0.662533 |
| rs12575456 | A | G | 0.321829 | 0.0445866 | 0.00203342 | 350.8310831 | 1.44E-106 | 0.00016904 | 0.000215968 | 0.323951 | 0.433799 |
| rs12740374 | T | G | 0.221008 | 0.0288629 | 0.00229106 | 115.9026718 | 2.16E-36 | -0.000409038 | 0.000243662 | 0.221644 | 0.0932101 |
| rs13235365 | T | C | 0.273616 | 0.0258181 | 0.00214379 | 107.0580517 | 2.11E-33 | -0.000323462 | 0.000226822 | 0.277844 | 0.153853 |
| rs140584594 | G | A | 0.729761 | 0.031071 | 0.00212905 | 153.869474 | 3.07E-48 | -0.00021191 | 0.000226543 | 0.730507 | 0.349579 |
| rs141469619 | G | A | 0.009879 | -0.203479 | 0.0101857 | 327.446451 | 8.74E-89 | -0.000681979 | 0.00106972 | 0.0101031 | 0.523779 |
| rs144311893 | T | C | 0.022086 | 0.0809615 | 0.0066916 | 114.4051934 | 1.07E-33 | -0.000938543 | 0.000706738 | 0.0222688 | 0.184182 |
| rs150224153 | T | C | 0.029665 | -0.0934689 | 0.00575879 | 203.2669313 | 3.06E-59 | 0.000532405 | 0.000604526 | 0.0302722 | 0.378482 |
| rs150844304 | C | A | 0.025907 | -0.090543 | 0.00599215 | 167.2071999 | 1.39E-51 | 0.000178771 | 0.000654346 | 0.0245613 | 0.784696 |
| rs1601935 | T | G | 0.65541 | -0.0968707 | 0.00201868 | 1719.464798 | 0 | -0.00025819 | 0.000214384 | 0.6565 | 0.228462 |
| rs17138358 | C | G | 0.398392 | -0.0272272 | 0.00194811 | 143.5927331 | 2.18E-44 | -0.000140532 | 0.000207276 | 0.395861 | 0.497775 |
| rs174566 | G | A | 0.350061 | -0.0562241 | 0.00199551 | 581.8813518 | 1.17E-174 | 0.000341201 | 0.000212372 | 0.349943 | 0.10814 |
| rs2066714 | C | T | 0.128622 | 0.0465396 | 0.00284369 | 196.2121871 | 3.35E-60 | -5.66E-05 | 0.000303441 | 0.127007 | 0.852011 |
| rs2256609 | G | A | 0.189238 | -0.0328881 | 0.00244058 | 134.1131621 | 2.18E-41 | 2.72E-05 | 0.000257999 | 0.189662 | 0.915978 |
| rs2281718 | T | A | 0.612575 | 0.0596431 | 0.00195409 | 683.2020777 | 1.32E-204 | -2.75E-05 | 0.000208136 | 0.614282 | 0.894821 |
| rs2297409 | A | G | 0.194635 | -0.0333565 | 0.00240571 | 140.9529247 | 1.02E-43 | 5.09E-05 | 0.000255721 | 0.194218 | 0.842353 |
| rs2498786 | G | C | 0.615979 | -0.0254741 | 0.00196826 | 124.0508839 | 2.59E-38 | 0.000264103 | 0.000208772 | 0.617069 | 0.205862 |
| rs2517951 | T | C | 0.677255 | 0.028238 | 0.00204079 | 140.8571697 | 1.53E-43 | 0.000383087 | 0.000216739 | 0.678963 | 0.0771462 |
| rs2642438 | G | A | 0.702698 | 0.0276562 | 0.00207854 | 129.1333178 | 2.15E-40 | 0.000140963 | 0.000221213 | 0.703454 | 0.523977 |
| rs2740488 | C | A | 0.265328 | -0.0686515 | 0.00216181 | 743.5724274 | 2.58E-221 | 0.00034378 | 0.000229711 | 0.264895 | 0.134506 |
| rs2792751 | C | T | 0.725031 | -0.0360998 | 0.0021336 | 210.0020612 | 3.22E-64 | 0.000415433 | 0.000226875 | 0.726135 | 0.0670863 |
| rs35493868 | G | C | 0.202024 | 0.0372262 | 0.00237772 | 180.5644416 | 3.01E-55 | 0.00043159 | 0.000251254 | 0.204181 | 0.0858444 |
| rs367070 | G | A | 0.225571 | 0.0417949 | 0.00228709 | 246.6746468 | 1.33E-74 | -2.96E-05 | 0.000241366 | 0.229843 | 0.902479 |
| rs3745683 | A | G | 0.074638 | -0.0546353 | 0.00362928 | 166.6269949 | 3.25E-51 | -1.39E-05 | 0.000386451 | 0.073926 | 0.971233 |
| rs3779788 | T | C | 0.144349 | 0.102233 | 0.00271342 | 1045.594514 | 0 | -0.000541992 | 0.000289408 | 0.144216 | 0.061103 |
| rs4803773 | G | A | 0.498722 | 0.0400846 | 0.00199508 | 324.7799842 | 8.72E-90 | -5.21E-05 | 0.00021142 | 0.497872 | 0.805443 |
| rs4871603 | T | C | 0.653471 | 0.0360061 | 0.00199228 | 237.3127507 | 5.22E-73 | -8.14E-05 | 0.000213202 | 0.657201 | 0.702659 |
| rs4969141 | T | C | 0.489676 | 0.0296373 | 0.00191152 | 177.406984 | 3.23E-54 | -0.000120023 | 0.000202592 | 0.489456 | 0.55356 |
| rs55781197 | G | A | 0.115034 | 0.0587349 | 0.00295144 | 283.9215489 | 4.04E-88 | -6.20E-05 | 0.000317594 | 0.114195 | 0.845201 |
| rs559355 | T | A | 0.1573 | -0.0349311 | 0.00261254 | 130.7117664 | 8.99E-41 | 0.000471903 | 0.000277986 | 0.156745 | 0.0895891 |
| rs571848809 | A | G | 0.105486 | -0.0569166 | 0.00310573 | 247.100363 | 5.10E-75 | -0.000236962 | 0.000329639 | 0.105957 | 0.472232 |
| rs59781045 | T | C | 0.068117 | 0.074037 | 0.0037879 | 281.2967977 | 4.49E-85 | -1.36E-05 | 0.000404416 | 0.0675444 | 0.973205 |
| rs6073958 | C | T | 0.198804 | -0.0609299 | 0.00239207 | 478.2849045 | 4.07E-143 | 7.77E-08 | 0.000253779 | 0.198407 | 0.999756 |
| rs61352607 | T | G | 0.240851 | 0.0306111 | 0.00222442 | 138.4618478 | 4.35E-43 | -0.000305896 | 0.000235439 | 0.243996 | 0.193857 |
| rs61805075 | A | G | 0.328694 | -0.0257183 | 0.00202534 | 117.9428289 | 6.05E-37 | -0.00027151 | 0.00021553 | 0.328602 | 0.207768 |
| rs6469605 | T | C | 0.568501 | 0.0316197 | 0.00191959 | 198.2382846 | 5.82E-61 | 0.000152608 | 0.000204812 | 0.568975 | 0.456203 |
| rs676210 | A | G | 0.205227 | 0.0592694 | 0.00235237 | 463.4304475 | 4.47E-140 | 0.000110685 | 0.000250999 | 0.203985 | 0.659231 |
| rs686030 | A | C | 0.858823 | 0.0498241 | 0.00273951 | 243.3077736 | 6.52E-74 | 0.000161501 | 0.000290995 | 0.859252 | 0.578898 |
| rs6902116 | G | A | 0.322025 | -0.0273073 | 0.0020592 | 131.5679643 | 3.89E-40 | 0.000409576 | 0.00021847 | 0.324036 | 0.0608292 |
| rs6989064 | T | C | 0.546975 | 0.0635535 | 0.00190475 | 810.189707 | 4.30E-244 | 0.000190174 | 0.000202904 | 0.545746 | 0.348625 |
| rs7136506 | C | T | 0.2164 | -0.036108 | 0.00235185 | 178.6893041 | 3.38E-53 | 0.00021221 | 0.000250096 | 0.215916 | 0.396154 |
| rs7170361 | C | T | 0.285912 | 0.0812416 | 0.00216071 | 1091.593707 | 0 | -0.000391905 | 0.000229688 | 0.285081 | 0.0879637 |
| rs73607786 | A | G | 0.012812 | 0.0995915 | 0.0084655 | 101.3720554 | 5.96E-32 | 0.00154717 | 0.000938295 | 0.0118818 | 0.0991674 |
| rs771481 | A | T | 0.18382 | 0.0288148 | 0.0024568 | 100.6621203 | 9.10E-32 | 0.000548421 | 0.000261271 | 0.184052 | 0.0358136 |
| rs77960347 | G | A | 0.013349 | 0.290966 | 0.00830188 | 902.847334 | 4.15E-269 | -0.000535209 | 0.000878809 | 0.0134132 | 0.542514 |
| rs7817574 | C | T | 0.185003 | 0.0331985 | 0.00243942 | 134.2962852 | 3.53E-42 | -0.000601041 | 0.000260398 | 0.184479 | 0.0209911 |
| rs79600951 | G | C | 0.092057 | -0.106795 | 0.00325908 | 771.603106 | 1.66E-235 | -6.33E-05 | 0.000350189 | 0.091721 | 0.856502 |
| rs8086351 | G | C | 0.824212 | 0.0839587 | 0.0025068 | 826.7905632 | 6.22E-246 | 0.000458991 | 0.000265037 | 0.822179 | 0.0833105 |
| rs921919 | A | G | 0.669162 | -0.041665 | 0.00206455 | 310.7214989 | 1.43E-90 | -0.000385755 | 0.000219597 | 0.671097 | 0.0789789 |
| rs9296128 | A | G | 0.312474 | -0.0255061 | 0.00205198 | 112.9433005 | 1.80E-35 | 0.000250814 | 0.000219258 | 0.308399 | 0.252658 |
| rs964184 | C | G | 0.866364 | 0.10544 | 0.00279456 | 1042.562386 | 0 | 7.38E-05 | 0.000298113 | 0.867459 | 0.804348 |
| rs998584 | A | C | 0.482733 | -0.0341901 | 0.00190975 | 235.9522068 | 1.12E-71 | -0.000162248 | 0.000203107 | 0.482308 | 0.424391 |
| rs9987289 | G | A | 0.908737 | 0.0872902 | 0.00329366 | 511.164976 | 9.07E-155 | -0.000136008 | 0.000349048 | 0.9076833 | 0.696793 |
| rs9989419 | G | A | 0.605917 | 0.143765 | 0.00192633 | 4026.828073 | 0 | 0.00020488 | 0.000206713 | 0.606598 | 0.321622 |

Abbreviations: SE, standard error; EAF, effect allele frequency; SNP, single nucleotide polymorphism; FS, frozen shoulder.

Supplement table 10. Summary data of SNPs used in MR analysis of IVs associated with HDL-frozen shoulder in males.

| **SNP** | **Effect Allele** | **Other Allele** | **EAF** | **Beta Estimate** | **SE** | **F-statistic** | **P value** | **beta.FS** | **se.FS** | **eaf.FS** | **Pval.FS** |
| --- | --- | --- | --- | --- | --- | --- | --- | --- | --- | --- | --- |
| rs10162642 | A | G | 0.211684 | -0.0479223 | 0.0023419 | 309.8450868 | 4.60E-93 | -5.62E-05 | 0.000214454 | 0.210986 | 0.793415 |
| rs1054852 | G | A | 0.376936 | 0.0351047 | 0.0020654 | 233.9538527 | 8.71E-65 | 4.69E-05 | 0.000188843 | 0.376158 | 0.803662 |
| rs11045171 | G | A | 0.197605 | 0.0283661 | 0.00240558 | 103.0965574 | 4.30E-32 | 4.83E-05 | 0.000219979 | 0.197806 | 0.826102 |
| rs112259268 | A | C | 0.028404 | -0.168534 | 0.00575922 | 634.2633255 | 3.04E-188 | 0.000408259 | 0.00052315 | 0.0286919 | 0.435165 |
| rs11239536 | A | T | 0.240967 | 0.0287501 | 0.00223129 | 122.1732744 | 5.47E-38 | 0.000191971 | 0.000204293 | 0.240082 | 0.347381 |
| rs114165349 | C | G | 0.023124 | -0.0810512 | 0.00636562 | 119.9218018 | 3.89E-37 | -0.000895017 | 0.000581497 | 0.0232534 | 0.123767 |
| rs116843064 | A | G | 0.019327 | 0.206178 | 0.0069176 | 651.961017 | 3.38E-195 | 0.000487045 | 0.00061996 | 0.0200665 | 0.432099 |
| rs12575456 | A | G | 0.321829 | 0.0445866 | 0.00203342 | 350.8310831 | 1.44E-106 | -5.43E-05 | 0.000185902 | 0.323581 | 0.770353 |
| rs12740374 | T | G | 0.221008 | 0.0288629 | 0.00229106 | 115.9026718 | 2.16E-36 | -0.000303819 | 0.000209196 | 0.222 | 0.146415 |
| rs13235365 | T | C | 0.273616 | 0.0258181 | 0.00214379 | 107.0580517 | 2.11E-33 | -0.00014699 | 0.000195404 | 0.27634 | 0.45191 |
| rs140584594 | G | A | 0.729761 | 0.031071 | 0.00212905 | 153.869474 | 3.07E-48 | -0.00042129 | 0.000195136 | 0.731209 | 0.030855 |
| rs141469619 | G | A | 0.009879 | -0.203479 | 0.0101857 | 327.446451 | 8.74E-89 | -0.00032337 | 0.000913926 | 0.0102061 | 0.72347 |
| rs144311893 | T | C | 0.022086 | 0.0809615 | 0.0066916 | 114.4051934 | 1.07E-33 | 0.00139151 | 0.000608692 | 0.022262 | 0.0222516 |
| rs150224153 | T | C | 0.029665 | -0.0934689 | 0.00575879 | 203.2669313 | 3.06E-59 | -0.000370621 | 0.000519547 | 0.0303425 | 0.475628 |
| rs150844304 | C | A | 0.025907 | -0.090543 | 0.00599215 | 167.2071999 | 1.39E-51 | 0.000504806 | 0.000564284 | 0.0244856 | 0.371005 |
| rs1601935 | T | G | 0.65541 | -0.0968707 | 0.00201868 | 1719.464798 | 0 | 6.81E-05 | 0.000184361 | 0.654682 | 0.711708 |
| rs17138358 | C | G | 0.398392 | -0.0272272 | 0.00194811 | 143.5927331 | 2.18E-44 | -8.29E-05 | 0.000178165 | 0.396749 | 0.641694 |
| rs174566 | G | A | 0.350061 | -0.0562241 | 0.00199551 | 581.8813518 | 1.17E-174 | -1.14E-05 | 0.000182401 | 0.351636 | 0.950079 |
| rs2066714 | C | T | 0.128622 | 0.0465396 | 0.00284369 | 196.2121871 | 3.35E-60 | 0.000133076 | 0.000260577 | 0.127449 | 0.609565 |
| rs2256609 | G | A | 0.189238 | -0.0328881 | 0.00244058 | 134.1131621 | 2.18E-41 | -0.000410764 | 0.000222528 | 0.188168 | 0.0649095 |
| rs2281718 | T | A | 0.612575 | 0.0596431 | 0.00195409 | 683.2020777 | 1.32E-204 | 0.00010226 | 0.000178925 | 0.613721 | 0.567646 |
| rs2297409 | A | G | 0.194635 | -0.0333565 | 0.00240571 | 140.9529247 | 1.02E-43 | -0.000271331 | 0.00021974 | 0.194546 | 0.216912 |
| rs2498786 | G | C | 0.615979 | -0.0254741 | 0.00196826 | 124.0508839 | 2.59E-38 | -0.00022221 | 0.000179477 | 0.617041 | 0.215681 |
| rs2517951 | T | C | 0.677255 | 0.028238 | 0.00204079 | 140.8571697 | 1.53E-43 | 3.69E-05 | 0.000186547 | 0.678195 | 0.843383 |
| rs2642438 | G | A | 0.702698 | 0.0276562 | 0.00207854 | 129.1333178 | 2.15E-40 | -0.000347542 | 0.000190188 | 0.701347 | 0.0676472 |
| rs2740488 | C | A | 0.265328 | -0.0686515 | 0.00216181 | 743.5724274 | 2.58E-221 | 0.000228638 | 0.000197653 | 0.266174 | 0.24737 |
| rs2792751 | C | T | 0.725031 | -0.0360998 | 0.0021336 | 210.0020612 | 3.22E-64 | -0.000122862 | 0.000195576 | 0.727308 | 0.529868 |
| rs35493868 | G | C | 0.202024 | 0.0372262 | 0.00237772 | 180.5644416 | 3.01E-55 | -0.00018973 | 0.000216658 | 0.204028 | 0.381186 |
| rs367070 | G | A | 0.225571 | 0.0417949 | 0.00228709 | 246.6746468 | 1.33E-74 | 4.25E-06 | 0.000208448 | 0.228651 | 0.98374 |
| rs3745683 | A | G | 0.074638 | -0.0546353 | 0.00362928 | 166.6269949 | 3.25E-51 | 1.32E-05 | 0.000329835 | 0.07558 | 0.968089 |
| rs3779788 | T | C | 0.144349 | 0.102233 | 0.00271342 | 1045.594514 | 0 | -0.000136734 | 0.000249517 | 0.143729 | 0.583697 |
| rs4803773 | G | A | 0.498722 | 0.0400846 | 0.00199508 | 324.7799842 | 8.72E-90 | -7.54E-05 | 0.000182559 | 0.499236 | 0.679715 |
| rs4871603 | T | C | 0.653471 | 0.0360061 | 0.00199228 | 237.3127507 | 5.22E-73 | 0.000109726 | 0.000183226 | 0.655023 | 0.549269 |
| rs4969141 | T | C | 0.489676 | 0.0296373 | 0.00191152 | 177.406984 | 3.23E-54 | 0.000233436 | 0.000174452 | 0.490843 | 0.180862 |
| rs55781197 | G | A | 0.115034 | 0.0587349 | 0.00295144 | 283.9215489 | 4.04E-88 | 0.000125825 | 0.000272547 | 0.115331 | 0.644322 |
| rs559355 | T | A | 0.1573 | -0.0349311 | 0.00261254 | 130.7117664 | 8.99E-41 | -3.65E-05 | 0.000240647 | 0.155692 | 0.879604 |
| rs571848809 | A | G | 0.105486 | -0.0569166 | 0.00310573 | 247.100363 | 5.10E-75 | -5.81E-06 | 0.000282154 | 0.107313 | 0.983565 |
| rs59781045 | T | C | 0.068117 | 0.074037 | 0.0037879 | 281.2967977 | 4.49E-85 | -4.32E-05 | 0.000347357 | 0.067574 | 0.901005 |
| rs6073958 | C | T | 0.198804 | -0.0609299 | 0.00239207 | 478.2849045 | 4.07E-143 | -0.000170403 | 0.000218382 | 0.198788 | 0.435217 |
| rs61352607 | T | G | 0.240851 | 0.0306111 | 0.00222442 | 138.4618478 | 4.35E-43 | 6.44E-05 | 0.000203421 | 0.242447 | 0.751742 |
| rs61805075 | A | G | 0.328694 | -0.0257183 | 0.00202534 | 117.9428289 | 6.05E-37 | -5.34E-05 | 0.000185547 | 0.327682 | 0.773444 |
| rs6469605 | T | C | 0.568501 | 0.0316197 | 0.00191959 | 198.2382846 | 5.82E-61 | -0.000263768 | 0.000176212 | 0.565799 | 0.134426 |
| rs676210 | A | G | 0.205227 | 0.0592694 | 0.00235237 | 463.4304475 | 4.47E-140 | -0.000209221 | 0.000215957 | 0.204431 | 0.332643 |
| rs686030 | A | C | 0.858823 | 0.0498241 | 0.00273951 | 243.3077736 | 6.52E-74 | -0.000206153 | 0.000249843 | 0.857818 | 0.4093 |
| rs6902116 | G | A | 0.322025 | -0.0273073 | 0.0020592 | 131.5679643 | 3.89E-40 | 0.000303231 | 0.000187475 | 0.325649 | 0.105784 |
| rs6989064 | T | C | 0.546975 | 0.0635535 | 0.00190475 | 810.189707 | 4.30E-244 | -0.000206802 | 0.000175068 | 0.545915 | 0.2375 |
| rs7136506 | C | T | 0.2164 | -0.036108 | 0.00235185 | 178.6893041 | 3.38E-53 | -0.000496131 | 0.000214527 | 0.216868 | 0.0207418 |
| rs7170361 | C | T | 0.285912 | 0.0812416 | 0.00216071 | 1091.593707 | 0 | 0.00021256 | 0.000197667 | 0.286325 | 0.282222 |
| rs73607786 | A | G | 0.012812 | 0.0995915 | 0.0084655 | 101.3720554 | 5.96E-32 | -0.0011814 | 0.000798108 | 0.0121602 | 0.13881 |
| rs771481 | A | T | 0.18382 | 0.0288148 | 0.0024568 | 100.6621203 | 9.10E-32 | -0.000297656 | 0.000224936 | 0.18414 | 0.18574 |
| rs77960347 | G | A | 0.013349 | 0.290966 | 0.00830188 | 902.847334 | 4.15E-269 | -0.000792292 | 0.000749467 | 0.0136421 | 0.290449 |
| rs7817574 | C | T | 0.185003 | 0.0331985 | 0.00243942 | 134.2962852 | 3.53E-42 | 0.000117536 | 0.000223437 | 0.186418 | 0.598865 |
| rs79600951 | G | C | 0.092057 | -0.106795 | 0.00325908 | 771.603106 | 1.66E-235 | 0.000104659 | 0.000301496 | 0.0921169 | 0.728492 |
| rs8086351 | G | C | 0.824212 | 0.0839587 | 0.0025068 | 826.7905632 | 6.22E-246 | -0.000129135 | 0.000228627 | 0.82236 | 0.572192 |
| rs921919 | A | G | 0.669162 | -0.041665 | 0.00206455 | 310.7214989 | 1.43E-90 | -0.000335615 | 0.000188937 | 0.671535 | 0.0756808 |
| rs9296128 | A | G | 0.312474 | -0.0255061 | 0.00205198 | 112.9433005 | 1.80E-35 | 0.00013707 | 0.000188245 | 0.311331 | 0.466525 |
| rs964184 | C | G | 0.866364 | 0.10544 | 0.00279456 | 1042.562386 | 0 | 0.000120371 | 0.000257383 | 0.868234 | 0.640018 |
| rs998584 | A | C | 0.482733 | -0.0341901 | 0.00190975 | 235.9522068 | 1.12E-71 | -0.000276028 | 0.000174604 | 0.481018 | 0.113907 |
| rs9987289 | G | A | 0.908737 | 0.0872902 | 0.00329366 | 511.164976 | 9.07E-155 | -0.000462694 | 0.000302608 | 0.9088223 | 0.126261 |
| rs9989419 | G | A | 0.605917 | 0.143765 | 0.00192633 | 4026.828073 | 0 | -9.76E-05 | 0.000178318 | 0.605649 | 0.584026 |

Abbreviations: SE, standard error; EAF, effect allele frequency; SNP, single nucleotide polymorphism; FS, frozen shoulder.

Supplement table 11. Summary data of SNPs used in MR analysis of IVs associated with LDL-frozen shoulder in females.

| **SNP** | **Effect Allele** | **Other Allele** | **EAF** | **Beta Estimate** | **SE** | **F-statistic** | **P.vale** | **beta.FS** | **se. FS** | **eaf. FS** | **pval. FS** |
| --- | --- | --- | --- | --- | --- | --- | --- | --- | --- | --- | --- |
| rs10423733 | C | T | 0.180142 | -0.10331 | 0.002708 | 1393.114377 | 0 | -0.00013 | 0.000265 | 0.17989 | 0.614378 |
| rs11206517 | G | T | 0.033149 | 0.068029 | 0.005806 | 130.7253793 | 1.05E-31 | -0.00078 | 0.000568 | 0.032824 | 0.167587 |
| rs11591147 | T | G | 0.017468 | -0.34846 | 0.007931 | 1843.821775 | 0 | -0.0001 | 0.000768 | 0.017667 | 0.893467 |
| rs1169294 | A | G | 0.310089 | 0.024504 | 0.002258 | 113.212638 | 1.99E-27 | 0.000107 | 0.00022 | 0.307245 | 0.62555 |
| rs117733303 | G | A | 0.018422 | 0.083818 | 0.007738 | 111.9602226 | 2.45E-27 | -0.00017 | 0.000747 | 0.018741 | 0.821777 |
| rs118039278 | A | G | 0.078672 | 0.083506 | 0.003885 | 445.7837022 | 1.83E-102 | 0.000329 | 0.000373 | 0.080745 | 0.378091 |
| rs12208357 | T | C | 0.070102 | 0.056984 | 0.004096 | 186.5861245 | 5.39E-44 | -0.00036 | 0.000397 | 0.070194 | 0.361354 |
| rs12611067 | T | G | 0.351293 | -0.03174 | 0.002174 | 202.330015 | 2.97E-48 | -0.00013 | 0.000212 | 0.352018 | 0.540212 |
| rs12916 | C | T | 0.400537 | 0.062118 | 0.002127 | 817.8199832 | 1.75E-187 | 0.000305 | 0.000207 | 0.399456 | 0.140002 |
| rs1367117 | A | G | 0.335193 | 0.082872 | 0.0022 | 1352.560753 | 0 | -0.00015 | 0.000213 | 0.339252 | 0.471752 |
| rs1551891 | A | G | 0.088107 | -0.17363 | 0.00366 | 2144.480185 | 0 | -0.00039 | 0.000358 | 0.088227 | 0.272418 |
| rs174564 | G | A | 0.348557 | -0.0319 | 0.00219 | 203.6521373 | 4.50E-48 | 0.000346 | 0.000213 | 0.348915 | 0.103466 |
| rs183130 | T | C | 0.323808 | -0.03295 | 0.00223 | 209.6053591 | 2.11E-49 | 0.000164 | 0.000216 | 0.324813 | 0.447947 |
| rs1883711 | C | G | 0.031412 | 0.10277 | 0.006092 | 283.3125912 | 7.49E-64 | -1.85E-06 | 0.000592 | 0.031159 | 0.997503 |
| rs2618566 | T | G | 0.659861 | -0.02492 | 0.0022 | 122.8395913 | 9.59E-30 | -0.00012 | 0.000214 | 0.66058 | 0.580135 |
| rs2642438 | G | A | 0.702657 | 0.025322 | 0.002273 | 118.0662873 | 8.02E-29 | 0.000141 | 0.000221 | 0.703454 | 0.523977 |
| rs2740488 | C | A | 0.265466 | -0.02525 | 0.002365 | 109.5230887 | 1.34E-26 | 0.000344 | 0.00023 | 0.264895 | 0.134506 |
| rs34042070 | G | C | 0.187927 | 0.048523 | 0.00268 | 316.8149481 | 2.92E-73 | -0.00013 | 0.000261 | 0.185684 | 0.627757 |
| rs35081008 | T | C | 0.14811 | -0.03191 | 0.002929 | 113.2290463 | 1.22E-27 | -0.00015 | 0.000288 | 0.146164 | 0.60712 |
| rs36043200 | A | G | 0.516694 | -0.02659 | 0.002092 | 155.6221772 | 5.06E-37 | -6.20E-05 | 0.000203 | 0.51601 | 0.760031 |
| rs430096 | G | A | 0.221046 | -0.05439 | 0.002514 | 449.2434412 | 8.63E-104 | 0.000147 | 0.000246 | 0.219168 | 0.549142 |
| rs4307732 | A | G | 0.105876 | 0.044832 | 0.003399 | 167.7051497 | 9.81E-40 | -0.00011 | 0.00033 | 0.105378 | 0.749048 |
| rs4452060 | A | C | 0.420697 | 0.083271 | 0.002104 | 1494.002462 | 0 | 0.000154 | 0.000205 | 0.419436 | 0.45493 |
| rs4665985 | C | A | 0.273129 | 0.024176 | 0.002359 | 102.2645043 | 1.18E-24 | -4.46E-05 | 0.00023 | 0.270158 | 0.846586 |
| rs472495 | T | G | 0.648959 | 0.042574 | 0.002181 | 364.1230994 | 7.26E-85 | 0.000233 | 0.000212 | 0.651167 | 0.272557 |
| rs4738684 | G | A | 0.664652 | -0.03154 | 0.002209 | 195.493704 | 3.03E-46 | 0.000167 | 0.000215 | 0.666079 | 0.436918 |
| rs4970834 | T | C | 0.186642 | -0.1053 | 0.002682 | 1488.18354 | 0 | -0.00043 | 0.000261 | 0.187629 | 0.100457 |
| rs556107 | T | C | 0.523338 | 0.035171 | 0.002088 | 272.0536591 | 1.23E-63 | -2.70E-05 | 0.000203 | 0.522698 | 0.894465 |
| rs56130071 | C | G | 0.216984 | 0.03318 | 0.002538 | 164.8646926 | 4.67E-39 | 0.000106 | 0.000245 | 0.220038 | 0.665539 |
| rs61775180 | T | C | 0.420137 | -0.02634 | 0.002111 | 148.9967057 | 9.65E-36 | -0.00029 | 0.000206 | 0.417758 | 0.161522 |
| rs62116889 | C | T | 0.068292 | -0.04958 | 0.004112 | 137.8418401 | 1.80E-33 | -0.00017 | 0.000404 | 0.067654 | 0.679551 |
| rs6544713 | C | T | 0.676864 | -0.05372 | 0.002223 | 556.7355228 | 5.04E-129 | 0.000143 | 0.000216 | 0.676138 | 0.508122 |
| rs6680227 | A | G | 0.035237 | -0.07466 | 0.005633 | 167.0157137 | 4.32E-40 | 0.00068 | 0.000552 | 0.034806 | 0.217742 |
| rs6709904 | G | A | 0.112585 | -0.04344 | 0.003296 | 166.184306 | 1.15E-39 | 0.000457 | 0.000323 | 0.111079 | 0.156495 |
| rs6874202 | C | T | 0.634269 | 0.032331 | 0.002163 | 213.748702 | 1.61E-50 | -0.00038 | 0.00021 | 0.635957 | 0.0730159 |
| rs71205205 | A | C | 0.11784 | 0.04546 | 0.003249 | 189.3655887 | 1.73E-44 | -0.00014 | 0.000314 | 0.118763 | 0.658524 |
| rs7202323 | G | T | 0.229741 | -0.02554 | 0.002477 | 101.6992069 | 6.48E-25 | 9.35E-05 | 0.00024 | 0.229879 | 0.697322 |
| rs7746081 | A | G | 0.304113 | -0.02347 | 0.002267 | 102.7573696 | 4.02E-25 | 0.000132 | 0.000221 | 0.301507 | 0.549258 |
| rs77542162 | G | A | 0.022495 | 0.128484 | 0.00704 | 320.0647002 | 2.06E-74 | 0.000143 | 0.000674 | 0.023077 | 0.831675 |
| rs79220007 | C | T | 0.076135 | -0.05734 | 0.003921 | 203.844097 | 2.04E-48 | -0.00073 | 0.000377 | 0.078096 | 0.0527084 |
| rs964184 | C | G | 0.866409 | -0.05758 | 0.003064 | 338.4004483 | 8.16E-79 | 7.38E-05 | 0.000298 | 0.867459 | 0.804348 |
| rs9987289 | G | A | 0.908808 | 0.045382 | 0.003622 | 150.4427871 | 5.07E-36 | -0.00014 | 0.000349 | 0.907683 | 0.696793 |

Abbreviations: SE, standard error; EAF, effect allele frequency; SNP, single nucleotide polymorphism; FS, frozen shoulder.

Supplement table 12. Summary data of SNPs used in MR analysis of IVs associated with LDL-frozen shoulder in males.

| **SNP** | **Effect Allele** | **Other Allele** | **EAF** | **Beta Estimate** | **SE** | **F-statistic** | **P.vale** | **beta.FS** | **se. FS** | **eaf. FS** | **pval. FS** |
| --- | --- | --- | --- | --- | --- | --- | --- | --- | --- | --- | --- |
| rs10423733 | C | T | 0.180142 | -0.10331 | 0.002708 | 1393.114377 | 0 | 0.000194 | 0.000227 | 0.180067 | 0.39409 |
| rs11206517 | G | T | 0.033149 | 0.068029 | 0.005806 | 130.7253793 | 1.05E-31 | 0.000655 | 0.000493 | 0.0322747 | 0.183938 |
| rs11591147 | T | G | 0.017468 | -0.34846 | 0.007931 | 1843.821775 | 0 | -0.00042 | 0.00066 | 0.0176805 | 0.520137 |
| rs1169294 | A | G | 0.310089 | 0.024504 | 0.002258 | 113.212638 | 1.99E-27 | 0.000208 | 0.000189 | 0.307131 | 0.272106 |
| rs117733303 | G | A | 0.018422 | 0.083818 | 0.007738 | 111.9602226 | 2.45E-27 | 0.000195 | 0.000646 | 0.0184619 | 0.762286 |
| rs118039278 | A | G | 0.078672 | 0.083506 | 0.003885 | 445.7837022 | 1.83E-102 | -5.50E-06 | 0.000322 | 0.0805994 | 0.986359 |
| rs12208357 | T | C | 0.070102 | 0.056984 | 0.004096 | 186.5861245 | 5.39E-44 | 0.000347 | 0.000344 | 0.0694905 | 0.313623 |
| rs12611067 | T | G | 0.351293 | -0.03174 | 0.002174 | 202.330015 | 2.97E-48 | -0.00011 | 0.000183 | 0.351727 | 0.555147 |
| rs12916 | C | T | 0.400537 | 0.062118 | 0.002127 | 817.8199832 | 1.75E-187 | 5.45E-05 | 0.000178 | 0.400096 | 0.759096 |
| rs1367117 | A | G | 0.335193 | 0.082872 | 0.0022 | 1352.560753 | 0 | 0.000124 | 0.000184 | 0.33831 | 0.500373 |
| rs1551891 | A | G | 0.088107 | -0.17363 | 0.00366 | 2144.480185 | 0 | -0.00013 | 0.000309 | 0.0873366 | 0.674285 |
| rs174564 | G | A | 0.348557 | -0.0319 | 0.00219 | 203.6521373 | 4.50E-48 | 1.98E-05 | 0.000183 | 0.350585 | 0.913404 |
| rs183130 | T | C | 0.323808 | -0.03295 | 0.00223 | 209.6053591 | 2.11E-49 | -0.00054 | 0.000186 | 0.325758 | 0.003647 |
| rs1883711 | C | G | 0.031412 | 0.10277 | 0.006092 | 283.3125912 | 7.49E-64 | 3.50E-05 | 0.000514 | 0.0308764 | 0.945717 |
| rs2618566 | T | G | 0.659861 | -0.02492 | 0.0022 | 122.8395913 | 9.59E-30 | 7.87E-05 | 0.000184 | 0.662822 | 0.668963 |
| rs2642438 | G | A | 0.702657 | 0.025322 | 0.002273 | 118.0662873 | 8.02E-29 | -0.00035 | 0.00019 | 0.701347 | 0.067647 |
| rs2740488 | C | A | 0.265466 | -0.02525 | 0.002365 | 109.5230887 | 1.34E-26 | 0.000229 | 0.000198 | 0.266174 | 0.24737 |
| rs28601761 | G | C | 0.418688 | -0.06201 | 0.002136 | 826.1582239 | 2.93E-185 | -6.04E-05 | 0.000179 | 0.417679 | 0.735509 |
| rs34042070 | G | C | 0.187927 | 0.048523 | 0.00268 | 316.8149481 | 2.92E-73 | -0.00013 | 0.000225 | 0.186631 | 0.569816 |
| rs35081008 | T | C | 0.14811 | -0.03191 | 0.002929 | 113.2290463 | 1.22E-27 | 3.35E-05 | 0.000247 | 0.147453 | 0.892289 |
| rs36043200 | A | G | 0.516694 | -0.02659 | 0.002092 | 155.6221772 | 5.06E-37 | 0.00016 | 0.000175 | 0.515805 | 0.361345 |
| rs430096 | G | A | 0.221046 | -0.05439 | 0.002514 | 449.2434412 | 8.63E-104 | -0.00028 | 0.000211 | 0.21884 | 0.179789 |
| rs4307732 | A | G | 0.105876 | 0.044832 | 0.003399 | 167.7051497 | 9.81E-40 | -0.0004 | 0.000285 | 0.105167 | 0.15732 |
| rs4452060 | A | C | 0.420697 | 0.083271 | 0.002104 | 1494.002462 | 0 | 0.000149 | 0.000177 | 0.42004 | 0.398943 |
| rs4665985 | C | A | 0.273129 | 0.024176 | 0.002359 | 102.2645043 | 1.18E-24 | 0.000128 | 0.000198 | 0.270655 | 0.516906 |
| rs472495 | T | G | 0.648959 | 0.042574 | 0.002181 | 364.1230994 | 7.26E-85 | -7.35E-05 | 0.000183 | 0.650839 | 0.688236 |
| rs4738684 | G | A | 0.664652 | -0.03154 | 0.002209 | 195.493704 | 3.03E-46 | -7.69E-06 | 0.000184 | 0.663337 | 0.966712 |
| rs4970834 | T | C | 0.186642 | -0.1053 | 0.002682 | 1488.18354 | 0 | -0.00022 | 0.000224 | 0.188024 | 0.322688 |
| rs556107 | T | C | 0.523338 | 0.035171 | 0.002088 | 272.0536591 | 1.23E-63 | -0.00013 | 0.000175 | 0.522533 | 0.462842 |
| rs56130071 | C | G | 0.216984 | 0.03318 | 0.002538 | 164.8646926 | 4.67E-39 | -9.57E-05 | 0.000212 | 0.217976 | 0.650936 |
| rs61775180 | T | C | 0.420137 | -0.02634 | 0.002111 | 148.9967057 | 9.65E-36 | 1.98E-05 | 0.000177 | 0.418883 | 0.910863 |
| rs62116889 | C | T | 0.068292 | -0.04958 | 0.004112 | 137.8418401 | 1.80E-33 | 0.000332 | 0.000349 | 0.0669599 | 0.340124 |
| rs6544713 | C | T | 0.676864 | -0.05372 | 0.002223 | 556.7355228 | 5.04E-129 | -3.32E-06 | 0.000186 | 0.676799 | 0.985756 |
| rs6680227 | A | G | 0.035237 | -0.07466 | 0.005633 | 167.0157137 | 4.32E-40 | -0.0001 | 0.000475 | 0.0346875 | 0.82564 |
| rs6709904 | G | A | 0.112585 | -0.04344 | 0.003296 | 166.184306 | 1.15E-39 | -0.0006 | 0.000278 | 0.110965 | 0.031217 |
| rs6874202 | C | T | 0.634269 | 0.032331 | 0.002163 | 213.748702 | 1.61E-50 | 3.44E-05 | 0.000181 | 0.635601 | 0.849261 |
| rs71205205 | A | C | 0.11784 | 0.04546 | 0.003249 | 189.3655887 | 1.73E-44 | -0.00039 | 0.000272 | 0.117316 | 0.155639 |
| rs7202323 | G | T | 0.229741 | -0.02554 | 0.002477 | 101.6992069 | 6.48E-25 | -0.00015 | 0.000208 | 0.227197 | 0.47117 |
| rs7746081 | A | G | 0.304113 | -0.02347 | 0.002267 | 102.7573696 | 4.02E-25 | -2.53E-05 | 0.00019 | 0.301411 | 0.893959 |
| rs77542162 | G | A | 0.022495 | 0.128484 | 0.00704 | 320.0647002 | 2.06E-74 | 0.000166 | 0.000583 | 0.0229284 | 0.776132 |
| rs79220007 | C | T | 0.076135 | -0.05734 | 0.003921 | 203.844097 | 2.04E-48 | -0.00053 | 0.000325 | 0.0780306 | 0.101996 |
| rs964184 | C | G | 0.866409 | -0.05758 | 0.003064 | 338.4004483 | 8.16E-79 | 0.00012 | 0.000257 | 0.868234 | 0.640018 |
| rs9987289 | G | A | 0.908808 | 0.045382 | 0.003622 | 150.4427871 | 5.07E-36 | -0.00046 | 0.000303 | 0.9088223 | 0.126261 |

Abbreviations: SE, standard error; EAF, effect allele frequency; SNP, single nucleotide polymorphism; FS, frozen shoulder.

Supplement table 13. Summary data of SNPs used in MR analysis of IVs associated with TG-frozen shoulder in females.

| **SNP** | **Effect Allele** | **Other Allele** | **EAF** | **Beta Estimate** | **SE** | **F-statistic** | **P.vale** | **beta.FS** | **se. FS** | **eaf. FS** | **pval. FS** |
| --- | --- | --- | --- | --- | --- | --- | --- | --- | --- | --- | --- |
| rs10773049 | C | T | 0.39535 | -0.02904 | 0.002037 | 177.8814046 | 4.07E-46 | 0.000188 | 0.000207 | 0.395126 | 0.363435 |
| rs1077835 | G | A | 0.220459 | 0.047367 | 0.002405 | 340.3551281 | 2.20E-86 | -0.0003 | 0.000245 | 0.219703 | 0.214262 |
| rs11122450 | G | T | 0.611733 | -0.04817 | 0.002037 | 486.730143 | 1.29E-123 | -2.93E-05 | 0.000208 | 0.613389 | 0.88816 |
| rs112259268 | A | C | 0.028291 | 0.139849 | 0.006014 | 474.7367786 | 1.26E-119 | -0.00021 | 0.000605 | 0.028951 | 0.722294 |
| rs114165349 | C | G | 0.023157 | 0.081813 | 0.006636 | 133.5861264 | 6.35E-35 | -3.95E-05 | 0.000671 | 0.023652 | 0.953042 |
| rs116843064 | A | G | 0.019327 | -0.22651 | 0.00721 | 859.3539166 | 1.26E-216 | -0.00032 | 0.000737 | 0.019161 | 0.662533 |
| rs12446515 | T | C | 0.322889 | -0.03342 | 0.002136 | 215.495763 | 3.47E-55 | 0.000178 | 0.000217 | 0.324087 | 0.411861 |
| rs12546096 | G | A | 0.252369 | 0.027897 | 0.00231 | 129.5550881 | 1.39E-33 | 0.000126 | 0.000237 | 0.250374 | 0.594214 |
| rs12928099 | A | C | 0.296442 | -0.02821 | 0.002181 | 146.4608846 | 2.92E-38 | -3.49E-05 | 0.000221 | 0.296782 | 0.874585 |
| rs13108218 | G | A | 0.614859 | -0.03052 | 0.002058 | 194.6032483 | 9.71E-50 | -0.00019 | 0.00021 | 0.618642 | 0.360624 |
| rs139974673 | C | T | 0.026131 | 0.143073 | 0.006239 | 459.9461767 | 2.17E-116 | 0.000214 | 0.000649 | 0.024996 | 0.741104 |
| rs150423652 | T | G | 0.006675 | 0.285824 | 0.012292 | 478.2920285 | 1.35E-119 | 0.001525 | 0.001258 | 0.00663 | 0.225406 |
| rs150564454 | A | G | 0.011556 | -0.10175 | 0.009632 | 104.333092 | 4.37E-26 | -0.00125 | 0.000993 | 0.011385 | 0.207832 |
| rs17184382 | C | A | 0.425367 | -0.02193 | 0.00201 | 103.6625687 | 1.08E-27 | -1.07E-05 | 0.000204 | 0.427127 | 0.958279 |
| rs174566 | G | A | 0.34974 | 0.048502 | 0.00208 | 472.3861553 | 2.76E-120 | 0.000341 | 0.000212 | 0.349943 | 0.10814 |
| rs17585887 | C | T | 0.591074 | -0.02858 | 0.002017 | 174.2664902 | 1.31E-45 | -8.12E-06 | 0.000206 | 0.590814 | 0.968511 |
| rs1801689 | C | A | 0.030543 | -0.06613 | 0.005777 | 114.2436707 | 2.44E-30 | -9.11E-05 | 0.000596 | 0.029726 | 0.878409 |
| rs186696265 | T | C | 0.014693 | -0.10432 | 0.008285 | 139.0015955 | 2.37E-36 | 0.000159 | 0.00084 | 0.014934 | 0.84947 |
| rs188247550 | T | C | 0.013117 | -0.13429 | 0.009131 | 205.9903323 | 5.89E-49 | -0.00095 | 0.000925 | 0.013291 | 0.304831 |
| rs2043085 | C | T | 0.612184 | -0.0308 | 0.002042 | 198.7770155 | 1.95E-51 | 0.000167 | 0.000208 | 0.613427 | 0.422025 |
| rs2068888 | A | G | 0.450732 | -0.03182 | 0.001996 | 221.2314638 | 3.16E-57 | 0.000219 | 0.000203 | 0.449512 | 0.282022 |
| rs2081687 | C | T | 0.663201 | -0.02614 | 0.002091 | 134.6394379 | 7.59E-36 | 0.000107 | 0.000214 | 0.664677 | 0.617178 |
| rs2130382 | G | C | 0.313212 | -0.02514 | 0.002145 | 119.9830712 | 9.65E-32 | 0.000423 | 0.000219 | 0.310378 | 0.053225 |
| rs2240466 | A | G | 0.122916 | -0.12277 | 0.003018 | 1437.881467 | 0 | 0.000294 | 0.000306 | 0.124131 | 0.337358 |
| rs2302263 | T | C | 0.088863 | 0.043611 | 0.003491 | 135.8679043 | 8.03E-36 | 0.000265 | 0.000354 | 0.089937 | 0.454468 |
| rs28383314 | C | T | 0.624109 | 0.037901 | 0.002047 | 297.4318175 | 1.48E-76 | 0.000162 | 0.000209 | 0.625107 | 0.438145 |
| rs28752924 | C | T | 0.445662 | 0.026556 | 0.002057 | 153.718527 | 3.96E-38 | 0.000127 | 0.000211 | 0.441032 | 0.547225 |
| rs308 | G | T | 0.020597 | -0.15941 | 0.006984 | 452.6272982 | 2.64E-115 | -0.00013 | 0.000706 | 0.021084 | 0.855294 |
| rs326222 | C | T | 0.697957 | 0.02522 | 0.002157 | 118.3032828 | 1.39E-31 | -0.00025 | 0.00022 | 0.696228 | 0.250282 |
| rs343 | A | C | 0.083129 | -0.14149 | 0.003611 | 1350.020909 | 0 | -0.0004 | 0.000371 | 0.082621 | 0.275752 |
| rs3775228 | T | C | 0.399717 | 0.03382 | 0.002036 | 242.1971223 | 5.83E-62 | -0.00022 | 0.000207 | 0.399616 | 0.295993 |
| rs4760254 | C | G | 0.23908 | -0.02814 | 0.002325 | 127.1104255 | 1.00E-33 | -0.00033 | 0.000236 | 0.242458 | 0.15933 |
| rs480823 | C | T | 0.078996 | 0.15572 | 0.00372 | 1561.611077 | 0 | -0.00041 | 0.000381 | 0.07821 | 0.283934 |
| rs4841580 | C | T | 0.434941 | -0.02454 | 0.002001 | 130.5641713 | 1.42E-34 | -8.15E-05 | 0.000205 | 0.43535 | 0.690831 |
| rs6073958 | C | T | 0.198674 | 0.055695 | 0.002493 | 436.0108865 | 1.63E-110 | 7.77E-08 | 0.000254 | 0.198407 | 0.999756 |
| rs61905078 | C | A | 0.073841 | 0.199898 | 0.003795 | 2423.608898 | 0 | -8.32E-06 | 0.000388 | 0.073542 | 0.982898 |
| rs676210 | A | G | 0.205293 | -0.07352 | 0.00245 | 779.120256 | 7.09E-198 | 0.000111 | 0.000251 | 0.203985 | 0.659231 |
| rs6800707 | G | C | 0.810543 | 0.029885 | 0.002538 | 121.0009935 | 5.35E-32 | 0.00053 | 0.000258 | 0.809748 | 0.039694 |
| rs684773 | C | A | 0.766797 | 0.029113 | 0.002347 | 133.7246434 | 2.50E-35 | 0.000439 | 0.000239 | 0.767214 | 0.066483 |
| rs6882076 | C | T | 0.634285 | 0.033101 | 0.002061 | 224.2927617 | 4.95E-58 | -0.00038 | 0.00021 | 0.635953 | 0.073147 |
| rs6999569 | G | A | 0.470737 | -0.08609 | 0.001981 | 1634.667045 | 0 | 8.74E-05 | 0.000203 | 0.474515 | 0.666673 |
| rs7000494 | C | G | 0.030042 | 0.136538 | 0.005842 | 479.6708457 | 7.96E-121 | -0.00045 | 0.000599 | 0.029865 | 0.45304 |
| rs7140110 | C | T | 0.298338 | 0.028317 | 0.002178 | 148.1035068 | 1.21E-38 | -1.04E-05 | 0.000221 | 0.296938 | 0.962446 |
| rs72555385 | G | A | 0.048975 | 0.065378 | 0.004618 | 175.6627387 | 1.67E-45 | -4.25E-06 | 0.000472 | 0.048612 | 0.992824 |
| rs73243877 | G | A | 0.168135 | 0.028812 | 0.002656 | 102.4311336 | 2.00E-27 | -0.00059 | 0.000271 | 0.167824 | 0.028948 |
| rs75609851 | A | G | 0.01048 | -0.19912 | 0.01004 | 362.9424906 | 1.57E-87 | 0.000715 | 0.001032 | 0.010394 | 0.48826 |
| rs77009508 | G | A | 0.073734 | 0.045091 | 0.0038 | 122.5152379 | 1.77E-32 | 0.000141 | 0.000386 | 0.074449 | 0.715113 |
| rs7786102 | A | G | 0.715703 | -0.02837 | 0.002201 | 144.4884438 | 5.12E-38 | 0.000378 | 0.000233 | 0.718258 | 0.105193 |
| rs78484485 | A | G | 0.054016 | -0.07585 | 0.004399 | 259.4707484 | 1.23E-66 | 0.000291 | 0.000452 | 0.052986 | 0.519457 |
| rs9436661 | G | T | 0.352919 | -0.07773 | 0.002078 | 1220.444876 | 2.84E-306 | -0.00013 | 0.000212 | 0.354822 | 0.533981 |
| rs998584 | A | C | 0.482727 | 0.040118 | 0.00199 | 354.7609966 | 2.20E-90 | -0.00016 | 0.000203 | 0.482308 | 0.424391 |

Abbreviations: SE, standard error; EAF, effect allele frequency; SNP, single nucleotide polymorphism; FS, frozen shoulder.

Supplement table 14. Summary data of SNPs used in MR analysis of IVs associated with TG-frozen shoulder in males.

| **SNP** | **Effect Allele** | **Other Allele** | **EAF** | **Beta Estimate** | **SE** | **F-statistic** | **P.vale** | **beta.FS** | **se. FS** | **eaf. FS** | **pval. FS** |
| --- | --- | --- | --- | --- | --- | --- | --- | --- | --- | --- | --- |
| rs10773049 | C | T | 0.39535 | -0.02904 | 0.002037 | 177.8814046 | 4.07E-46 | -0.000359936 | 0.000178616 | 0.395574 | 0.0438919 |
| rs1077835 | G | A | 0.220459 | 0.047367 | 0.002405 | 340.3551281 | 2.20E-86 | 0.000571935 | 0.000210541 | 0.219653 | 0.00659833 |
| rs11122450 | G | T | 0.611733 | -0.04817 | 0.002037 | 486.730143 | 1.29E-123 | 0.000111204 | 0.000178823 | 0.612748 | 0.53403 |
| rs112259268 | A | C | 0.028291 | 0.139849 | 0.006014 | 474.7367786 | 1.26E-119 | 0.000408259 | 0.00052315 | 0.0286919 | 0.435165 |
| rs114165349 | C | G | 0.023157 | 0.081813 | 0.006636 | 133.5861264 | 6.35E-35 | -0.000895017 | 0.000581497 | 0.0232534 | 0.123767 |
| rs116843064 | A | G | 0.019327 | -0.22651 | 0.00721 | 859.3539166 | 1.26E-216 | 0.000487045 | 0.00061996 | 0.0200665 | 0.432099 |
| rs12446515 | T | C | 0.322889 | -0.03342 | 0.002136 | 215.495763 | 3.47E-55 | -0.000530392 | 0.000186806 | 0.324841 | 0.00452229 |
| rs12546096 | G | A | 0.252369 | 0.027897 | 0.00231 | 129.5550881 | 1.39E-33 | 0.000136862 | 0.000203825 | 0.250537 | 0.501923 |
| rs12928099 | A | C | 0.296442 | -0.02821 | 0.002181 | 146.4608846 | 2.92E-38 | -0.000186221 | 0.000191705 | 0.295365 | 0.331353 |
| rs13108218 | G | A | 0.614859 | -0.03052 | 0.002058 | 194.6032483 | 9.71E-50 | 1.01E-05 | 0.000180938 | 0.617771 | 0.955584 |
| rs139974673 | C | T | 0.026131 | 0.143073 | 0.006239 | 459.9461767 | 2.17E-116 | 0.000465374 | 0.000560223 | 0.0249132 | 0.406148 |
| rs150423652 | T | G | 0.006675 | 0.285824 | 0.012292 | 478.2920285 | 1.35E-119 | -0.000699488 | 0.00107832 | 0.00667519 | 0.516543 |
| rs150564454 | A | G | 0.011556 | -0.10175 | 0.009632 | 104.333092 | 4.37E-26 | -0.000464565 | 0.000854121 | 0.0114244 | 0.586505 |
| rs17184382 | C | A | 0.425367 | -0.02193 | 0.00201 | 103.6625687 | 1.08E-27 | -0.000212215 | 0.000175995 | 0.426335 | 0.227897 |
| rs174566 | G | A | 0.34974 | 0.048502 | 0.00208 | 472.3861553 | 2.76E-120 | -1.14E-05 | 0.000182401 | 0.351636 | 0.950079 |
| rs17585887 | C | T | 0.591074 | -0.02858 | 0.002017 | 174.2664902 | 1.31E-45 | 0.000130794 | 0.000176803 | 0.589993 | 0.459437 |
| rs1801689 | C | A | 0.030543 | -0.06613 | 0.005777 | 114.2436707 | 2.44E-30 | -0.000895911 | 0.000509871 | 0.0300802 | 0.0788967 |
| rs186696265 | T | C | 0.014693 | -0.10432 | 0.008285 | 139.0015955 | 2.37E-36 | -0.00024156 | 0.000721798 | 0.0148814 | 0.737879 |
| rs188247550 | T | C | 0.013117 | -0.13429 | 0.009131 | 205.9903323 | 5.89E-49 | -0.000266446 | 0.000792166 | 0.0133316 | 0.736606 |
| rs2043085 | C | T | 0.612184 | -0.0308 | 0.002042 | 198.7770155 | 1.95E-51 | -3.13E-05 | 0.000178551 | 0.610738 | 0.860944 |
| rs2068888 | A | G | 0.450732 | -0.03182 | 0.001996 | 221.2314638 | 3.16E-57 | 0.00043945 | 0.000175041 | 0.447734 | 0.0120552 |
| rs2081687 | C | T | 0.663201 | -0.02614 | 0.002091 | 134.6394379 | 7.59E-36 | 2.38E-05 | 0.000183895 | 0.661975 | 0.897101 |
| rs2130382 | G | C | 0.313212 | -0.02514 | 0.002145 | 119.9830712 | 9.65E-32 | -7.37E-06 | 0.000187873 | 0.313064 | 0.968689 |
| rs2240466 | A | G | 0.122916 | -0.12277 | 0.003018 | 1437.881467 | 0 | -0.000115209 | 0.000264502 | 0.123392 | 0.663149 |
| rs2302263 | T | C | 0.088863 | 0.043611 | 0.003491 | 135.8679043 | 8.03E-36 | -9.22E-06 | 0.000309361 | 0.0873148 | 0.976212 |
| rs28383314 | C | T | 0.624109 | 0.037901 | 0.002047 | 297.4318175 | 1.48E-76 | -1.14E-05 | 0.000179494 | 0.6233 | 0.949309 |
| rs28752924 | C | T | 0.445662 | 0.026556 | 0.002057 | 153.718527 | 3.96E-38 | 2.52E-05 | 0.00018142 | 0.440007 | 0.889666 |
| rs308 | G | T | 0.020597 | -0.15941 | 0.006984 | 452.6272982 | 2.64E-115 | -0.000168311 | 0.000618525 | 0.0203627 | 0.785533 |
| rs326222 | C | T | 0.697957 | 0.02522 | 0.002157 | 118.3032828 | 1.39E-31 | 9.16E-05 | 0.000189431 | 0.696976 | 0.628821 |
| rs343 | A | C | 0.083129 | -0.14149 | 0.003611 | 1350.020909 | 0 | -0.000150482 | 0.000318901 | 0.0824421 | 0.637014 |
| rs3775228 | T | C | 0.399717 | 0.03382 | 0.002036 | 242.1971223 | 5.83E-62 | 0.000163149 | 0.000178427 | 0.400048 | 0.360524 |
| rs4760254 | C | G | 0.23908 | -0.02814 | 0.002325 | 127.1104255 | 1.00E-33 | -1.30E-05 | 0.000203617 | 0.240849 | 0.949201 |
| rs480823 | C | T | 0.078996 | 0.15572 | 0.00372 | 1561.611077 | 0 | -0.000118938 | 0.000327226 | 0.0789161 | 0.716251 |
| rs4841580 | C | T | 0.434941 | -0.02454 | 0.002001 | 130.5641713 | 1.42E-34 | -0.000126696 | 0.000176111 | 0.43298 | 0.471889 |
| rs6073958 | C | T | 0.198674 | 0.055695 | 0.002493 | 436.0108865 | 1.63E-110 | -0.000170403 | 0.000218382 | 0.198788 | 0.435217 |
| rs61905078 | C | A | 0.073841 | 0.199898 | 0.003795 | 2423.608898 | 0 | 0.00016405 | 0.000333716 | 0.0734691 | 0.623012 |
| rs676210 | A | G | 0.205293 | -0.07352 | 0.00245 | 779.120256 | 7.09E-198 | -0.000209221 | 0.000215957 | 0.204431 | 0.332643 |
| rs6800707 | G | C | 0.810543 | 0.029885 | 0.002538 | 121.0009935 | 5.35E-32 | -1.90E-05 | 0.000222206 | 0.809305 | 0.932036 |
| rs684773 | C | A | 0.766797 | 0.029113 | 0.002347 | 133.7246434 | 2.50E-35 | -6.29E-05 | 0.000205752 | 0.766675 | 0.759925 |
| rs6882076 | C | T | 0.634285 | 0.033101 | 0.002061 | 224.2927617 | 4.95E-58 | 3.45E-05 | 0.000180974 | 0.635595 | 0.848697 |
| rs6999569 | G | A | 0.470737 | -0.08609 | 0.001981 | 1634.667045 | 0 | -9.53E-05 | 0.000174613 | 0.471746 | 0.585058 |
| rs7000494 | C | G | 0.030042 | 0.136538 | 0.005842 | 479.6708457 | 7.96E-121 | -0.000335501 | 0.000515265 | 0.0299961 | 0.514968 |
| rs7140110 | C | T | 0.298338 | 0.028317 | 0.002178 | 148.1035068 | 1.21E-38 | 0.000331809 | 0.000190733 | 0.296982 | 0.0819228 |
| rs72555385 | G | A | 0.048975 | 0.065378 | 0.004618 | 175.6627387 | 1.67E-45 | -0.000386464 | 0.00040453 | 0.0489619 | 0.339407 |
| rs73243877 | G | A | 0.168135 | 0.028812 | 0.002656 | 102.4311336 | 2.00E-27 | 0.000621877 | 0.000232536 | 0.167719 | 0.00748875 |
| rs75609851 | A | G | 0.01048 | -0.19912 | 0.01004 | 362.9424906 | 1.57E-87 | -0.000760623 | 0.000882043 | 0.0105077 | 0.3885 |
| rs77009508 | G | A | 0.073734 | 0.045091 | 0.0038 | 122.5152379 | 1.77E-32 | 7.79E-06 | 0.000329013 | 0.0758891 | 0.98112 |
| rs7786102 | A | G | 0.715703 | -0.02837 | 0.002201 | 144.4884438 | 5.12E-38 | 0.000342552 | 0.00020021 | 0.717869 | 0.0870893 |
| rs78484485 | A | G | 0.054016 | -0.07585 | 0.004399 | 259.4707484 | 1.23E-66 | 0.00034844 | 0.000386917 | 0.0539146 | 0.367825 |
| rs9436661 | G | T | 0.352919 | -0.07773 | 0.002078 | 1220.444876 | 2.84E-306 | -0.000138509 | 0.000182146 | 0.355007 | 0.447 |
| rs998584 | A | C | 0.482727 | 0.040118 | 0.00199 | 354.7609966 | 2.20E-90 | -0.000276028 | 0.000174604 | 0.481018 | 0.113907 |

Abbreviations: SE, standard error; EAF, effect allele frequency; SNP, single nucleotide polymorphism; FS, frozen shoulder.

Supplement table 15. Summary data of SNPs used in MR analysis of IVs associated with frozen shoulder-HDL.

| **SNP** | **Effect Allele** | **Other Allele** | **EAF** | **Beta Estimate** | **SE** | **F-statistic** | **P.vale** | **beta. HDL** | **se. HDL** | **eaf. HDL** | **pval. HDL** |
| --- | --- | --- | --- | --- | --- | --- | --- | --- | --- | --- | --- |
| rs1042704 | A | G | 0.784999 | 0.103074 | 0.016909 | 1623.559 | 1.09E-09 | 0.001945 | 0.002332 | 0.213953 | 0.4 |
| rs111481541 | C | T | 0.971361 | -0.19751 | 0.042067 | 981.2261 | 2.66E-06 | 0.002835 | 0.005771 | 0.028556 | 0.62 |
| rs117557731 | G | A | 0.963514 | 0.170286 | 0.037039 | 921.5702 | 4.28E-06 | -0.00717 | 0.005107 | 0.036072 | 0.16 |
| rs117999064 | G | A | 0.983692 | 0.304439 | 0.05549 | 1345.407 | 4.10E-08 | 0.013766 | 0.00768 | 0.016167 | 0.073 |
| rs12915503 | C | T | 0.745019 | 0.086278 | 0.0161 | 1279.409 | 8.38E-08 | 0.001184 | 0.002204 | 0.25633 | 0.59 |
| rs1929880 | A | C | 0.241854 | -0.07525 | 0.016242 | 938.7018 | 3.60E-06 | 0.003022 | 0.002226 | 0.757235 | 0.17 |
| rs2598109 | T | C | 0.161047 | 0.096864 | 0.018882 | 1146.617 | 2.90E-07 | 0.001447 | 0.002595 | 0.839269 | 0.58 |
| rs2746117 | A | G | 0.589134 | 0.064676 | 0.014127 | 915.3481 | 4.69E-06 | 0.001368 | 0.001944 | 0.411043 | 0.48 |
| rs296724 | T | C | 0.227306 | -0.07932 | 0.016577 | 999.0837 | 1.71E-06 | -0.00401 | 0.002272 | 0.77134 | 0.077999 |
| rs41271365 | C | T | 0.78792 | 0.078834 | 0.016961 | 938.8886 | 3.35E-06 | -0.00377 | 0.002328 | 0.211867 | 0.11 |
| rs4959559 | G | A | 0.787804 | 0.079786 | 0.017149 | 962.1378 | 3.28E-06 | -0.00293 | 0.00235 | 0.213195 | 0.21 |
| rs530125017 | C | T | 0.988891 | 0.356925 | 0.067372 | 1266.177 | 1.17E-07 | -0.00957 | 0.009264 | 0.011052 | 0.3 |
| rs592294 | G | A | 0.446147 | -0.06765 | 0.014273 | 1022.715 | 2.14E-06 | 0.003745 | 0.001963 | 0.553754 | 0.056 |
| rs61306957 | C | G | 0.987402 | 0.314039 | 0.062276 | 1109.506 | 4.59E-07 | -0.00261 | 0.008337 | 0.986826 | 0.75 |
| rs61811976 | C | A | 0.914113 | -0.12057 | 0.024801 | 1032.01 | 1.17E-06 | -0.00268 | 0.003405 | 0.085442 | 0.43 |
| rs62290785 | T | G | 0.975752 | 0.21287 | 0.045317 | 969.3407 | 2.64E-06 | 0.001026 | 0.006262 | 0.023971 | 0.87 |
| rs667828 | A | G | 0.609276 | 0.064852 | 0.014223 | 905.096 | 5.13E-06 | 0.003033 | 0.001951 | 0.390236 | 0.12 |
| rs6731576 | C | T | 0.204159 | 0.080267 | 0.0173 | 946.3976 | 3.49E-06 | -0.00295 | 0.002364 | 0.794688 | 0.21 |
| rs73346492 | C | T | 0.984211 | -0.25407 | 0.055643 | 906.8213 | 4.97E-06 | -0.01089 | 0.007585 | 0.016045 | 0.15 |
| rs79292884 | T | G | 0.986134 | 0.286788 | 0.059309 | 1016.92 | 1.33E-06 | -0.00639 | 0.008126 | 0.013944 | 0.43 |
| rs9929820 | T | C | 0.735945 | 0.07846 | 0.015813 | 1081.882148 | 6.98E-07 | -0.00283 | 0.002142 | 0.265484 | 0.19 |

Abbreviations: SE, standard error; EAF, effect allele frequency; SNP, single nucleotide polymorphism.

Supplement table 16. Summary data of SNPs used in MR analysis of IVs associated with frozen shoulder-LDL.

| **SNP** | **Effect Allele** | **Other Allele** | **EAF** | **Beta Estimate** | **SE** | **F-statistic** | **P.vale** | **beta. LDL** | **se. LDL** | **eaf. LDL** | **pval. LDL** |
| --- | --- | --- | --- | --- | --- | --- | --- | --- | --- | --- | --- |
| rs1042704 | A | G | 0.784999 | 0.103074 | 0.016909 | 1623.559 | 1.09E-09 | -0.00044 | 0.002546 | 0.213771 | 0.86 |
| rs111481541 | C | T | 0.971361 | -0.19751 | 0.042067 | 981.2261 | 2.66E-06 | -0.00118 | 0.006305 | 0.028625 | 0.85 |
| rs117557731 | G | A | 0.963514 | 0.170286 | 0.037039 | 921.5702 | 4.28E-06 | 0.000576 | 0.005603 | 0.035986 | 0.92 |
| rs117999064 | G | A | 0.983692 | 0.304439 | 0.05549 | 1345.407 | 4.10E-08 | -0.01449 | 0.008389 | 0.016123 | 0.084 |
| rs12915503 | C | T | 0.745019 | 0.086278 | 0.0161 | 1279.409 | 8.38E-08 | -0.00594 | 0.002415 | 0.256374 | 0.014 |
| rs1929880 | A | C | 0.241854 | -0.07525 | 0.016242 | 938.7018 | 3.60E-06 | 0.003467 | 0.002436 | 0.75715 | 0.15 |
| rs2598109 | T | C | 0.161047 | 0.096864 | 0.018882 | 1146.617 | 2.90E-07 | -0.00095 | 0.002839 | 0.839285 | 0.74 |
| rs2746117 | A | G | 0.589134 | 0.064676 | 0.014127 | 915.3481 | 4.69E-06 | 0.003112 | 0.002123 | 0.410888 | 0.14 |
| rs296724 | T | C | 0.227306 | -0.07932 | 0.016577 | 999.0837 | 1.71E-06 | -0.00156 | 0.002486 | 0.771394 | 0.53 |
| rs41271365 | C | T | 0.78792 | 0.078834 | 0.016961 | 938.8886 | 3.35E-06 | -0.00112 | 0.002549 | 0.211738 | 0.66 |
| rs4959559 | G | A | 0.787804 | 0.079786 | 0.017149 | 962.1378 | 3.28E-06 | -0.0007 | 0.00257 | 0.213297 | 0.780001 |
| rs530125017 | C | T | 0.988891 | 0.356925 | 0.067372 | 1266.177 | 1.17E-07 | -0.00998 | 0.010129 | 0.011091 | 0.32 |
| rs592294 | G | A | 0.446147 | -0.06765 | 0.014273 | 1022.715 | 2.14E-06 | -0.00063 | 0.002146 | 0.553767 | 0.77 |
| rs61306957 | C | G | 0.987402 | 0.314039 | 0.062276 | 1109.506 | 4.59E-07 | -0.00756 | 0.009105 | 0.986776 | 0.41 |
| rs61811976 | C | A | 0.914113 | -0.12057 | 0.024801 | 1032.01 | 1.17E-06 | -0.00324 | 0.003726 | 0.085321 | 0.39 |
| rs62290785 | T | G | 0.975752 | 0.21287 | 0.045317 | 969.3407 | 2.64E-06 | -0.00097 | 0.006848 | 0.023979 | 0.89 |
| rs6330 | A | G | 0.538728 | 0.076142 | 0.013916 | 1303.548 | 4.46E-08 | 0.000283 | 0.002084 | 0.459762 | 0.89 |
| rs667828 | A | G | 0.609276 | 0.064852 | 0.014223 | 905.096 | 5.13E-06 | 0.001115 | 0.002135 | 0.39043 | 0.6 |
| rs6731576 | C | T | 0.204159 | 0.080267 | 0.0173 | 946.3976 | 3.49E-06 | -0.00238 | 0.002587 | 0.794745 | 0.36 |
| rs73346492 | C | T | 0.984211 | -0.25407 | 0.055643 | 906.8213 | 4.97E-06 | -0.00743 | 0.008297 | 0.016034 | 0.37 |
| rs79292884 | T | G | 0.986134 | 0.286788 | 0.059309 | 1016.92 | 1.33E-06 | -0.01168 | 0.008902 | 0.0139 | 0.19 |
| rs9264971 | T | C | 0.308958 | 0.070224 | 0.015308 | 951.8926 | 4.48E-06 | 0.001753 | 0.002297 | 0.691114 | 0.450001 |
| rs9929820 | T | C | 0.735945 | 0.07846 | 0.015813 | 1081.882148 | 6.98E-07 | -0.00322 | 0.002371 | 0.265572 | 0.17 |

Abbreviations: SE, standard error; EAF, effect allele frequency; SNP, single nucleotide polymorphism.

Supplement table 17. Summary data of SNPs used in MR analysis of IVs associated with frozen shoulder-TG.

| **SNP** | **Effect Allele** | **Other Allele** | **EAF** | **Beta Estimate** | **SE** | **F-statistic** | **P.vale** | **beta. TG** | **se. TG** | **eaf. TG** | **pval. TG** |
| --- | --- | --- | --- | --- | --- | --- | --- | --- | --- | --- | --- |
| rs1042704 | A | G | 0.784999 | 0.103074 | 0.016909 | 1623.559 | 1.09E-09 | -0.0044 | 0.002429 | 0.213767 | 0.07 |
| rs111481541 | C | T | 0.971361 | -0.19751 | 0.042067 | 981.2261 | 2.66E-06 | -0.00836 | 0.006005 | 0.028619 | 0.16 |
| rs117557731 | G | A | 0.963514 | 0.170286 | 0.037039 | 921.5702 | 4.28E-06 | 0.002713 | 0.005325 | 0.036001 | 0.61 |
| rs12915503 | C | T | 0.745019 | 0.086278 | 0.0161 | 1279.409 | 8.38E-08 | 0.005174 | 0.002302 | 0.256362 | 0.025 |
| rs1929880 | A | C | 0.241854 | -0.07525 | 0.016242 | 938.7018 | 3.60E-06 | -0.00044 | 0.002323 | 0.757141 | 0.85 |
| rs2598109 | T | C | 0.161047 | 0.096864 | 0.018882 | 1146.617 | 2.90E-07 | -0.00178 | 0.002703 | 0.839253 | 0.51 |
| rs2746117 | A | G | 0.589134 | 0.064676 | 0.014127 | 915.3481 | 4.69E-06 | -0.00344 | 0.002026 | 0.410922 | 0.089 |
| rs296724 | T | C | 0.227306 | -0.07932 | 0.016577 | 999.0837 | 1.71E-06 | -0.00045 | 0.002371 | 0.771413 | 0.85 |
| rs41271365 | C | T | 0.78792 | 0.078834 | 0.016961 | 938.8886 | 3.35E-06 | -0.00069 | 0.00243 | 0.211708 | 0.780001 |
| rs4959559 | G | A | 0.787804 | 0.079786 | 0.017149 | 962.1378 | 3.28E-06 | 0.003046 | 0.002448 | 0.21328 | 0.21 |
| rs530125017 | C | T | 0.988891 | 0.356925 | 0.067372 | 1266.177 | 1.17E-07 | -0.00967 | 0.009651 | 0.011099 | 0.32 |
| rs592294 | G | A | 0.446147 | -0.06765 | 0.014273 | 1022.715 | 2.14E-06 | -0.00226 | 0.002046 | 0.553741 | 0.27 |
| rs61306957 | C | G | 0.987402 | 0.314039 | 0.062276 | 1109.506 | 4.59E-07 | -0.00553 | 0.008668 | 0.986771 | 0.52 |
| rs61811976 | C | A | 0.914113 | -0.12057 | 0.024801 | 1032.01 | 1.17E-06 | 0.000216 | 0.003554 | 0.085308 | 0.95 |
| rs62290785 | T | G | 0.975752 | 0.21287 | 0.045317 | 969.3407 | 2.64E-06 | 0.004901 | 0.006526 | 0.023973 | 0.450001 |
| rs6330 | A | G | 0.538728 | 0.076142 | 0.013916 | 1303.548 | 4.46E-08 | -0.00032 | 0.001988 | 0.459735 | 0.87 |
| rs667828 | A | G | 0.609276 | 0.064852 | 0.014223 | 905.096 | 5.13E-06 | 0.000705 | 0.002034 | 0.390478 | 0.73 |
| rs6731576 | C | T | 0.204159 | 0.080267 | 0.0173 | 946.3976 | 3.49E-06 | -0.00341 | 0.002464 | 0.794743 | 0.17 |
| rs73346492 | C | T | 0.984211 | -0.25407 | 0.055643 | 906.8213 | 4.97E-06 | 0.001712 | 0.007908 | 0.016029 | 0.83 |
| rs79292884 | T | G | 0.986134 | 0.286788 | 0.059309 | 1016.92 | 1.33E-06 | -0.0122 | 0.008482 | 0.013901 | 0.15 |
| rs9929820 | T | C | 0.735945 | 0.07846 | 0.015813 | 1081.882148 | 6.98E-07 | 0.00319 | 0.002261 | 0.265591 | 0.16 |

Abbreviations: SE, standard error; EAF, effect allele frequency; SNP, single nucleotide polymorphism.

Supplement table 18. Summary data of SNPs used in MR analysis of IVs associated with frozen shoulder-HDL in females.

| **SNP** | **Effect Allele** | **Other Allele** | **EAF** | **Beta Estimate** | **SE** | **F-statistic** | **P.vale** | **beta. HDL** | **se. HDL** | **eaf. HDL** | **pval. HDL** |
| --- | --- | --- | --- | --- | --- | --- | --- | --- | --- | --- | --- |
| rs1042704 | A | G | 0.784999 | 0.103074 | 0.016909 | 1623.558928 | 1.09E-09 | 0.002932 | 0.004186 | 0.21553 | 0.4837 |
| rs111481541 | C | T | 0.971361 | -0.197512 | 0.042067 | 981.2260539 | 2.66E-06 | 0.015918 | 0.010445 | 0.028289 | 0.12754 |
| rs117557731 | G | A | 0.963514 | 0.170286 | 0.037039 | 921.5702453 | 4.28E-06 | -0.0094509 | 0.009138 | 0.036583 | 0.30103 |
| rs12915503 | C | T | 0.745019 | 0.0862784 | 0.0161 | 1279.408637 | 8.38E-08 | 0.0039109 | 0.004096 | 0.25389 | 0.33962 |
| rs13151266 | A | C | 0.755331 | -0.0773496 | 0.016169 | 999.7554641 | 1.72E-06 | -0.00064868 | 0.003999 | 0.2454 | 0.87113 |
| rs1929880 | A | C | 0.241854 | -0.0752505 | 0.016242 | 938.7017657 | 3.60E-06 | 0.0026972 | 0.004034 | 0.75992 | 0.50369 |
| rs2598109 | T | C | 0.161047 | 0.0968641 | 0.018882 | 1146.617082 | 2.90E-07 | 0.0063728 | 0.004667 | 0.83898 | 0.17205 |
| rs2746117 | A | G | 0.589134 | 0.0646764 | 0.014127 | 915.3480543 | 4.69E-06 | -0.00057059 | 0.003498 | 0.41132 | 0.87041 |
| rs28971325 | A | G | 0.766755 | 0.184128 | 0.016444 | 5537.4175 | 4.20E-29 | 0.0014002 | 0.004068 | 0.23363 | 0.7307 |
| rs296724 | T | C | 0.227306 | -0.0793161 | 0.016577 | 999.0837228 | 1.71E-06 | 0.00010082 | 0.004107 | 0.77317 | 0.98042 |
| rs41271365 | C | T | 0.78792 | 0.0788342 | 0.016961 | 938.8886321 | 3.35E-06 | -0.00052232 | 0.004198 | 0.21224 | 0.90097 |
| rs4959559 | G | A | 0.787804 | 0.0797863 | 0.017149 | 962.1377922 | 3.28E-06 | 0.0017858 | 0.004257 | 0.21069 | 0.67488 |
| rs530125017 | C | T | 0.988891 | 0.356925 | 0.067372 | 1266.177434 | 1.17E-07 | -0.016175 | 0.016551 | 0.011279 | 0.32842 |
| rs56248155 | G | C | 0.598251 | -0.0673744 | 0.014506 | 986.4536102 | 3.41E-06 | 0.00077936 | 0.003591 | 0.599 | 0.82817 |
| rs592294 | G | A | 0.446147 | -0.0676549 | 0.014273 | 1022.714569 | 2.14E-06 | 0.0064116 | 0.003535 | 0.55325 | 0.069723 |
| rs61306957 | C | G | 0.987402 | 0.314039 | 0.062276 | 1109.505808 | 4.59E-07 | 0.012216 | 0.015625 | 0.987745 | 0.43434 |
| rs61811976 | C | A | 0.914113 | -0.120568 | 0.024801 | 1032.009752 | 1.17E-06 | -0.003507 | 0.006109 | 0.086732 | 0.56591 |
| rs62290785 | T | G | 0.975752 | 0.21287 | 0.045317 | 969.3407306 | 2.64E-06 | 0.003401 | 0.01127 | 0.024017 | 0.76282 |
| rs6330 | A | G | 0.538728 | 0.0761419 | 0.013916 | 1303.548455 | 4.46E-08 | 0.0055357 | 0.003449 | 0.46152 | 0.10851 |
| rs667828 | A | G | 0.609276 | 0.0648515 | 0.014223 | 905.0960201 | 5.13E-06 | -0.0042006 | 0.003525 | 0.39033 | 0.23338 |
| rs6731576 | C | T | 0.204159 | 0.0802665 | 0.0173 | 946.3975719 | 3.49E-06 | -0.00048782 | 0.004292 | 0.79614 | 0.90951 |
| rs73346492 | C | T | 0.984211 | -0.25407 | 0.055643 | 906.8213084 | 4.97E-06 | -0.0196 | 0.013872 | 0.015569 | 0.15767 |
| rs79292884 | T | G | 0.986134 | 0.286788 | 0.059309 | 1016.920386 | 1.33E-06 | -0.004391 | 0.014568 | 0.014064 | 0.7631 |
| rs9264971 | T | C | 0.308958 | 0.0702238 | 0.015308 | 951.8925927 | 4.48E-06 | -0.013528 | 0.003785 | 0.69013 | 0.00035099 |
| rs9929820 | T | C | 0.735945 | 0.0784603 | 0.015813 | 1081.882148 | 6.98E-07 | -0.003384 | 0.003905 | 0.26405 | 0.38613 |

Abbreviations: SE, standard error; EAF, effect allele frequency; SNP, single nucleotide polymorphism.

Supplement table 19. Summary data of SNPs used in MR analysis of IVs associated with frozen shoulder-HDL in males.

| **SNP** | **Effect Allele** | **Other Allele** | **EAF** | **Beta Estimate** | **SE** | **F-statistic** | **P.vale** | **beta. HDL** | **se. HDL** | **eaf. HDL** | **pval. HDL** |
| --- | --- | --- | --- | --- | --- | --- | --- | --- | --- | --- | --- |
| rs1042704 | A | G | 0.784999 | 0.103074 | 0.016909 | 1623.558928 | 1.09E-09 | -4.62E-05 | 0.0044756 | 0.21612 | 0.99177 |
| rs111481541 | C | T | 0.971361 | -0.197512 | 0.042067 | 981.2260539 | 2.66E-06 | -0.0096948 | 0.011159 | 0.028817 | 0.38498 |
| rs117557731 | G | A | 0.963514 | 0.170286 | 0.037039 | 921.5702453 | 4.28E-06 | 0.004102 | 0.0098286 | 0.036632 | 0.67642 |
| rs12915503 | C | T | 0.745019 | 0.0862784 | 0.0161 | 1279.408637 | 8.38E-08 | 0.0026159 | 0.0043923 | 0.25261 | 0.55147 |
| rs13151266 | A | C | 0.755331 | -0.0773496 | 0.016169 | 999.7554641 | 1.72E-06 | 0.0026561 | 0.0042983 | 0.24458 | 0.53661 |
| rs1929880 | A | C | 0.241854 | -0.0752505 | 0.016242 | 938.7017657 | 3.60E-06 | -0.0012037 | 0.0043091 | 0.75947 | 0.77998 |
| rs2598109 | T | C | 0.161047 | 0.0968641 | 0.018882 | 1146.617082 | 2.90E-07 | -0.003436 | 0.0050014 | 0.83753 | 0.49208 |
| rs2746117 | A | G | 0.589134 | 0.0646764 | 0.014127 | 915.3480543 | 4.69E-06 | 0.0034706 | 0.0037441 | 0.41061 | 0.35396 |
| rs28971325 | A | G | 0.766755 | 0.184128 | 0.016444 | 5537.4175 | 4.20E-29 | 0.0055739 | 0.0043643 | 0.23231 | 0.20155 |
| rs296724 | T | C | 0.227306 | -0.0793161 | 0.016577 | 999.0837228 | 1.71E-06 | -0.008741 | 0.0044104 | 0.77384 | 0.047493 |
| rs41271365 | C | T | 0.78792 | 0.0788342 | 0.016961 | 938.8886321 | 3.35E-06 | -0.0051105 | 0.0044993 | 0.21204 | 0.25603 |
| rs4959559 | G | A | 0.787804 | 0.0797863 | 0.017149 | 962.1377922 | 3.28E-06 | -0.0024054 | 0.0045545 | 0.21224 | 0.59739 |
| rs530125017 | C | T | 0.988891 | 0.356925 | 0.067372 | 1266.177434 | 1.17E-07 | 0.0077416 | 0.018206 | 0.010673 | 0.67068 |
| rs56248155 | G | C | 0.598251 | -0.0673744 | 0.014506 | 986.4536102 | 3.41E-06 | 0.0051329 | 0.0038456 | 0.59837 | 0.18196 |
| rs592294 | G | A | 0.446147 | -0.0676549 | 0.014273 | 1022.714569 | 2.14E-06 | -0.0022192 | 0.0037866 | 0.55448 | 0.55783 |
| rs61306957 | C | G | 0.987402 | 0.314039 | 0.062276 | 1109.505808 | 4.59E-07 | 0.0012562 | 0.016646 | 0.987602 | 0.93985 |
| rs61811976 | C | A | 0.914113 | -0.120568 | 0.024801 | 1032.009752 | 1.17E-06 | -0.001925 | 0.0065883 | 0.085915 | 0.77014 |
| rs62290785 | T | G | 0.975752 | 0.21287 | 0.045317 | 969.3407306 | 2.64E-06 | 0.0072366 | 0.011925 | 0.024621 | 0.54396 |
| rs6330 | A | G | 0.538728 | 0.0761419 | 0.013916 | 1303.548455 | 4.46E-08 | 0.0064282 | 0.0036937 | 0.46171 | 0.081803 |
| rs667828 | A | G | 0.609276 | 0.0648515 | 0.014223 | 905.0960201 | 5.13E-06 | 0.0093433 | 0.0037712 | 0.39073 | 0.013231 |
| rs6731576 | C | T | 0.204159 | 0.0802665 | 0.0173 | 946.3975719 | 3.49E-06 | -0.0053528 | 0.004589 | 0.79621 | 0.24344 |
| rs73346492 | C | T | 0.984211 | -0.25407 | 0.055643 | 906.8213084 | 4.97E-06 | -0.019986 | 0.014835 | 0.015625 | 0.17791 |
| rs79292884 | T | G | 0.986134 | 0.286788 | 0.059309 | 1016.920386 | 1.33E-06 | -0.0074619 | 0.015873 | 0.013669 | 0.63827 |
| rs9264971 | T | C | 0.308958 | 0.0702238 | 0.015308 | 951.8925927 | 4.48E-06 | -0.012353 | 0.0040689 | 0.692 | 0.0023985 |
| rs9929820 | T | C | 0.735945 | 0.0784603 | 0.015813 | 1081.882148 | 6.98E-07 | -0.00028702 | 0.0042209 | 0.26191 | 0.94579 |

Abbreviations: SE, standard error; EAF, effect allele frequency; SNP, single nucleotide polymorphism.

Supplement table 20. Summary data of SNPs used in MR analysis of IVs associated with frozen shoulder-LDL in females.

| **SNP** | **Effect Allele** | **Other Allele** | **EAF** | **Beta Estimate** | **SE** | **F-statistic** | **P.vale** | **beta. LDL** | **se. LDL** | **eaf. LDL** | **pval. LDL** |
| --- | --- | --- | --- | --- | --- | --- | --- | --- | --- | --- | --- |
| rs1042704 | A | G | 0.784999 | 0.103074 | 0.016909 | 1623.558928 | 1.09E-09 | -0.0016812 | 0.0039061 | 0.21535 | 0.6669 |
| rs111481541 | C | T | 0.971361 | -0.197512 | 0.042067 | 981.2260539 | 2.66E-06 | 0.0056982 | 0.009729 | 0.028411 | 0.55808 |
| rs117557731 | G | A | 0.963514 | 0.170286 | 0.037039 | 921.5702453 | 4.28E-06 | 0.0021411 | 0.0085241 | 0.036593 | 0.80167 |
| rs12915503 | C | T | 0.745019 | 0.0862784 | 0.0161 | 1279.408637 | 8.38E-08 | -0.008671 | 0.0038241 | 0.25366 | 0.023365 |
| rs13107325 | T | C | 0.925314 | 0.134162 | 0.026431 | 1125.042955 | 3.85E-07 | -0.018264 | 0.006139 | 0.073708 | 0.0029304 |
| rs13151266 | A | C | 0.755331 | -0.0773496 | 0.016169 | 999.7554641 | 1.72E-06 | -0.00068605 | 0.00373 | 0.2451 | 0.85407 |
| rs1929880 | A | C | 0.241854 | -0.0752505 | 0.016242 | 938.7017657 | 3.60E-06 | 0.0056993 | 0.0037648 | 0.76015 | 0.13007 |
| rs2598109 | T | C | 0.161047 | 0.0968641 | 0.018882 | 1146.617082 | 2.90E-07 | -0.0023809 | 0.0043534 | 0.8389 | 0.58443 |
| rs2746117 | A | G | 0.589134 | 0.0646764 | 0.014127 | 915.3480543 | 4.69E-06 | 0.0029917 | 0.003266 | 0.41104 | 0.35967 |
| rs28971325 | A | G | 0.766755 | 0.184128 | 0.016444 | 5537.4175 | 4.20E-29 | -0.0067003 | 0.0037955 | 0.23358 | 0.077512 |
| rs296724 | T | C | 0.227306 | -0.0793161 | 0.016577 | 999.0837228 | 1.71E-06 | -0.0052997 | 0.0038318 | 0.77321 | 0.16664 |
| rs41271365 | C | T | 0.78792 | 0.0788342 | 0.016961 | 938.8886321 | 3.35E-06 | -0.0013882 | 0.0039188 | 0.21188 | 0.72315 |
| rs4959559 | G | A | 0.787804 | 0.0797863 | 0.017149 | 962.1377922 | 3.28E-06 | 0.0024748 | 0.0039676 | 0.21109 | 0.53279 |
| rs530125017 | C | T | 0.988891 | 0.356925 | 0.067372 | 1266.177434 | 1.17E-07 | -0.01128 | 0.015435 | 0.011296 | 0.4649 |
| rs56248155 | G | C | 0.598251 | -0.0673744 | 0.014506 | 986.4536102 | 3.41E-06 | 0.0016687 | 0.0033522 | 0.59869 | 0.61863 |
| rs592294 | G | A | 0.446147 | -0.0676549 | 0.014273 | 1022.714569 | 2.14E-06 | -0.0049539 | 0.0032989 | 0.55312 | 0.13317 |
| rs61306957 | C | G | 0.987402 | 0.314039 | 0.062276 | 1109.505808 | 4.59E-07 | 0.01456 | 0.014569 | 0.987725 | 0.31761 |
| rs61811976 | C | A | 0.914113 | -0.120568 | 0.024801 | 1032.009752 | 1.17E-06 | 0.00043921 | 0.0057055 | 0.086482 | 0.93864 |
| rs62290785 | T | G | 0.975752 | 0.21287 | 0.045317 | 969.3407306 | 2.64E-06 | -0.0062881 | 0.010519 | 0.024022 | 0.55 |
| rs6330 | A | G | 0.538728 | 0.0761419 | 0.013916 | 1303.548455 | 4.46E-08 | 2.57E-05 | 0.003216 | 0.46198 | 0.99364 |
| rs667828 | A | G | 0.609276 | 0.0648515 | 0.014223 | 905.0960201 | 5.13E-06 | -0.00092678 | 0.0032876 | 0.3908 | 0.77802 |
| rs6731576 | C | T | 0.204159 | 0.0802665 | 0.0173 | 946.3975719 | 3.49E-06 | -0.0014934 | 0.0040069 | 0.7964 | 0.70936 |
| rs7117115 | G | A | 0.561971 | -0.0658211 | 0.014145 | 964.2158924 | 3.27E-06 | -0.0017181 | 0.00327 | 0.43789 | 0.5993 |
| rs73346492 | C | T | 0.984211 | -0.25407 | 0.055643 | 906.8213084 | 4.97E-06 | -0.0031567 | 0.012962 | 0.015515 | 0.80759 |
| rs79292884 | T | G | 0.986134 | 0.286788 | 0.059309 | 1016.920386 | 1.33E-06 | -0.016149 | 0.013649 | 0.013953 | 0.23674 |
| rs9264971 | T | C | 0.308958 | 0.0702238 | 0.015308 | 951.8925927 | 4.48E-06 | 0.0030323 | 0.0035326 | 0.69008 | 0.39068 |
| rs9929820 | T | C | 0.735945 | 0.0784603 | 0.015813 | 1081.882148 | 6.98E-07 | -0.002877 | 0.0036442 | 0.26392 | 0.42983 |

Abbreviations: SE, standard error; EAF, effect allele frequency; SNP, single nucleotide polymorphism.

Supplement table 21. Summary data of SNPs used in MR analysis of IVs associated with frozen shoulder-LDL in males.

| **SNP** | **Effect Allele** | **Other Allele** | **EAF** | **Beta Estimate** | **SE** | **F-statistic** | **P.vale** | **beta. LDL** | **se. LDL** | **eaf. LDL** | **pval. LDL** |
| --- | --- | --- | --- | --- | --- | --- | --- | --- | --- | --- | --- |
| rs1042704 | A | G | 0.784999 | 0.103074 | 0.016909 | 1623.558928 | 1.09E-09 | -0.0073589 | 0.0042511 | 0.21556 | 0.083442 |
| rs111481541 | C | T | 0.971361 | -0.197512 | 0.042067 | 981.2260539 | 2.66E-06 | 0.00069015 | 0.010576 | 0.02889 | 0.94797 |
| rs117557731 | G | A | 0.963514 | 0.170286 | 0.037039 | 921.5702453 | 4.28E-06 | 0.0018026 | 0.0093467 | 0.036518 | 0.84707 |
| rs12915503 | C | T | 0.745019 | 0.0862784 | 0.0161 | 1279.408637 | 8.38E-08 | -0.0019635 | 0.0041693 | 0.25261 | 0.63769 |
| rs13107325 | T | C | 0.925314 | 0.134162 | 0.026431 | 1125.042955 | 3.85E-07 | -0.030088 | 0.0066072 | 0.076162 | 5.27E-06 |
| rs13151266 | A | C | 0.755331 | -0.0773496 | 0.016169 | 999.7554641 | 1.72E-06 | 0.00276 | 0.0040803 | 0.24439 | 0.49877 |
| rs1929880 | A | C | 0.241854 | -0.0752505 | 0.016242 | 938.7017657 | 3.60E-06 | 0.004024 | 0.0040889 | 0.75916 | 0.32506 |
| rs2598109 | T | C | 0.161047 | 0.0968641 | 0.018882 | 1146.617082 | 2.90E-07 | -0.0059728 | 0.0047467 | 0.83774 | 0.20828 |
| rs2746117 | A | G | 0.589134 | 0.0646764 | 0.014127 | 915.3480543 | 4.69E-06 | 0.00074082 | 0.0035544 | 0.41027 | 0.8349 |
| rs28971325 | A | G | 0.766755 | 0.184128 | 0.016444 | 5537.4175 | 4.20E-29 | -0.0073149 | 0.0041437 | 0.23242 | 0.077514 |
| rs296724 | T | C | 0.227306 | -0.0793161 | 0.016577 | 999.0837228 | 1.71E-06 | -0.0037102 | 0.0041866 | 0.77398 | 0.37551 |
| rs41271365 | C | T | 0.78792 | 0.0788342 | 0.016961 | 938.8886321 | 3.35E-06 | -0.00052938 | 0.00427 | 0.21195 | 0.90133 |
| rs4959559 | G | A | 0.787804 | 0.0797863 | 0.017149 | 962.1377922 | 3.28E-06 | -0.0012606 | 0.0043239 | 0.21211 | 0.77063 |
| rs530125017 | C | T | 0.988891 | 0.356925 | 0.067372 | 1266.177434 | 1.17E-07 | 0.003604 | 0.017238 | 0.010733 | 0.83439 |
| rs56248155 | G | C | 0.598251 | -0.0673744 | 0.014506 | 986.4536102 | 3.41E-06 | 0.0030846 | 0.0036503 | 0.59835 | 0.3981 |
| rs592294 | G | A | 0.446147 | -0.0676549 | 0.014273 | 1022.714569 | 2.14E-06 | 0.0025193 | 0.0035944 | 0.55483 | 0.48337 |
| rs61306957 | C | G | 0.987402 | 0.314039 | 0.062276 | 1109.505808 | 4.59E-07 | -0.018012 | 0.015751 | 0.987526 | 0.25281 |
| rs61811976 | C | A | 0.914113 | -0.120568 | 0.024801 | 1032.009752 | 1.17E-06 | 0.00073939 | 0.0062515 | 0.085964 | 0.90585 |
| rs62290785 | T | G | 0.975752 | 0.21287 | 0.045317 | 969.3407306 | 2.64E-06 | 0.013119 | 0.011325 | 0.02457 | 0.2467 |
| rs6330 | A | G | 0.538728 | 0.0761419 | 0.013916 | 1303.548455 | 4.46E-08 | 0.0017829 | 0.0035056 | 0.46144 | 0.61104 |
| rs667828 | A | G | 0.609276 | 0.0648515 | 0.014223 | 905.0960201 | 5.13E-06 | 0.0048477 | 0.0035794 | 0.39061 | 0.17563 |
| rs6731576 | C | T | 0.204159 | 0.0802665 | 0.0173 | 946.3975719 | 3.49E-06 | -0.00087637 | 0.0043532 | 0.79595 | 0.84045 |
| rs7117115 | G | A | 0.561971 | -0.0658211 | 0.014145 | 964.2158924 | 3.27E-06 | 0.0024799 | 0.0035651 | 0.43738 | 0.48668 |
| rs73346492 | C | T | 0.984211 | -0.25407 | 0.055643 | 906.8213084 | 4.97E-06 | -0.0076642 | 0.014084 | 0.015614 | 0.58633 |
| rs79292884 | T | G | 0.986134 | 0.286788 | 0.059309 | 1016.920386 | 1.33E-06 | -0.0057053 | 0.015066 | 0.01366 | 0.70491 |
| rs9264971 | T | C | 0.308958 | 0.0702238 | 0.015308 | 951.8925927 | 4.48E-06 | -0.00058614 | 0.0038593 | 0.6916 | 0.87929 |
| rs9929820 | T | C | 0.735945 | 0.0784603 | 0.015813 | 1081.882148 | 6.98E-07 | -0.003017 | 0.0040032 | 0.26221 | 0.45106 |

Abbreviations: SE, standard error; EAF, effect allele frequency; SNP, single nucleotide polymorphism.

Supplement table 22. Summary data of SNPs used in MR analysis of IVs associated with frozen shoulder-TG in females.

| **SNP** | **Effect Allele** | **Other Allele** | **EAF** | **Beta Estimate** | **SE** | **F-statistic** | **P.vale** | **beta. TG** | **se. TG** | **eaf. TG** | **pval. TG** |
| --- | --- | --- | --- | --- | --- | --- | --- | --- | --- | --- | --- |
| rs1042704 | A | G | 0.784999 | 0.103074 | 0.016909 | 1623.558928 | 1.09E-09 | -0.0079883 | 0.0038859 | 0.21534 | 0.039812 |
| rs111481541 | C | T | 0.971361 | -0.197512 | 0.042067 | 981.2260539 | 2.66E-06 | -0.0092667 | 0.0096798 | 0.028401 | 0.33841 |
| rs117557731 | G | A | 0.963514 | 0.170286 | 0.037039 | 921.5702453 | 4.28E-06 | 0.0024551 | 0.0084816 | 0.036581 | 0.77222 |
| rs12915503 | C | T | 0.745019 | 0.0862784 | 0.0161 | 1279.408637 | 8.38E-08 | 0.0048677 | 0.0038046 | 0.25361 | 0.20075 |
| rs13151266 | A | C | 0.755331 | -0.0773496 | 0.016169 | 999.7554641 | 1.72E-06 | -0.00059125 | 0.0037107 | 0.2451 | 0.8734 |
| rs1929880 | A | C | 0.241854 | -0.0752505 | 0.016242 | 938.7017657 | 3.60E-06 | -0.0028292 | 0.0037447 | 0.76012 | 0.44994 |
| rs2598109 | T | C | 0.161047 | 0.0968641 | 0.018882 | 1146.617082 | 2.90E-07 | -0.0053183 | 0.004331 | 0.83891 | 0.21945 |
| rs2746117 | A | G | 0.589134 | 0.0646764 | 0.014127 | 915.3480543 | 4.69E-06 | -0.00087213 | 0.0032492 | 0.41102 | 0.78838 |
| rs28971325 | A | G | 0.766755 | 0.184128 | 0.016444 | 5537.4175 | 4.20E-29 | -0.0027846 | 0.0037758 | 0.23356 | 0.46083 |
| rs296724 | T | C | 0.227306 | -0.0793161 | 0.016577 | 999.0837228 | 1.71E-06 | 0.0015054 | 0.0038118 | 0.77321 | 0.6929 |
| rs41271365 | C | T | 0.78792 | 0.0788342 | 0.016961 | 938.8886321 | 3.35E-06 | -0.0033251 | 0.0038986 | 0.21183 | 0.39372 |
| rs4959559 | G | A | 0.787804 | 0.0797863 | 0.017149 | 962.1377922 | 3.28E-06 | 0.0018871 | 0.0039465 | 0.21109 | 0.63252 |
| rs530125017 | C | T | 0.988891 | 0.356925 | 0.067372 | 1266.177434 | 1.17E-07 | -0.00068655 | 0.015351 | 0.0113 | 0.96433 |
| rs56248155 | G | C | 0.598251 | -0.0673744 | 0.014506 | 986.4536102 | 3.41E-06 | -0.0020001 | 0.0033345 | 0.59874 | 0.54862 |
| rs592294 | G | A | 0.446147 | -0.0676549 | 0.014273 | 1022.714569 | 2.14E-06 | -0.0047305 | 0.0032818 | 0.55312 | 0.14946 |
| rs61306957 | C | G | 0.987402 | 0.314039 | 0.062276 | 1109.505808 | 4.59E-07 | -0.012346 | 0.014496 | 0.98773 | 0.39436 |
| rs61811976 | C | A | 0.914113 | -0.120568 | 0.024801 | 1032.009752 | 1.17E-06 | 0.0019335 | 0.0056765 | 0.086461 | 0.73339 |
| rs62290785 | T | G | 0.975752 | 0.21287 | 0.045317 | 969.3407306 | 2.64E-06 | 0.003302 | 0.010467 | 0.024011 | 0.7524 |
| rs6330 | A | G | 0.538728 | 0.0761419 | 0.013916 | 1303.548455 | 4.46E-08 | -0.0039816 | 0.0031991 | 0.46194 | 0.21327 |
| rs667828 | A | G | 0.609276 | 0.0648515 | 0.014223 | 905.0960201 | 5.13E-06 | 0.0049469 | 0.0032708 | 0.39081 | 0.13041 |
| rs6731576 | C | T | 0.204159 | 0.0802665 | 0.0173 | 946.3975719 | 3.49E-06 | -0.0047594 | 0.0039862 | 0.79642 | 0.23249 |
| rs73346492 | C | T | 0.984211 | -0.25407 | 0.055643 | 906.8213084 | 4.97E-06 | 0.023104 | 0.012899 | 0.015504 | 0.073271 |
| rs79292884 | T | G | 0.986134 | 0.286788 | 0.059309 | 1016.920386 | 1.33E-06 | -0.007834 | 0.013579 | 0.013949 | 0.56401 |
| rs9264971 | T | C | 0.308958 | 0.0702238 | 0.015308 | 951.8925927 | 4.48E-06 | 0.010663 | 0.0035141 | 0.6901 | 0.0024122 |
| rs9929820 | T | C | 0.735945 | 0.0784603 | 0.015813 | 1081.882148 | 6.98E-07 | 0.0026431 | 0.0036248 | 0.26395 | 0.46589 |

Abbreviations: SE, standard error; EAF, effect allele frequency; SNP, single nucleotide polymorphism.

Supplement table 23. Summary data of SNPs used in MR analysis of IVs associated with frozen shoulder-TG in males.

| **SNP** | **Effect Allele** | **Other Allele** | **EAF** | **Beta Estimate** | **SE** | **F-statistic** | **P.vale** | **beta. TG** | **se. TG** | **eaf. TG** | **pval. TG** |
| --- | --- | --- | --- | --- | --- | --- | --- | --- | --- | --- | --- |
| rs1042704 | A | G | 0.784999 | 0.103074 | 0.016909 | 1623.558928 | 1.09E-09 | -0.0060818 | 0.0043072 | 0.21555 | 0.15795 |
| rs111481541 | C | T | 0.971361 | -0.197512 | 0.042067 | 981.2260539 | 2.66E-06 | -0.0057639 | 0.010718 | 0.028883 | 0.59072 |
| rs117557731 | G | A | 0.963514 | 0.170286 | 0.037039 | 921.5702453 | 4.28E-06 | -0.0013313 | 0.0094653 | 0.036556 | 0.88815 |
| rs12915503 | C | T | 0.745019 | 0.0862784 | 0.0161 | 1279.408637 | 8.38E-08 | 0.0071342 | 0.0042239 | 0.25262 | 0.091218 |
| rs13151266 | A | C | 0.755331 | -0.0773496 | 0.016169 | 999.7554641 | 1.72E-06 | -0.00082662 | 0.0041337 | 0.24442 | 0.8415 |
| rs1929880 | A | C | 0.241854 | -0.0752505 | 0.016242 | 938.7017657 | 3.60E-06 | 0.0030195 | 0.0041423 | 0.75918 | 0.46604 |
| rs2598109 | T | C | 0.161047 | 0.0968641 | 0.018882 | 1146.617082 | 2.90E-07 | -0.0034345 | 0.004808 | 0.8377 | 0.47503 |
| rs2746117 | A | G | 0.589134 | 0.0646764 | 0.014127 | 915.3480543 | 4.69E-06 | -0.004375 | 0.0036006 | 0.41033 | 0.22433 |
| rs28971325 | A | G | 0.766755 | 0.184128 | 0.016444 | 5537.4175 | 4.20E-29 | -0.0032921 | 0.0041979 | 0.23244 | 0.4329 |
| rs296724 | T | C | 0.227306 | -0.0793161 | 0.016577 | 999.0837228 | 1.71E-06 | -0.0069749 | 0.0042418 | 0.774 | 0.10012 |
| rs41271365 | C | T | 0.78792 | 0.0788342 | 0.016961 | 938.8886321 | 3.35E-06 | -0.001713 | 0.0043265 | 0.21195 | 0.69215 |
| rs4959559 | G | A | 0.787804 | 0.0797863 | 0.017149 | 962.1377922 | 3.28E-06 | 0.0085617 | 0.0043805 | 0.21209 | 0.050645 |
| rs530125017 | C | T | 0.988891 | 0.356925 | 0.067372 | 1266.177434 | 1.17E-07 | -0.025272 | 0.017458 | 0.01074 | 0.14774 |
| rs56248155 | G | C | 0.598251 | -0.0673744 | 0.014506 | 986.4536102 | 3.41E-06 | -0.00095145 | 0.0036983 | 0.59836 | 0.79697 |
| rs592294 | G | A | 0.446147 | -0.0676549 | 0.014273 | 1022.714569 | 2.14E-06 | 0.0027087 | 0.0036418 | 0.55476 | 0.45701 |
| rs61306957 | C | G | 0.987402 | 0.314039 | 0.062276 | 1109.505808 | 4.59E-07 | -0.0020527 | 0.01596 | 0.98753 | 0.89767 |
| rs61811976 | C | A | 0.914113 | -0.120568 | 0.024801 | 1032.009752 | 1.17E-06 | 0.00028736 | 0.0063338 | 0.085952 | 0.96381 |
| rs62290785 | T | G | 0.975752 | 0.21287 | 0.045317 | 969.3407306 | 2.64E-06 | 0.0074635 | 0.011475 | 0.024565 | 0.51543 |
| rs6330 | A | G | 0.538728 | 0.0761419 | 0.013916 | 1303.548455 | 4.46E-08 | 0.0033253 | 0.0035515 | 0.46145 | 0.34912 |
| rs667828 | A | G | 0.609276 | 0.0648515 | 0.014223 | 905.0960201 | 5.13E-06 | -0.0012851 | 0.0036265 | 0.39071 | 0.72307 |
| rs6731576 | C | T | 0.204159 | 0.0802665 | 0.0173 | 946.3975719 | 3.49E-06 | -0.0034758 | 0.004411 | 0.79599 | 0.43071 |
| rs73346492 | C | T | 0.984211 | -0.25407 | 0.055643 | 906.8213084 | 4.97E-06 | -0.0041869 | 0.014263 | 0.015628 | 0.76911 |
| rs79292884 | T | G | 0.986134 | 0.286788 | 0.059309 | 1016.920386 | 1.33E-06 | -0.02197 | 0.015255 | 0.013676 | 0.14982 |
| rs9264971 | T | C | 0.308958 | 0.0702238 | 0.015308 | 951.8925927 | 4.48E-06 | 0.0066728 | 0.0039102 | 0.69161 | 0.087913 |
| rs9929820 | T | C | 0.735945 | 0.0784603 | 0.015813 | 1081.882148 | 6.98E-07 | 0.0037044 | 0.0040555 | 0.26223 | 0.36102 |

Abbreviations: SE, standard error; EAF, effect allele frequency; SNP, single nucleotide polymorphism

Supplement figure 1: MR analysis of serum lipids and frozen shoulder without adjusting for confounders.


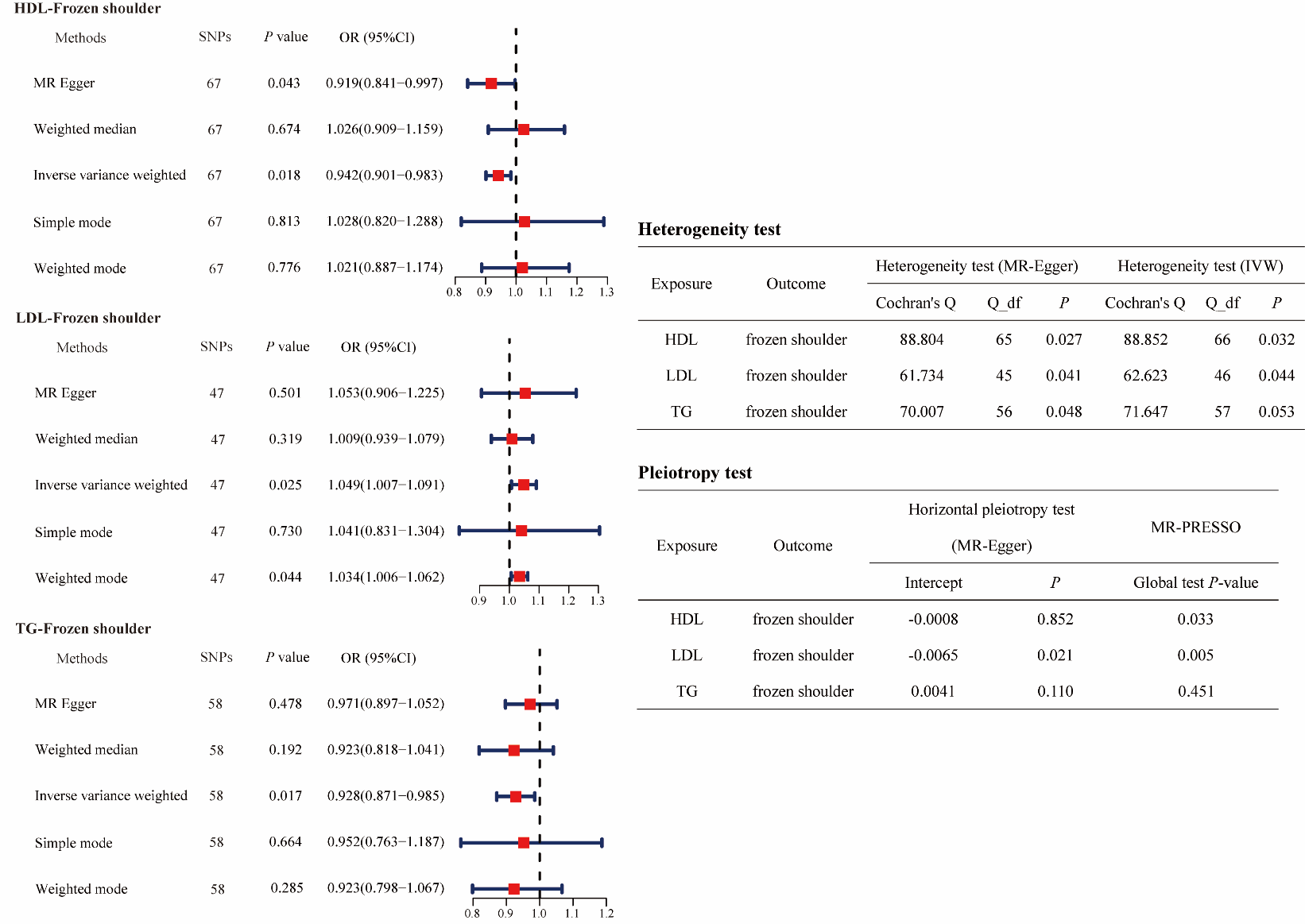


Supplement figure 2. Scatter plots of the causal effects.


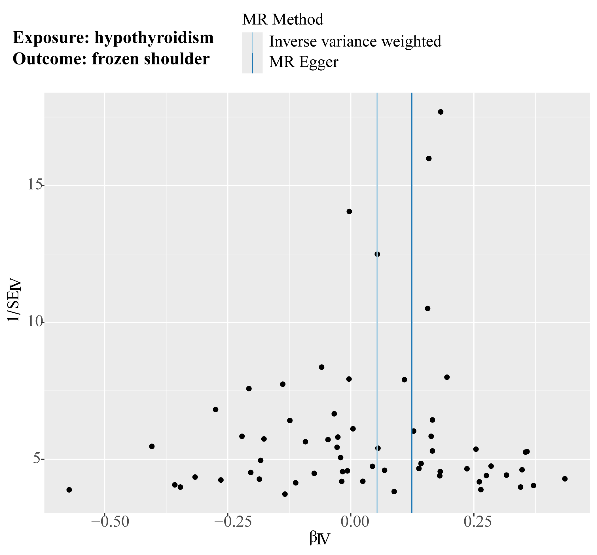

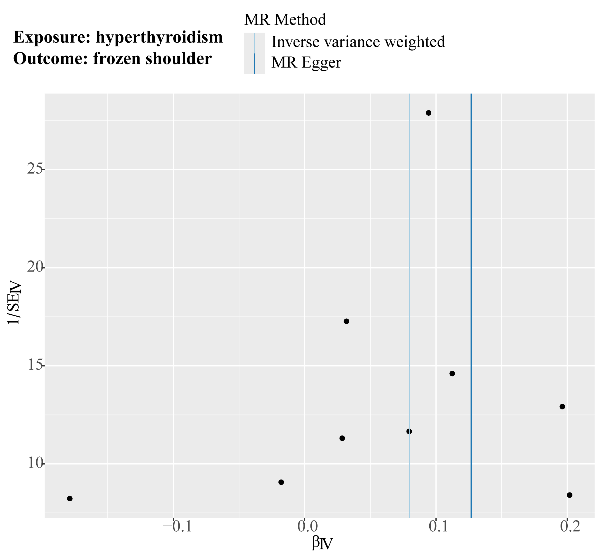

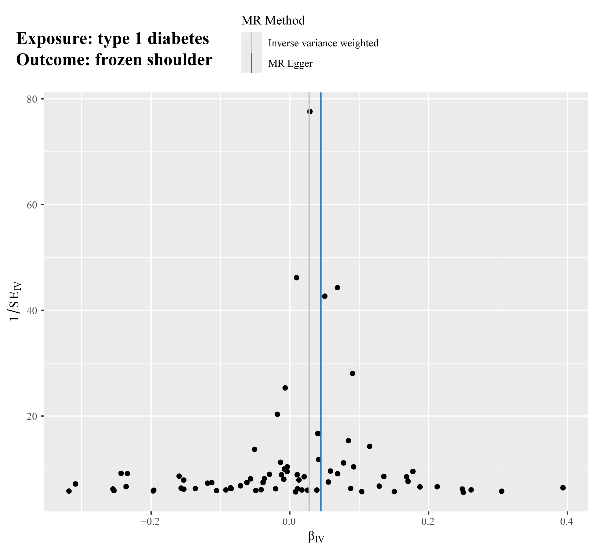


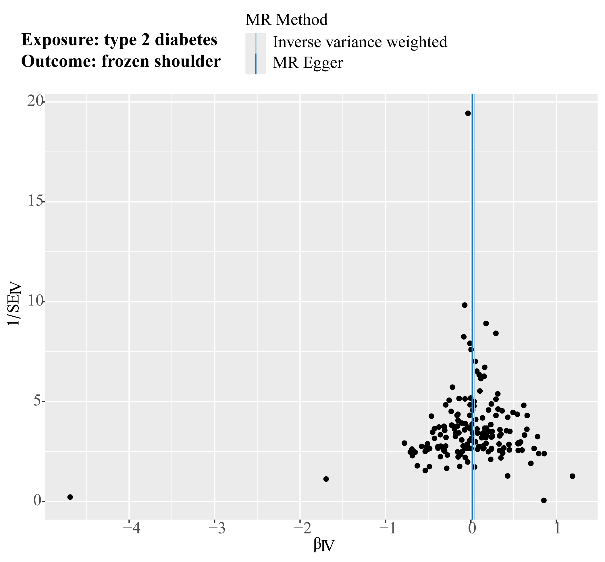

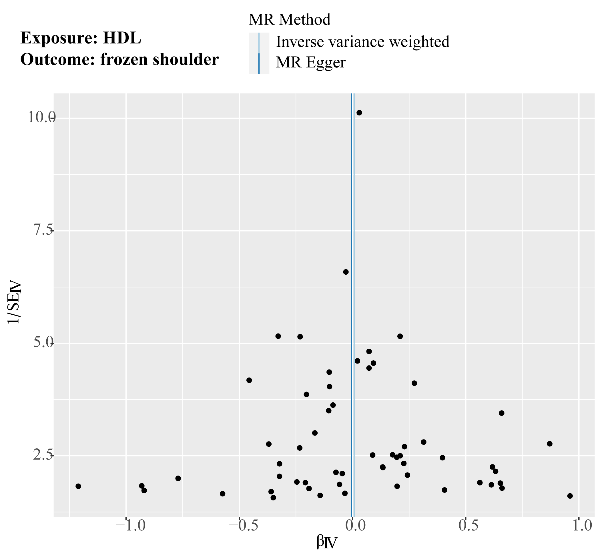

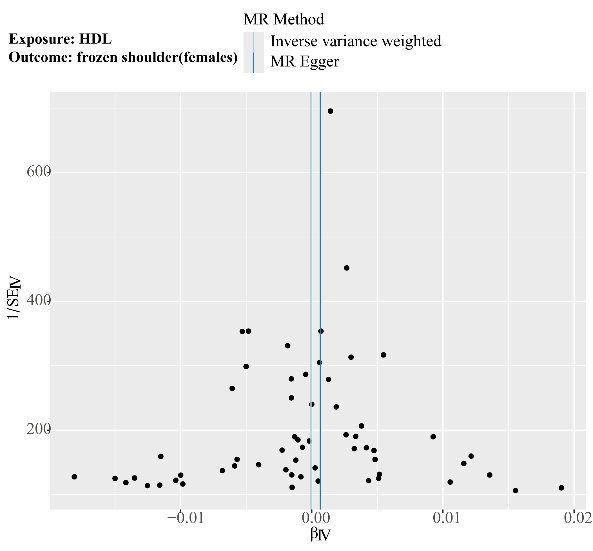


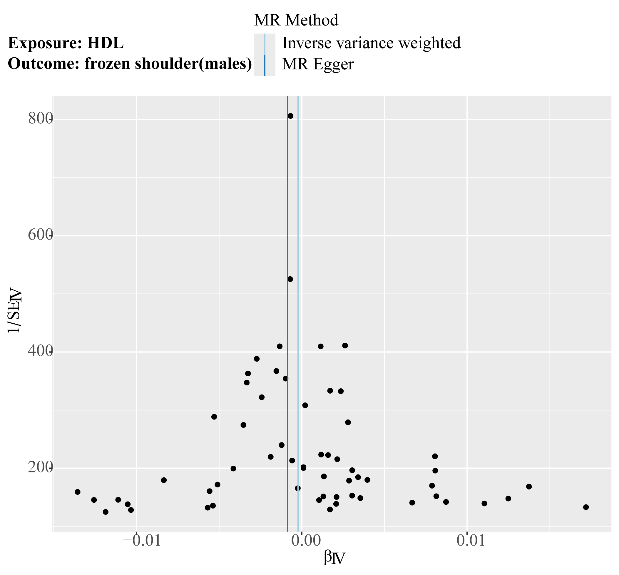

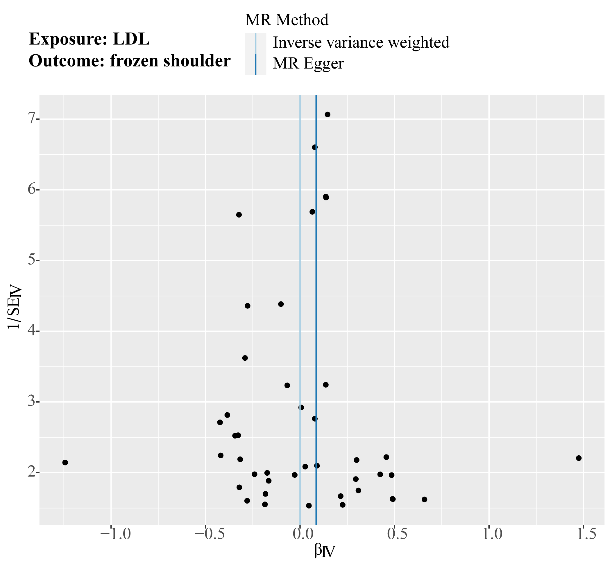

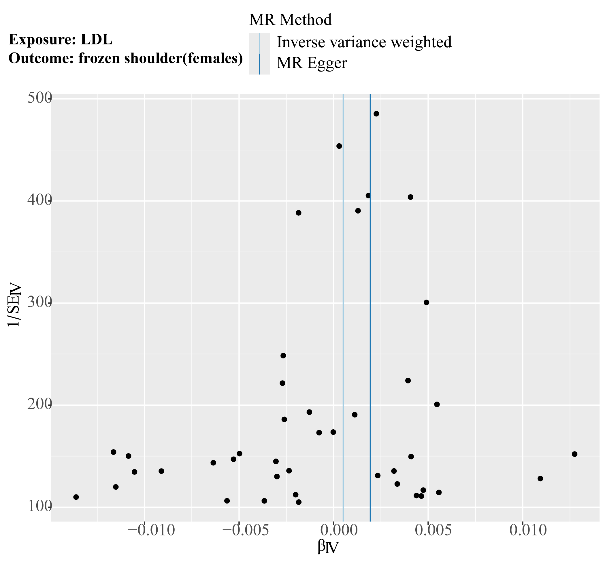

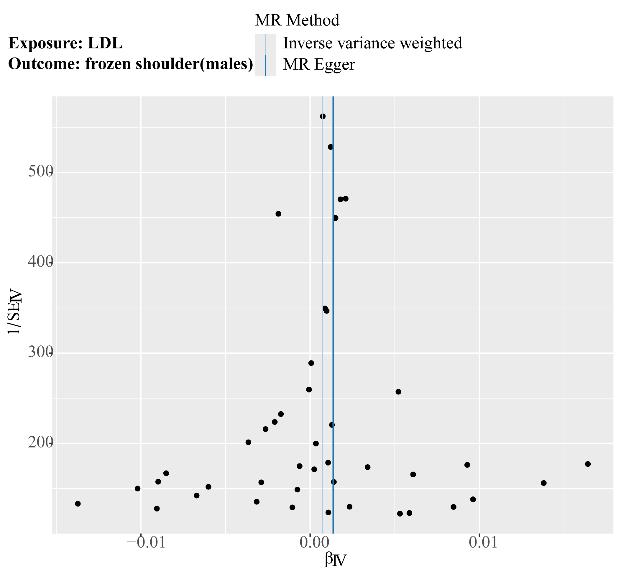

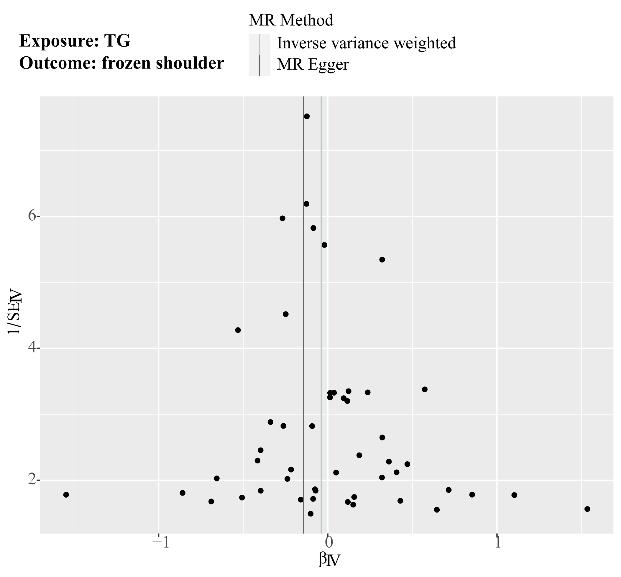

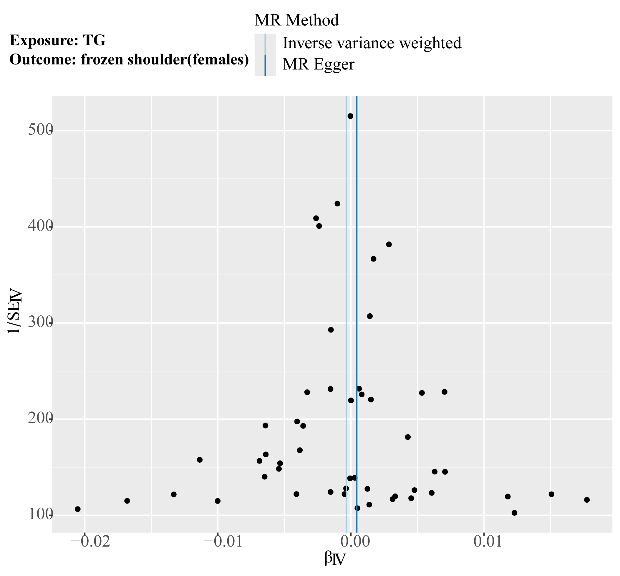


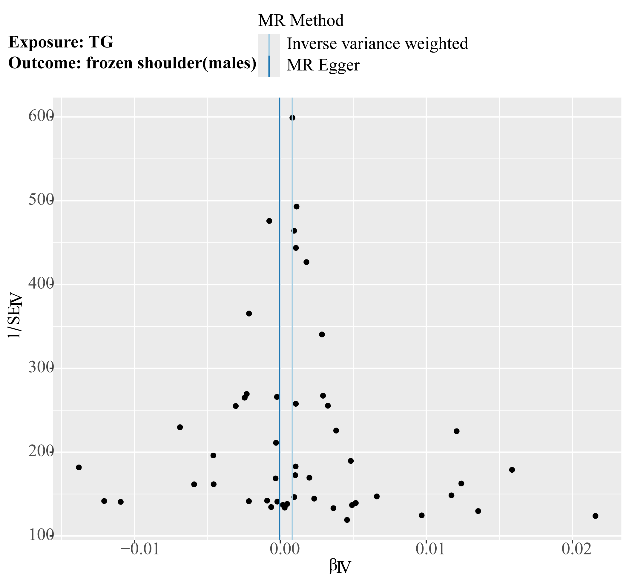

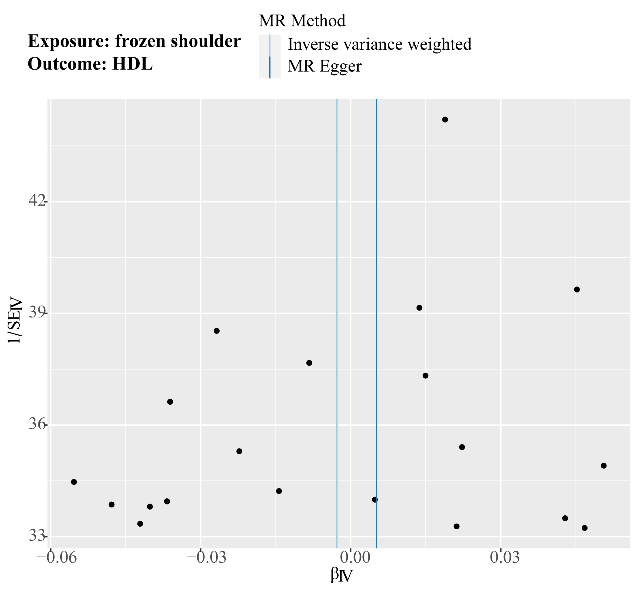

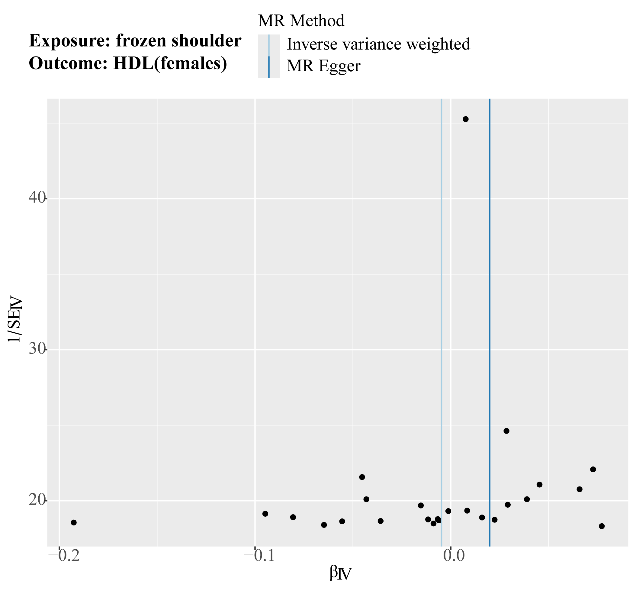


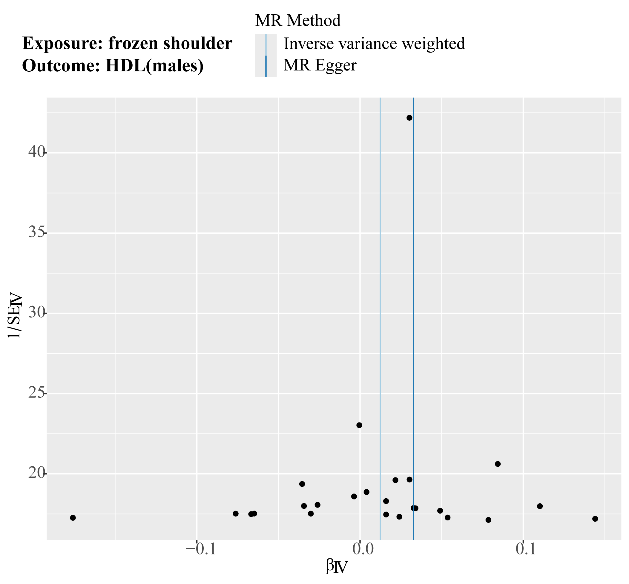

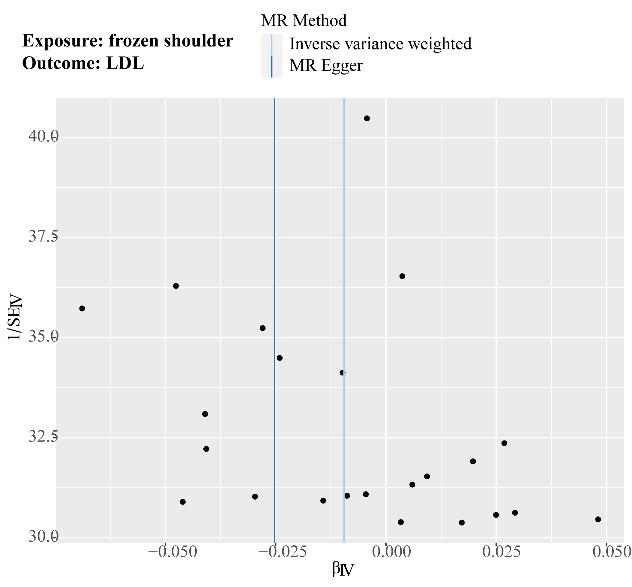

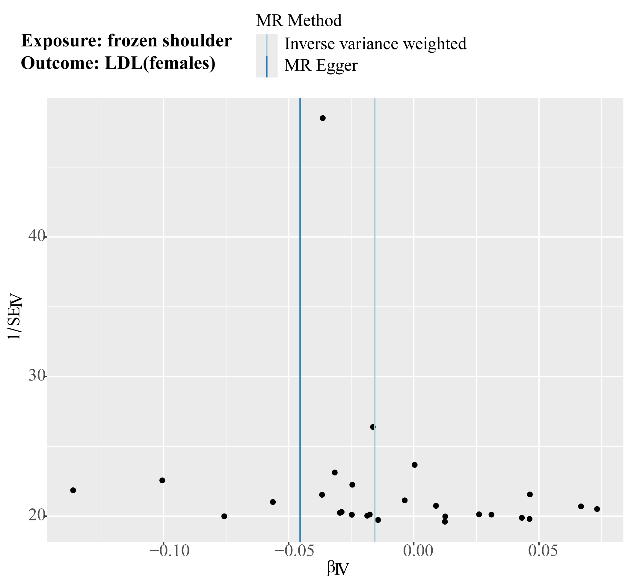


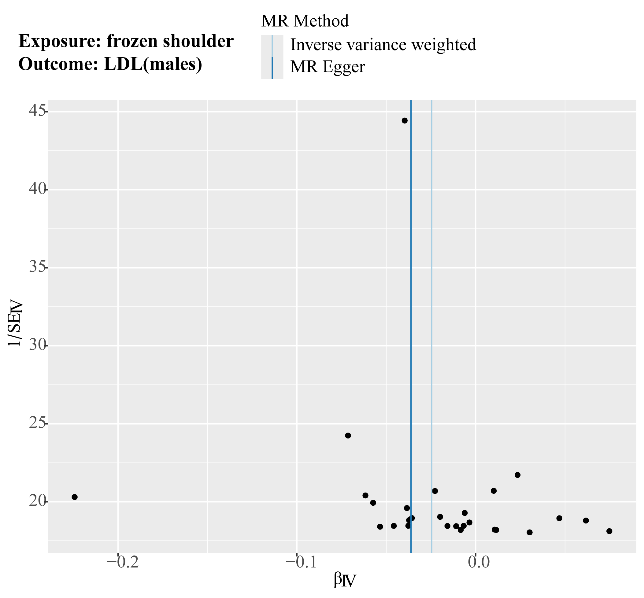

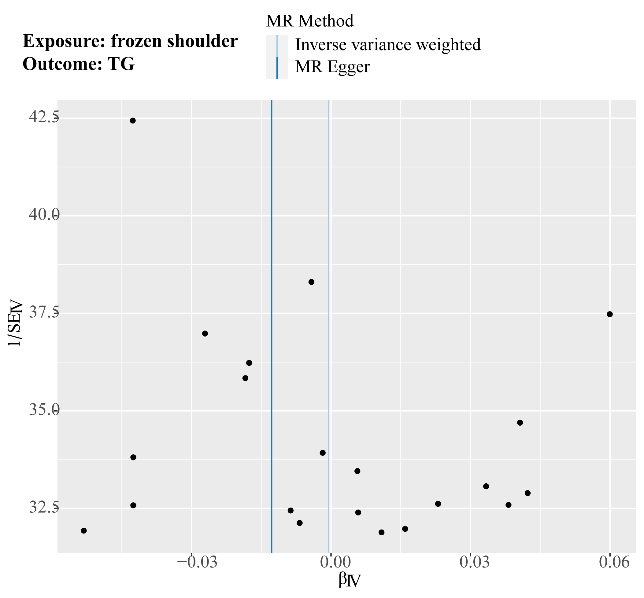

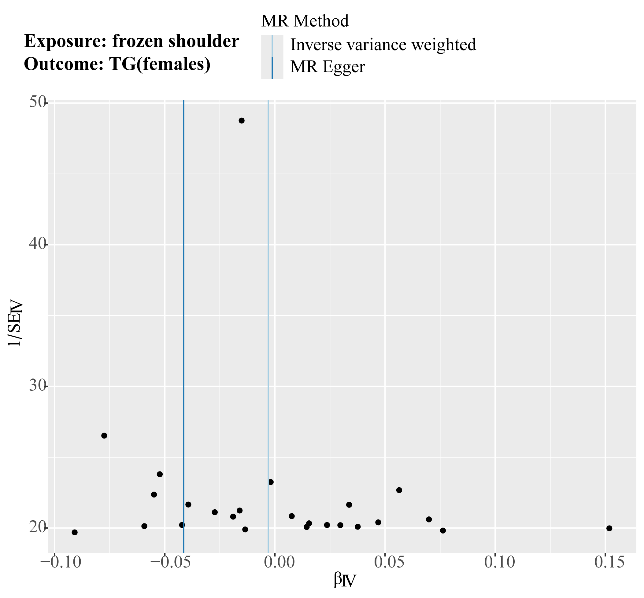


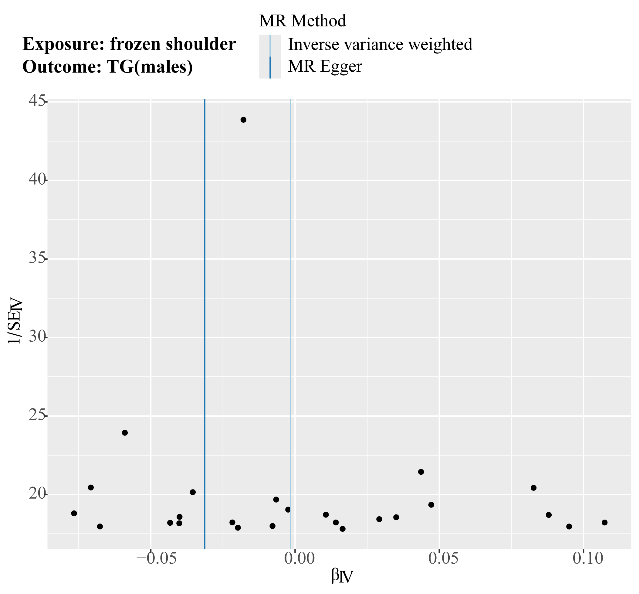


Supplement figure 3. Leave-one-out sensitivity analysis.


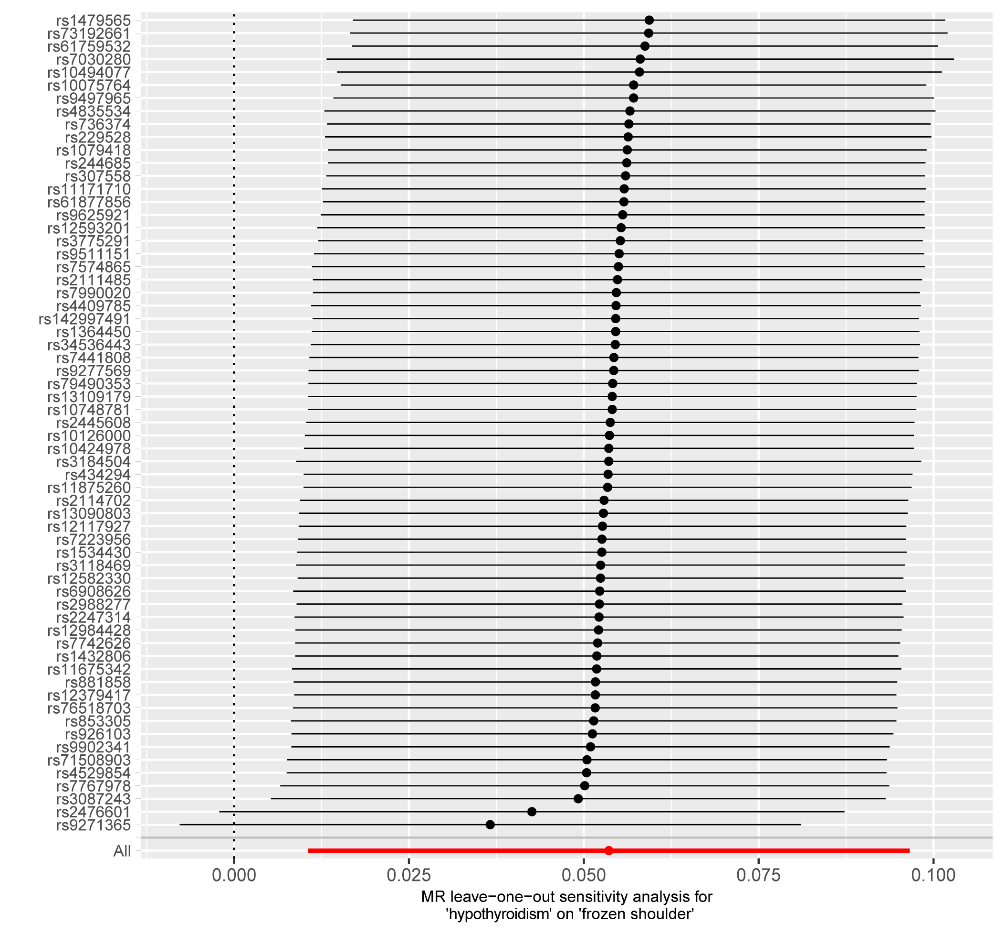

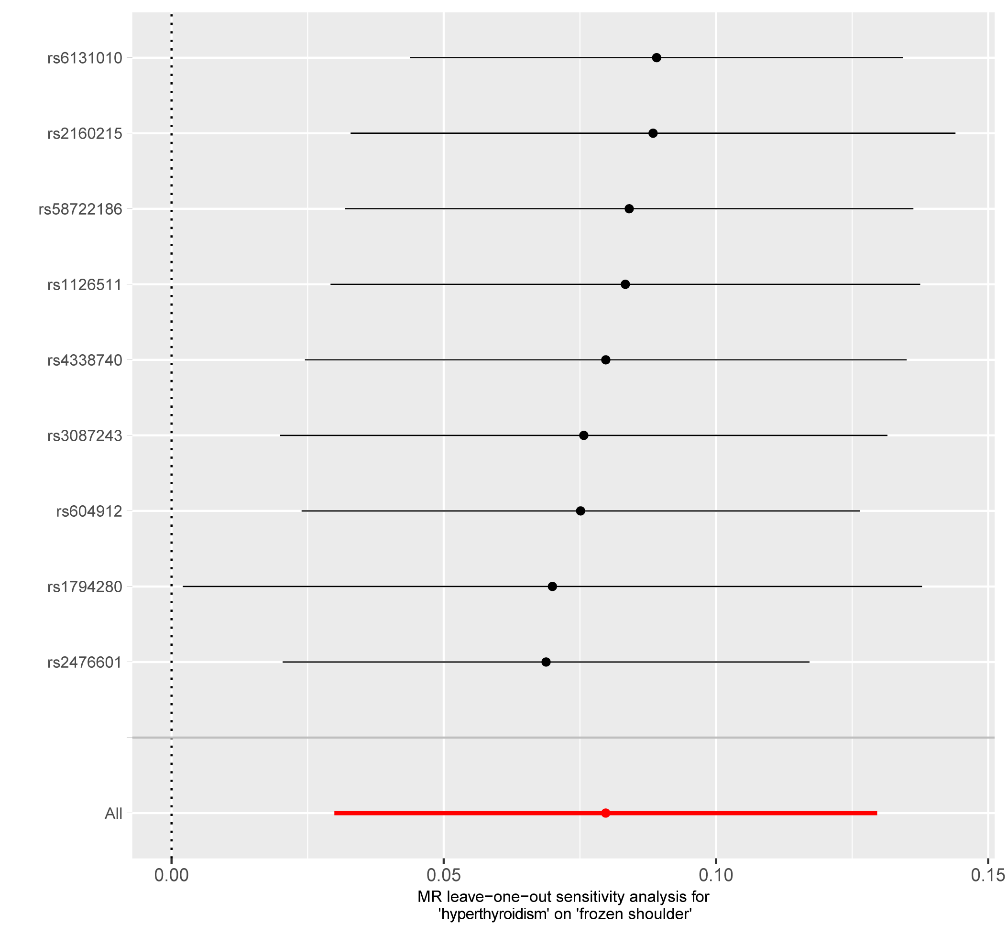


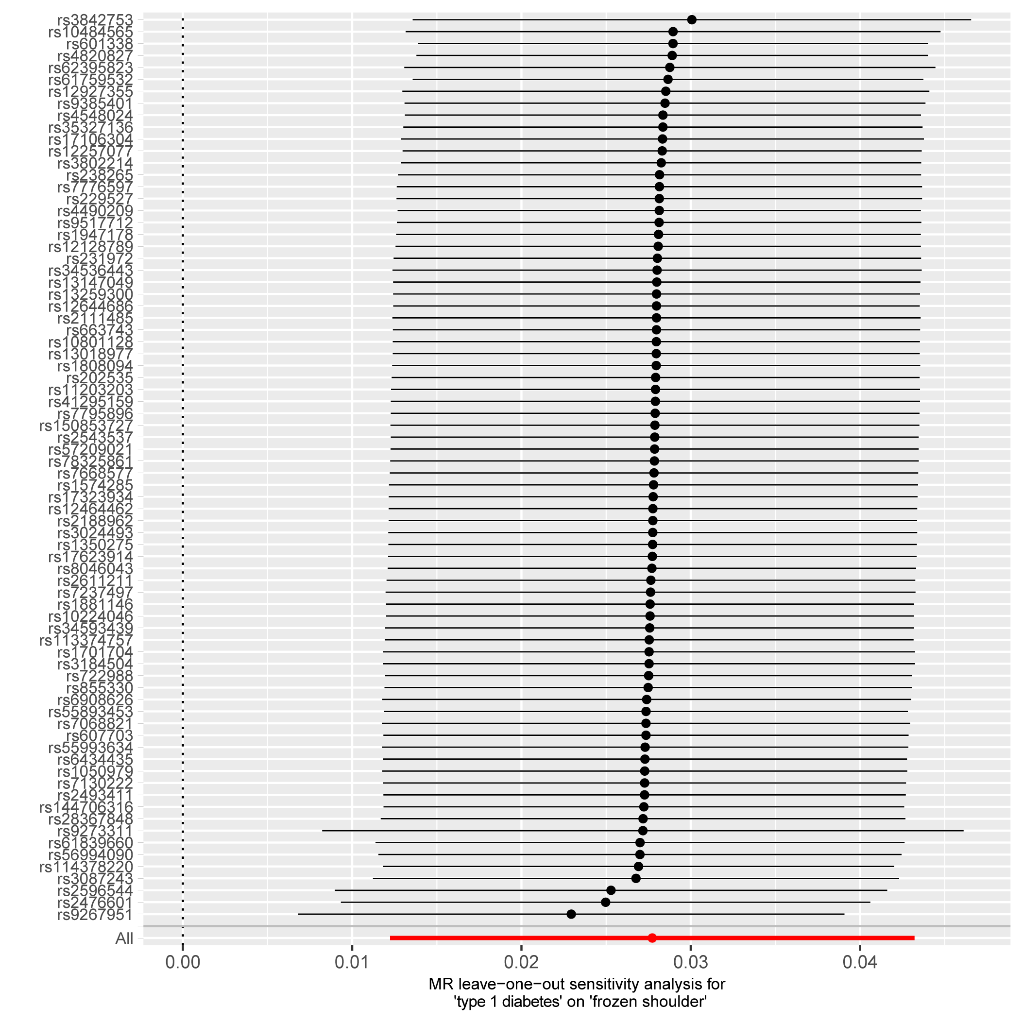

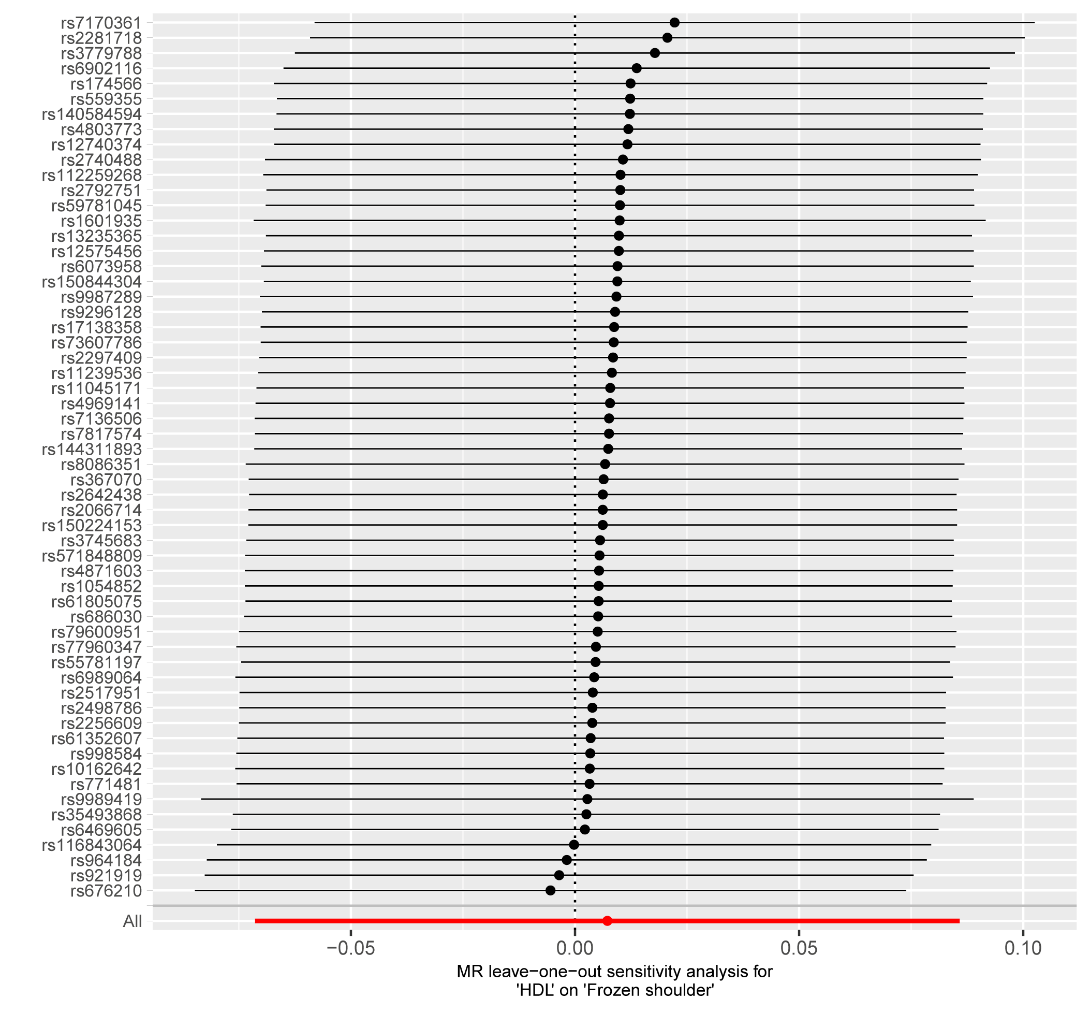


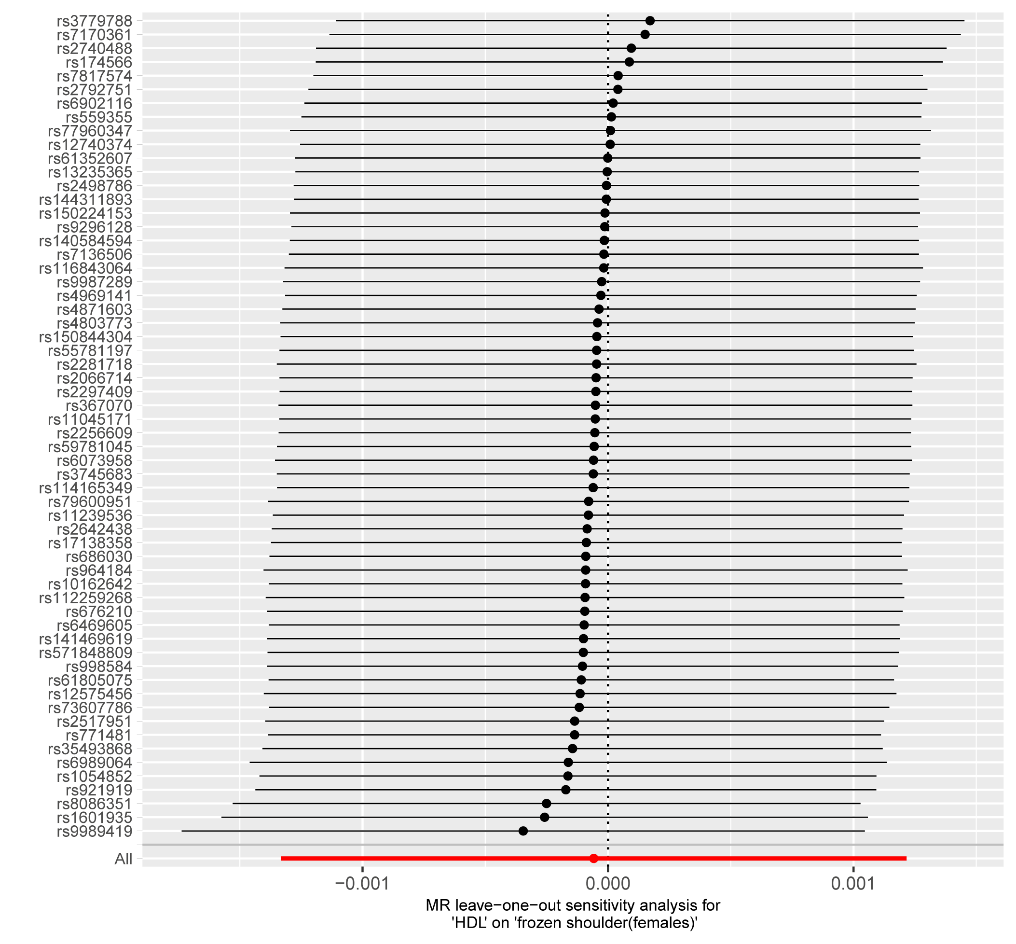

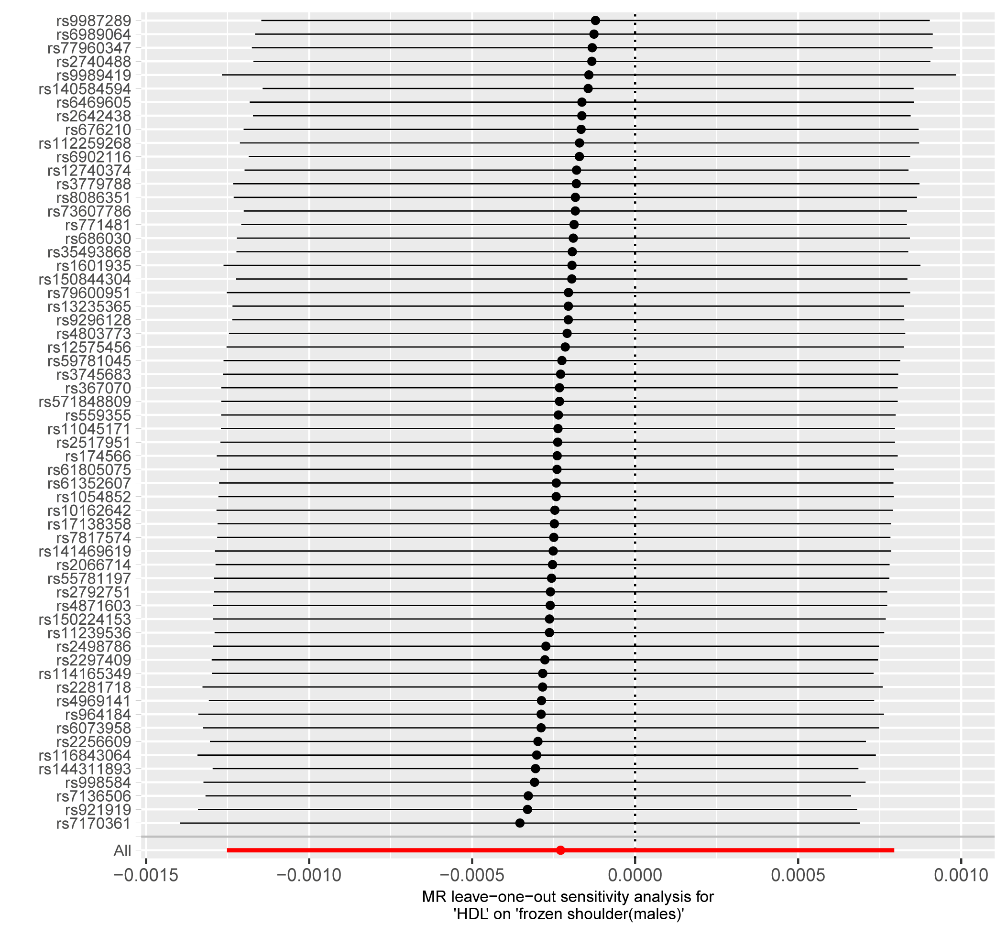


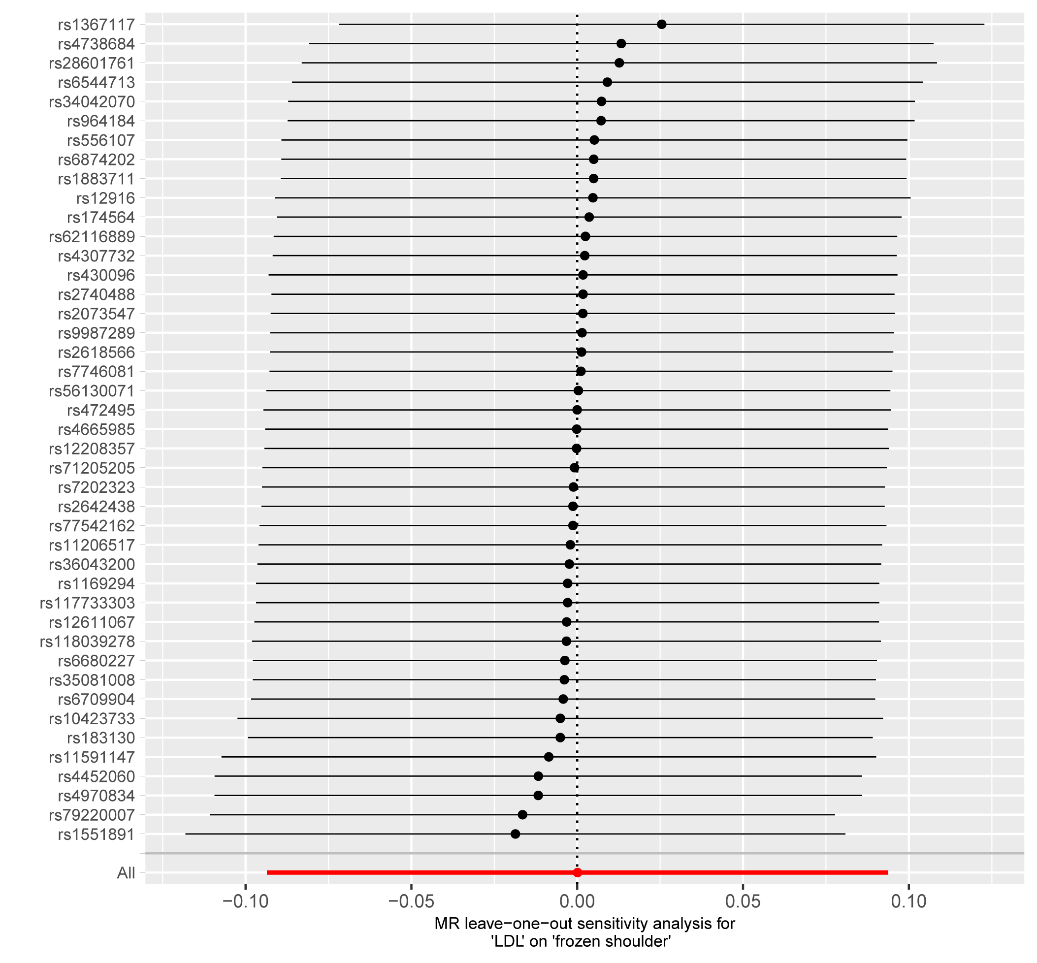

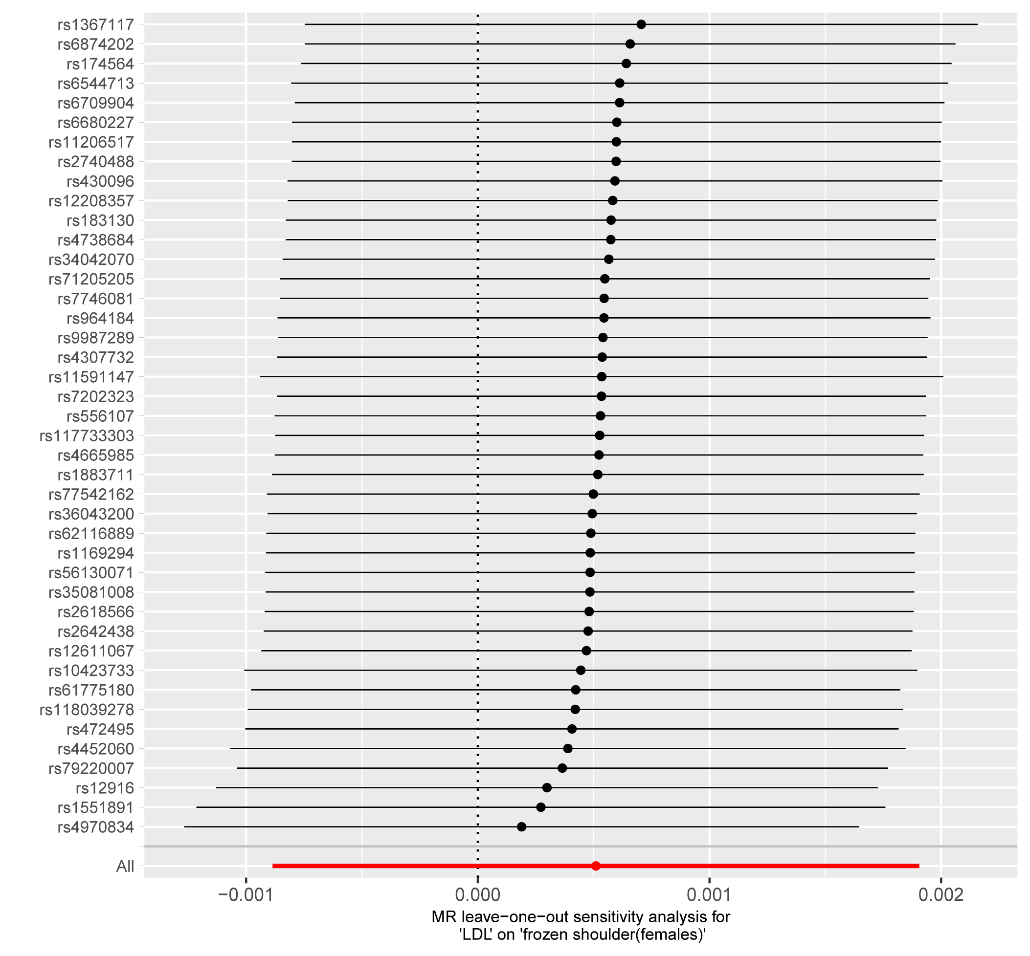


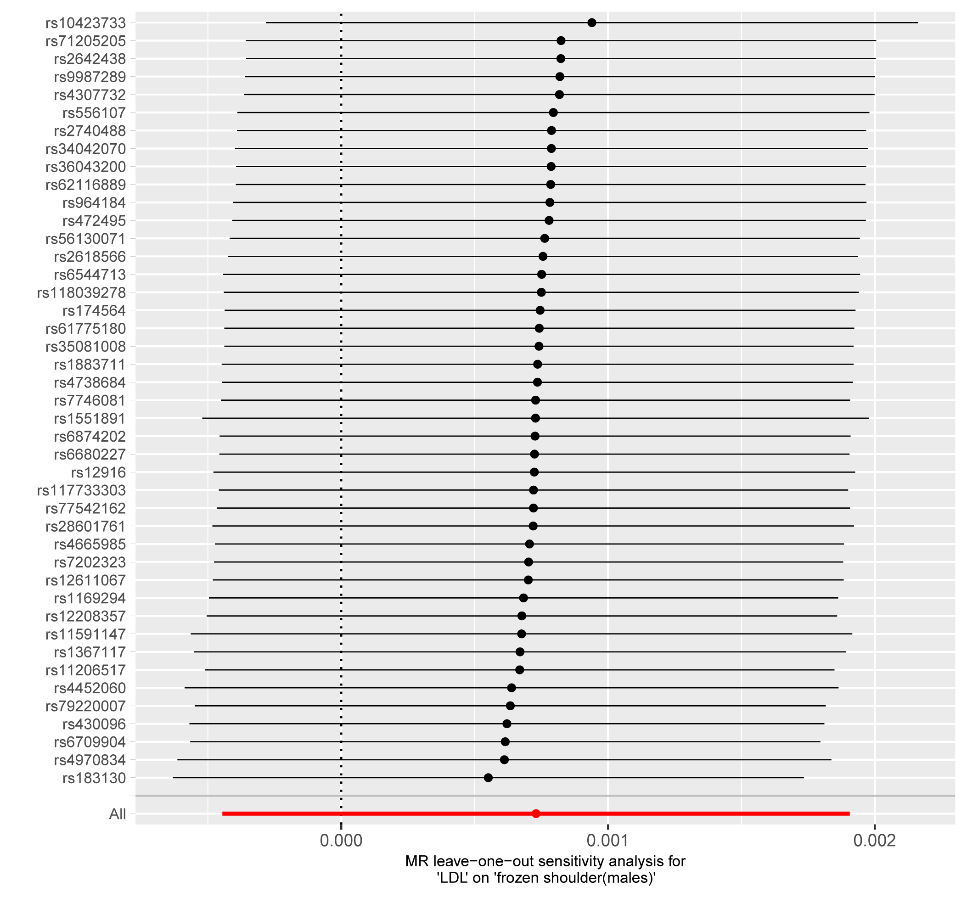

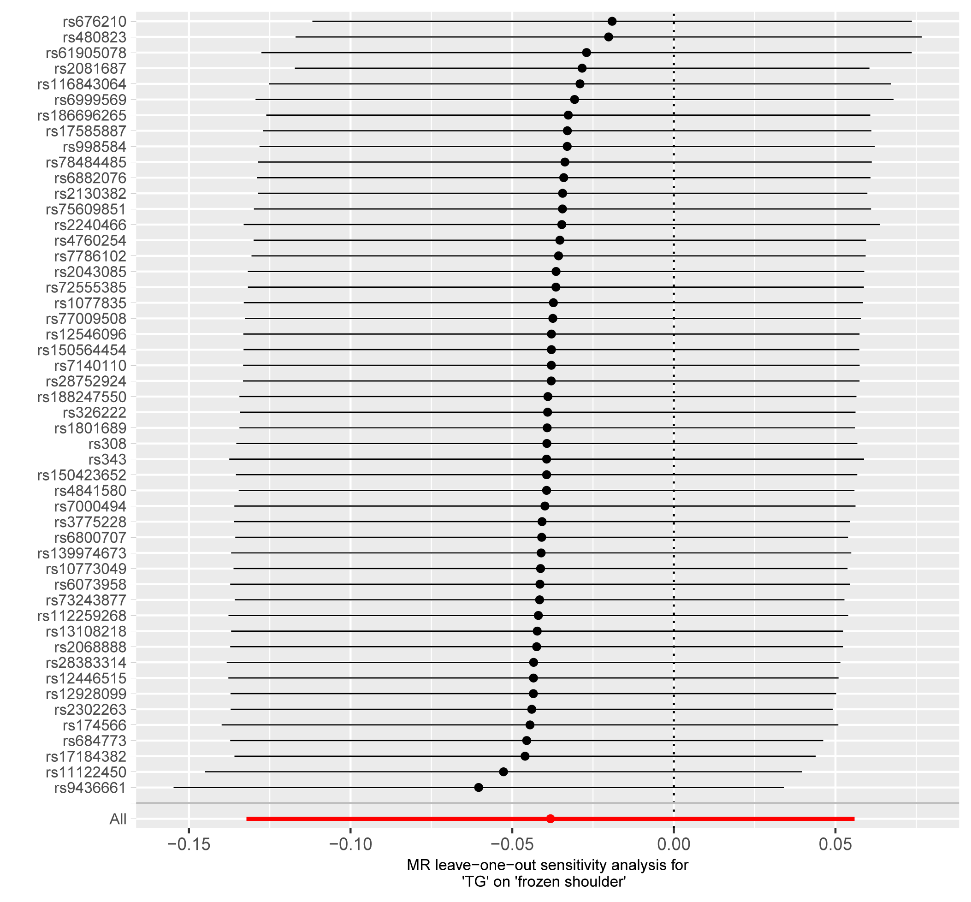


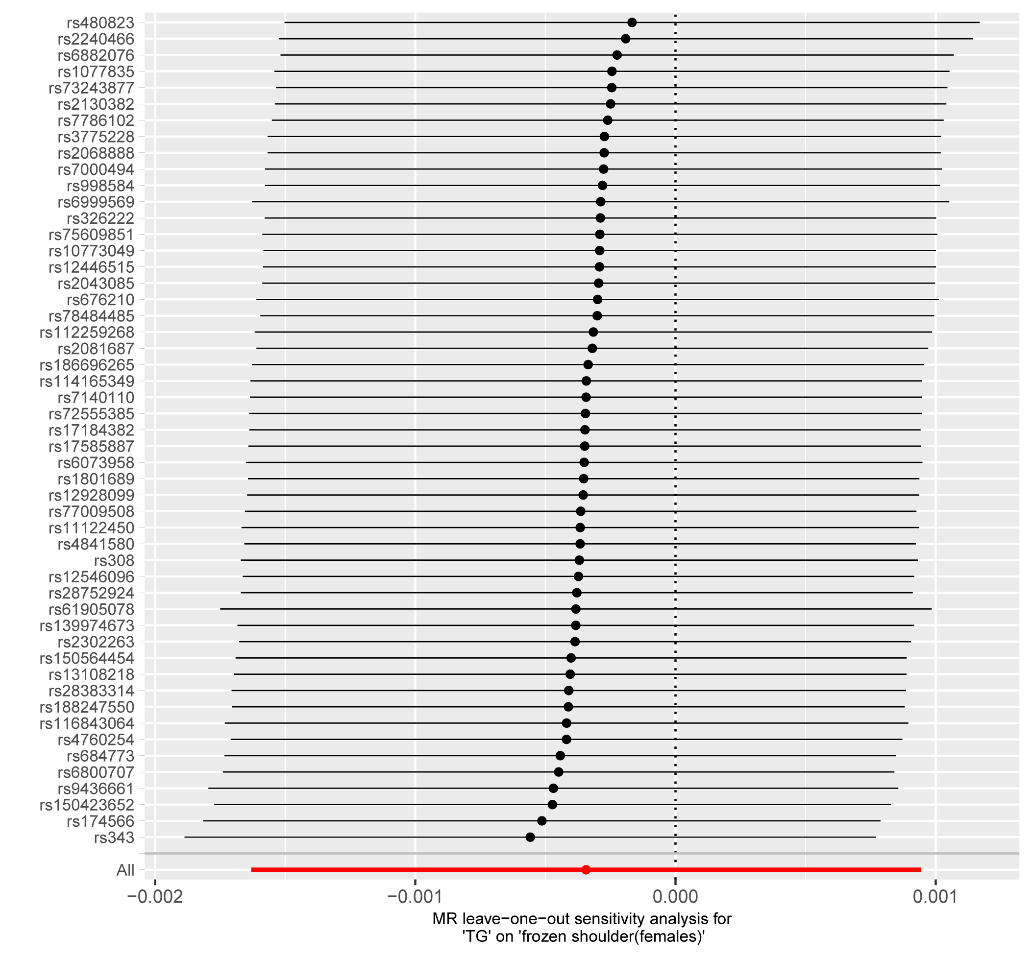

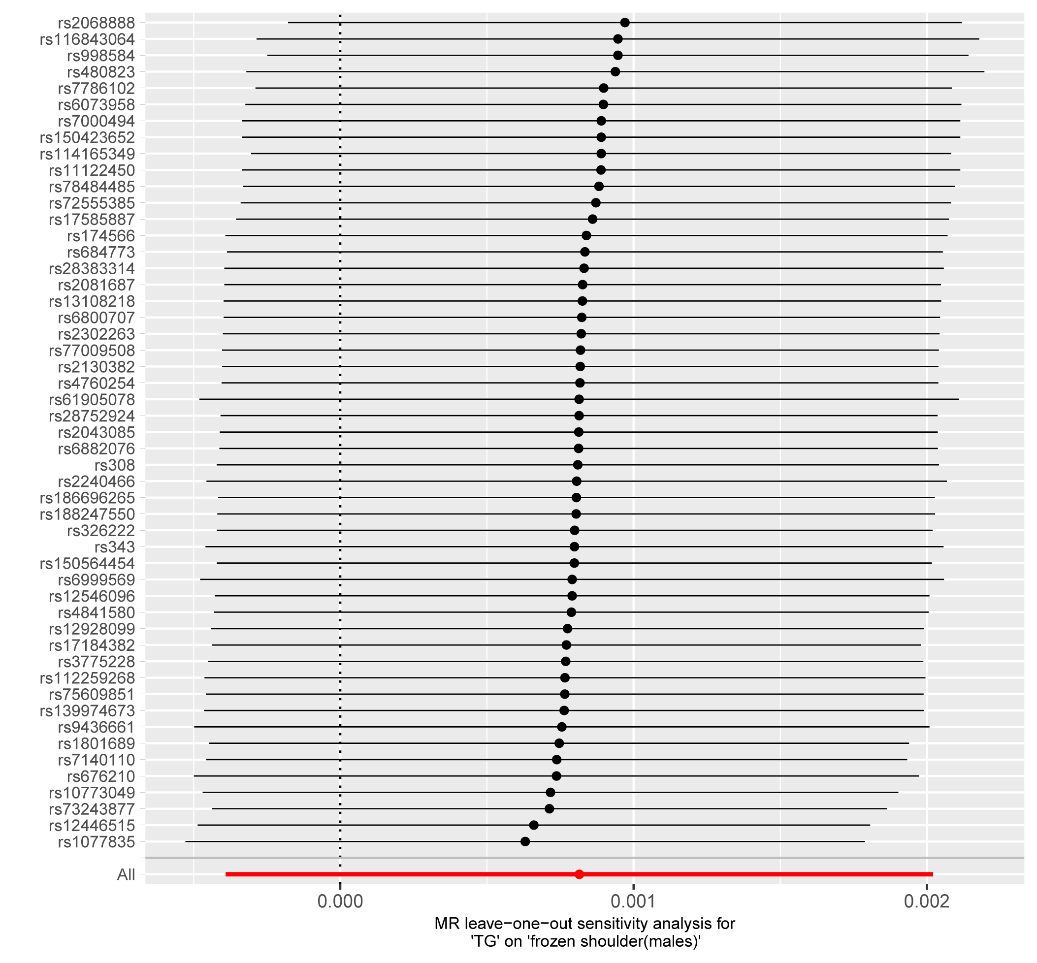


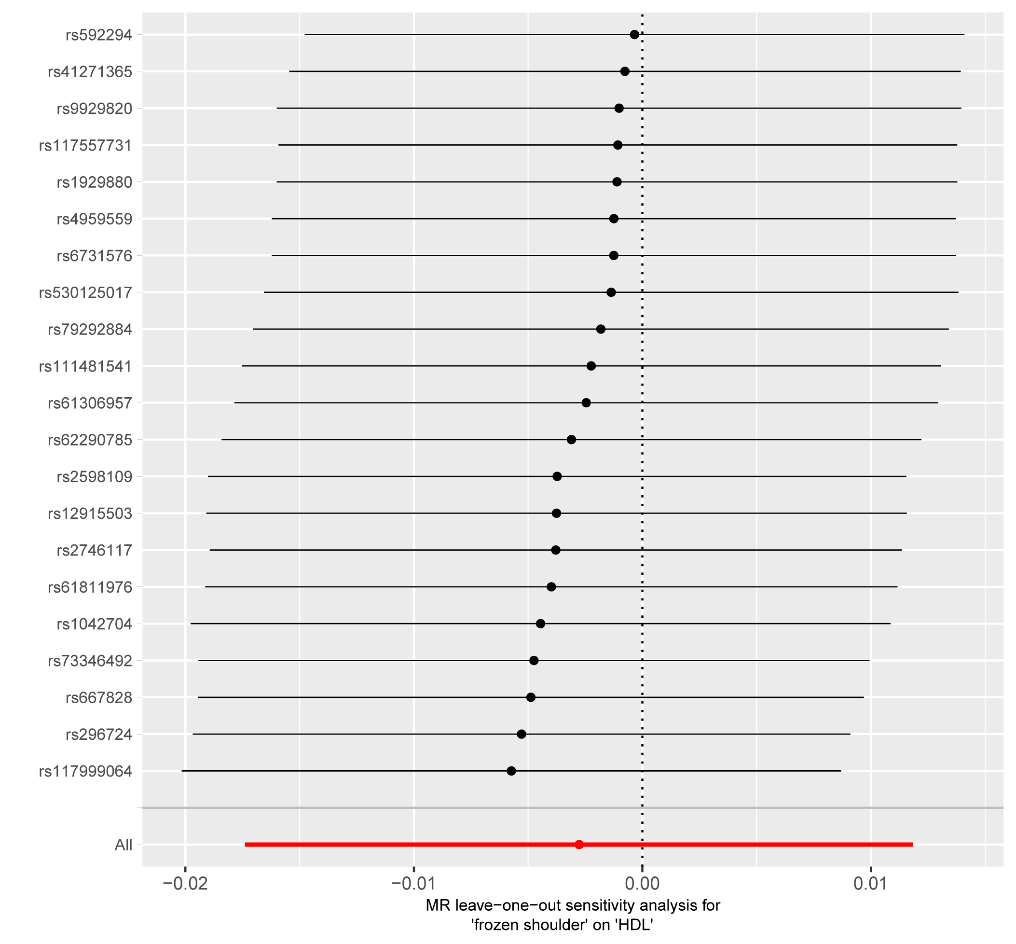

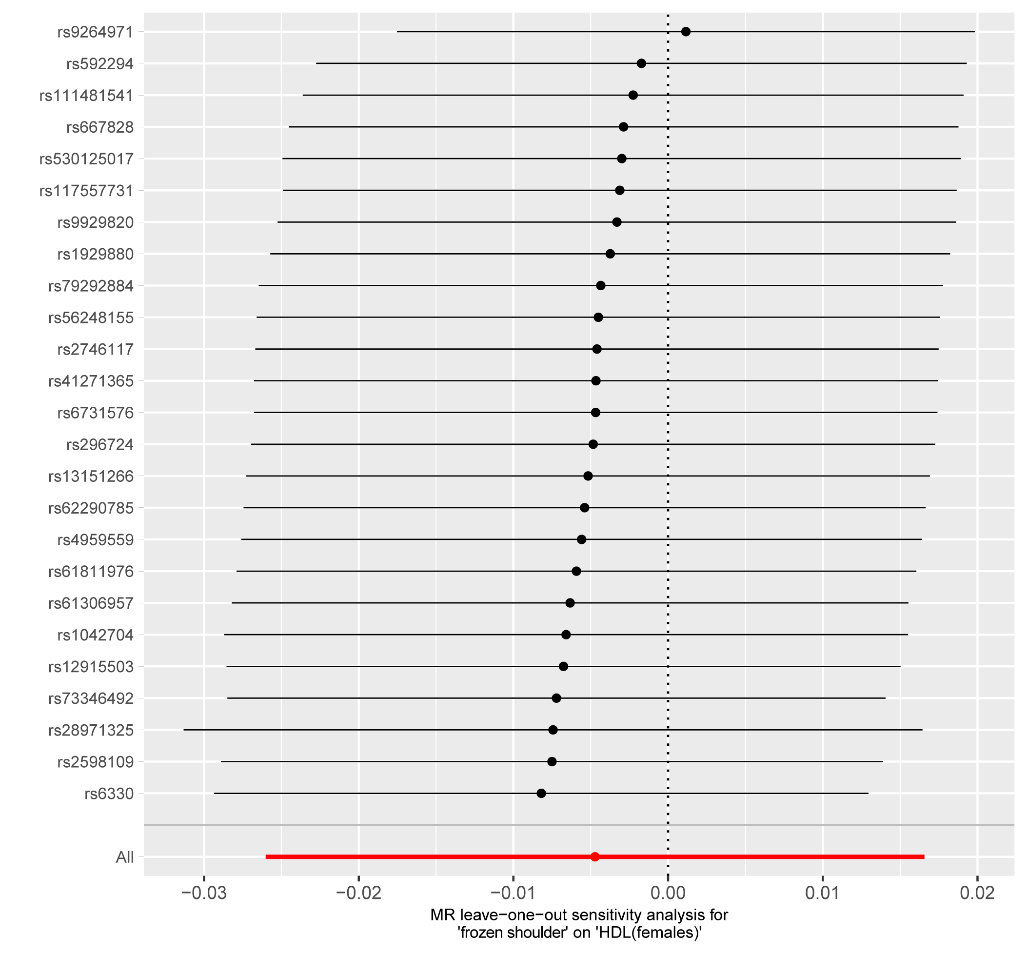


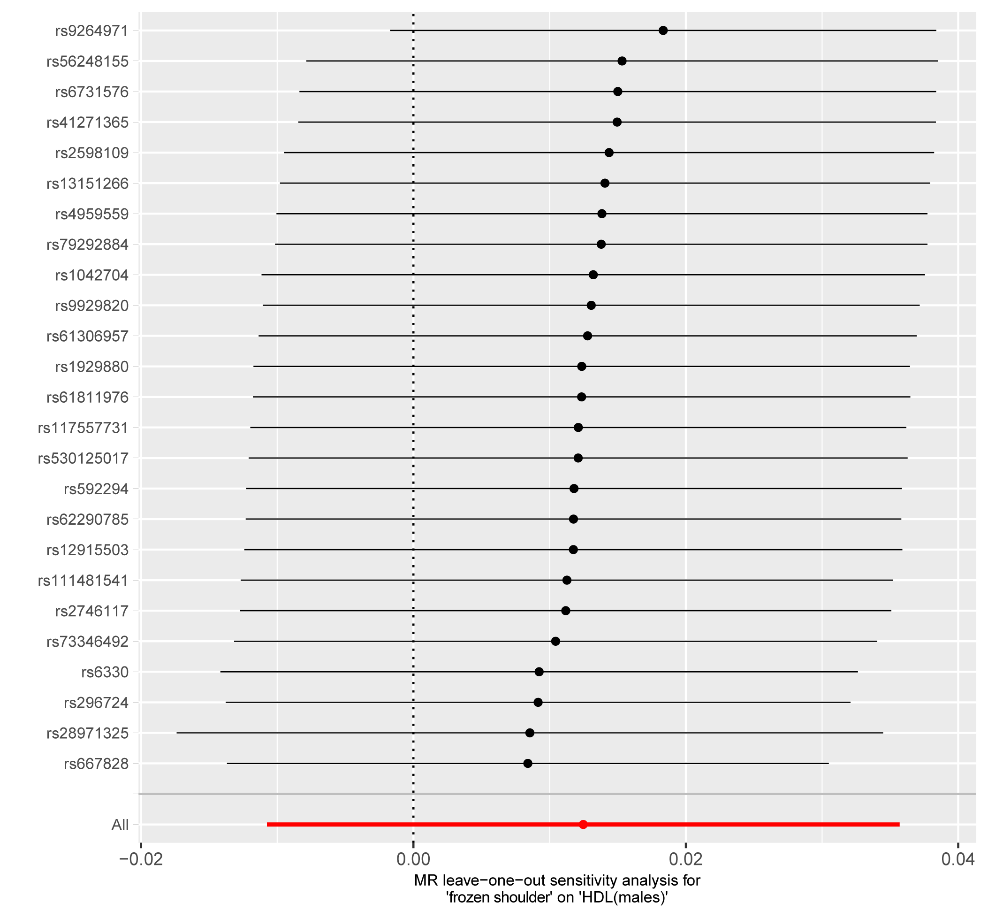

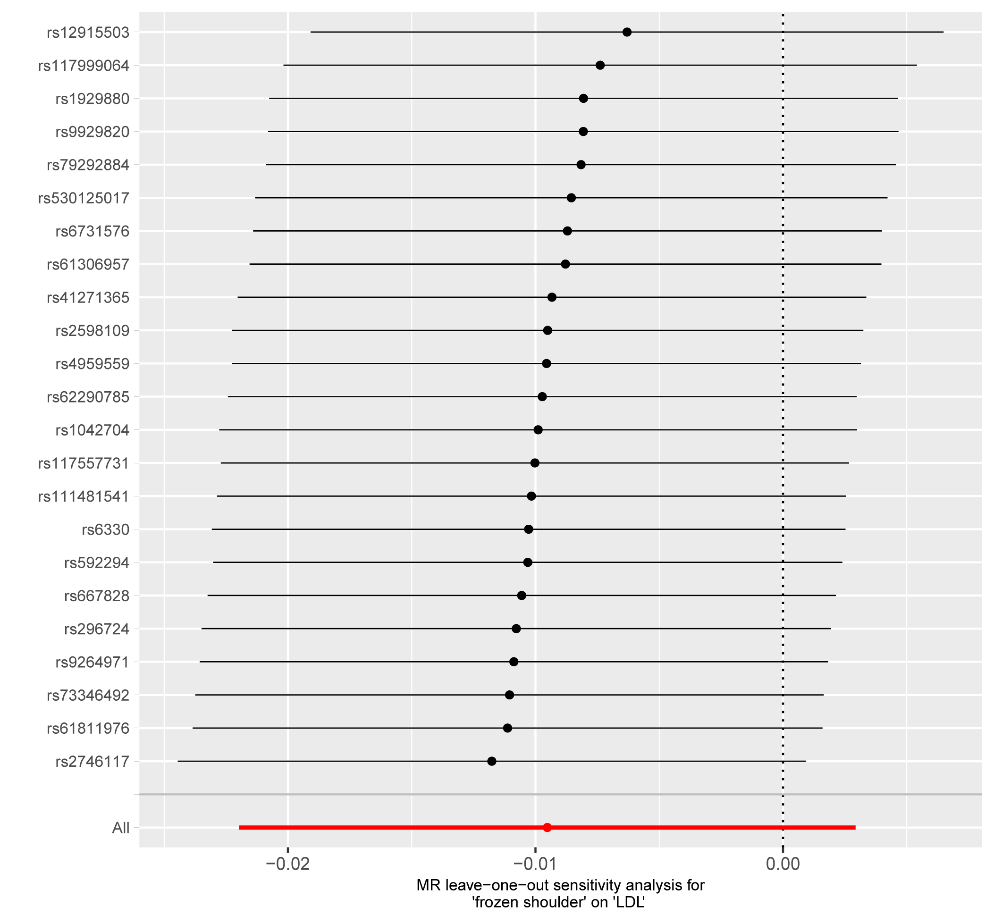


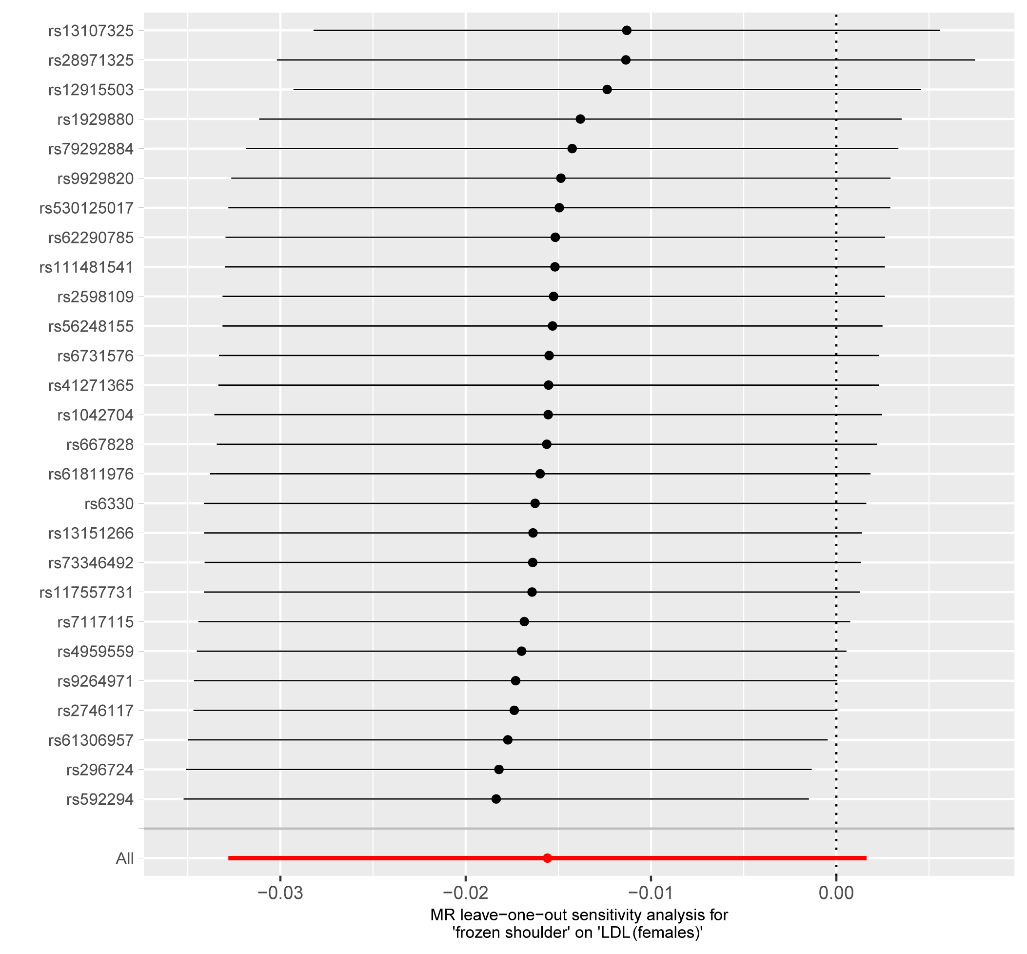

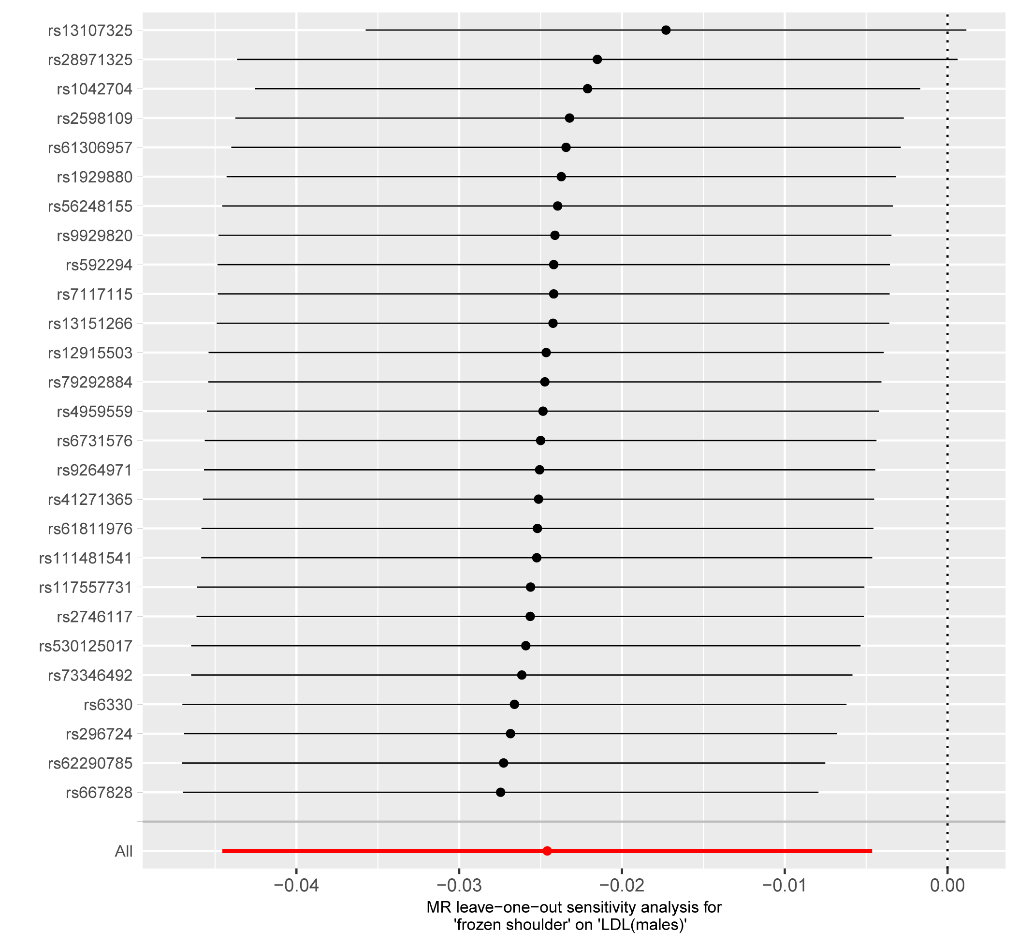

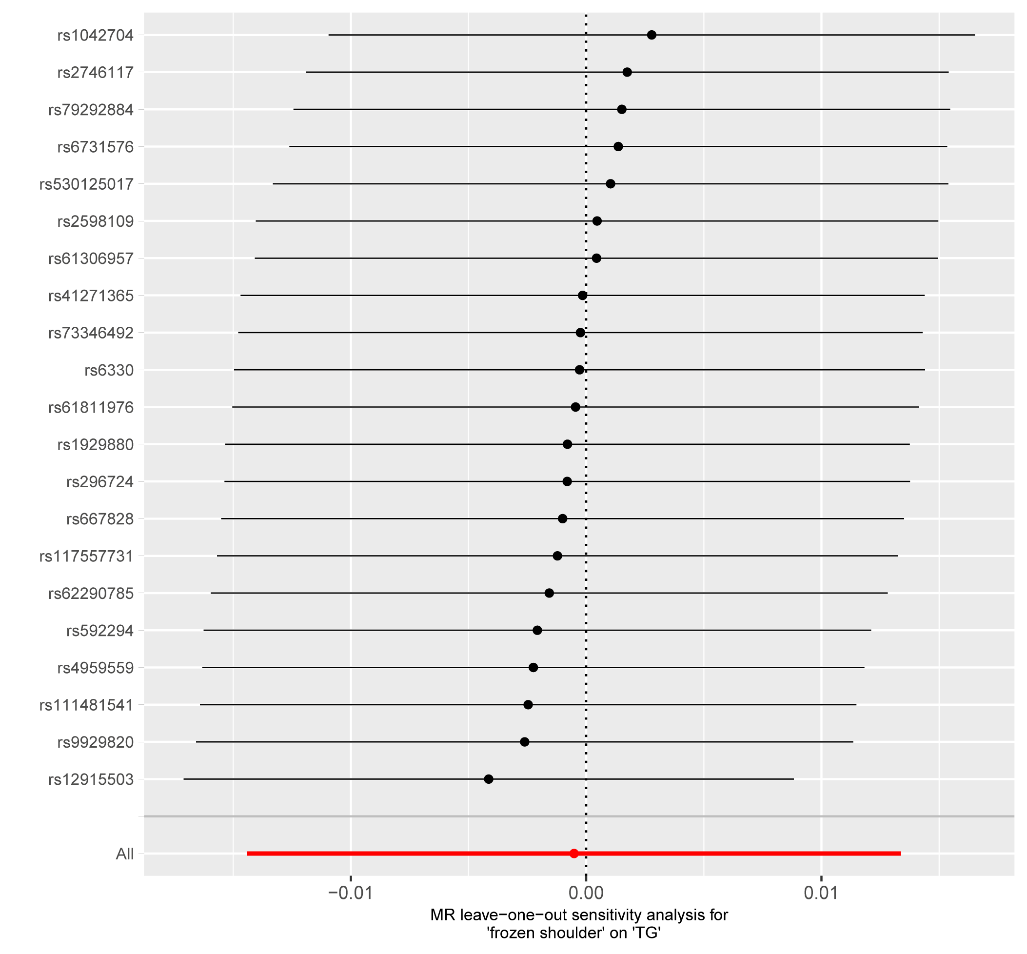

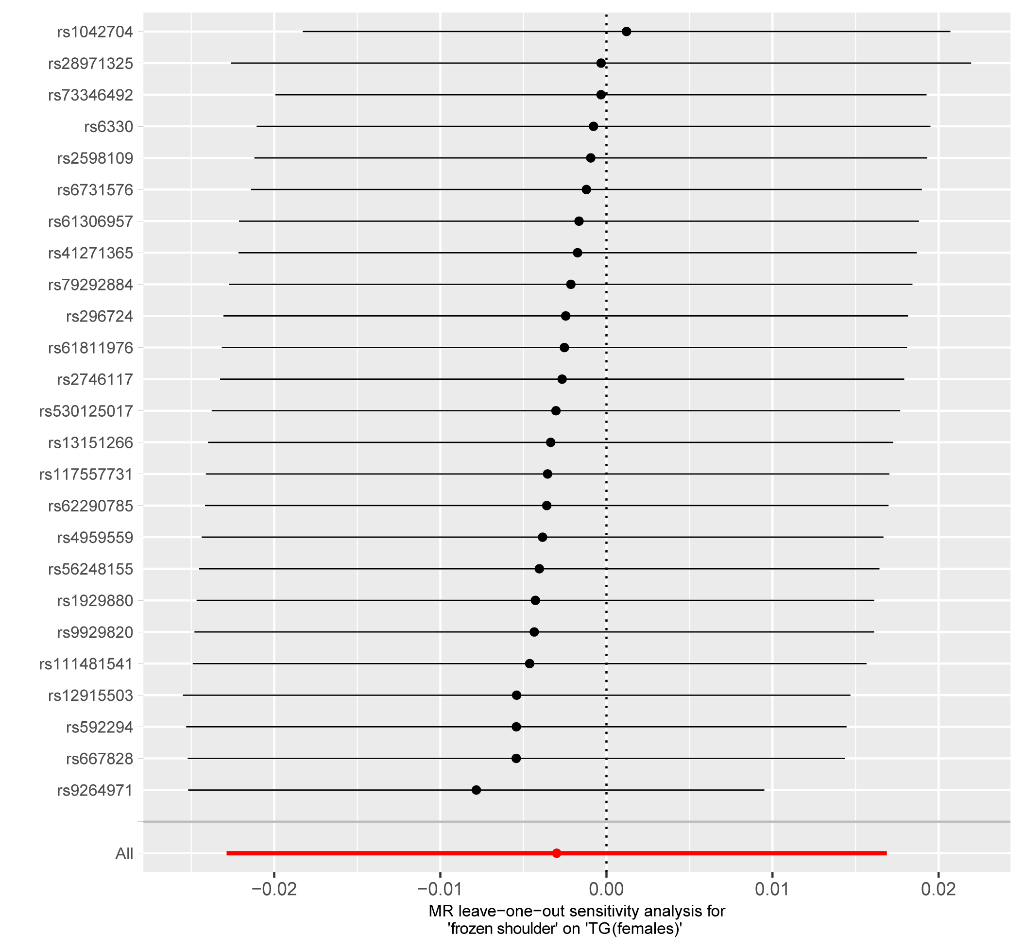

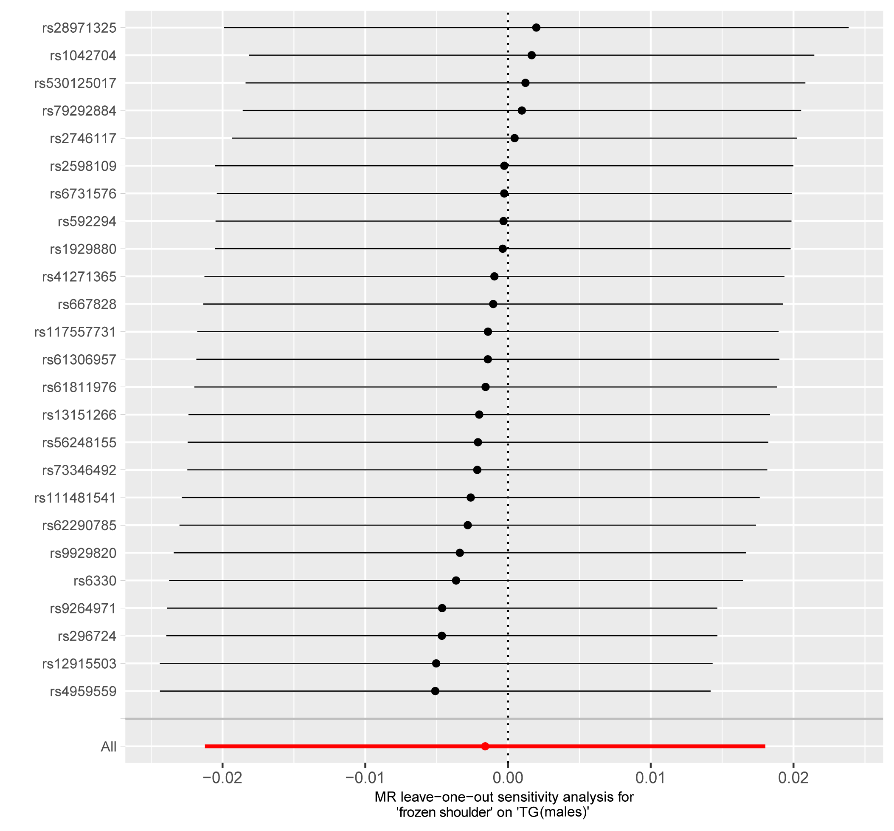


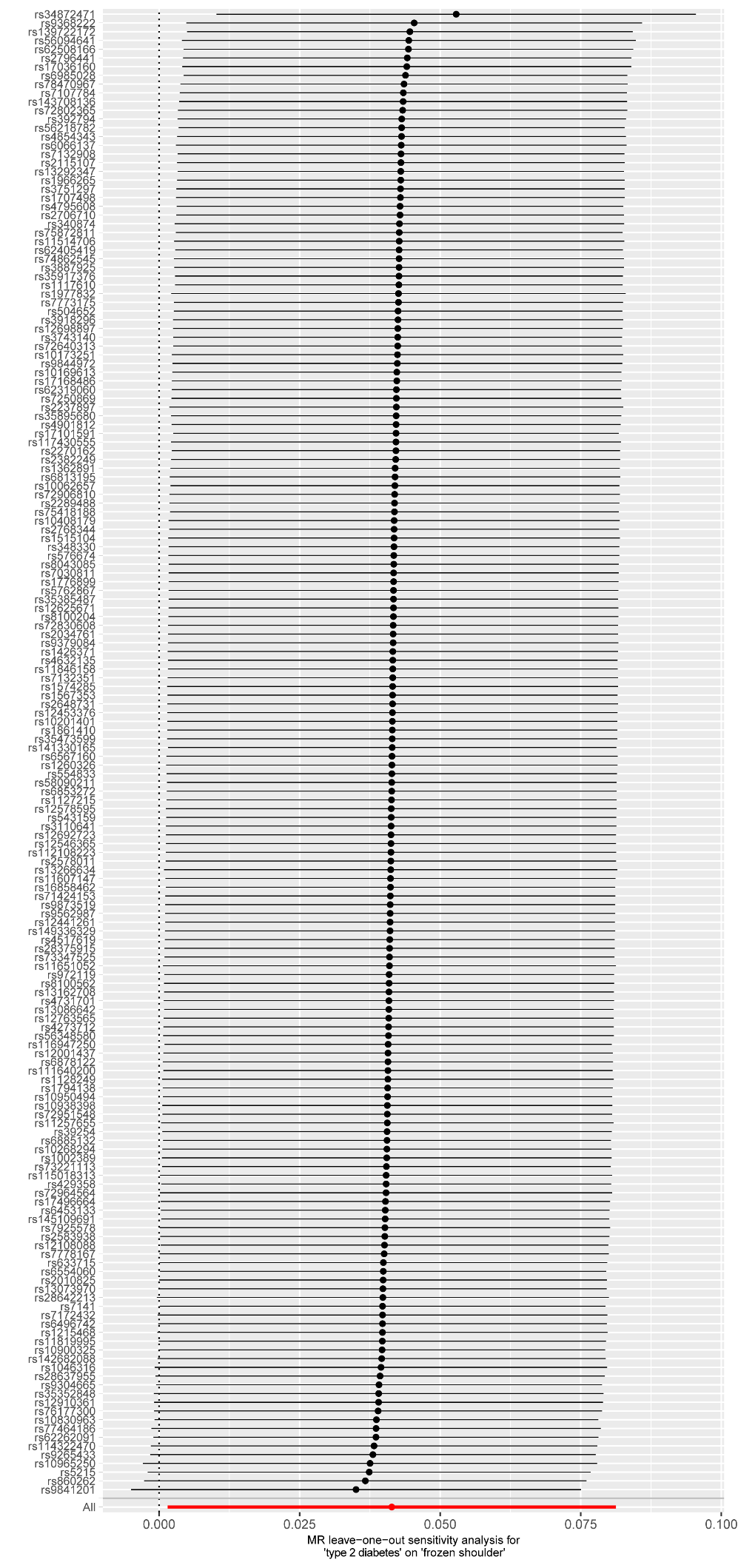

Supplement: Supplementary file 1 [file DataSheet1.docx]
